# Developing survival prediction models in colorectal cancer using epigenome-wide DNA methylation data from whole blood

**DOI:** 10.1038/s41698-024-00689-5

**Published:** 2024-09-06

**Authors:** Ziwen Fan, Dominic Edelmann, Tanwei Yuan, Bruno Christian Köhler, Michael Hoffmeister, Hermann Brenner

**Affiliations:** 1 Division of Clinical Epidemiology and Aging Research, German Cancer Research Center (DKFZ), Heidelberg, Germany; 2 Division of Biostatistics, German Cancer Research Center (DKFZ), Heidelberg, Germany; 3 Liver Cancer Center Heidelberg, Heidelberg University Hospital, Heidelberg, Germany; 4 Department of Medical Oncology, National Center for Tumor Diseases, Heidelberg University Hospital, Heidelberg, Germany; 5 NCT Heidelberg, National Center for Tumor Diseases (NCT) a partnership between DKFZ and University Hospital, Heidelberg, Germany; 6 Division of Preventive Oncology, German Cancer Research Center (DKFZ), Heidelberg, Germany; 7 German Cancer Consortium (DKTK), German Cancer Research Center (DKFZ), Heidelberg, Germany

**Keywords:** Prognostic markers, Cancer epigenetics, Colorectal cancer

## Abstract

While genome-wide association studies are valuable in identifying CRC survival predictors, the benefit of adding blood DNA methylation (blood-DNAm) to clinical features, including the TNM system, remains unclear. In a multi-site population-based patient cohort study of 2116 CRC patients with baseline blood-DNAm, we analyzed survival predictions using eXtreme Gradient Boosting with a 5-fold nested leave-sites-out cross-validation across four groups: traditional and comprehensive clinical features, blood-DNAm, and their combination. Model performance was assessed using time-dependent ROC curves and calibrations. During a median follow-up of 10.3 years, 1166 patients died. Although blood-DNAm-based predictive signatures achieved moderate performances, predictive signatures based on clinical features outperformed blood-DNAm signatures. The inclusion of blood-DNAm did not improve survival prediction over clinical features. M1 stage, age at blood collection, and N2 stage were the top contributors. Despite some prognostic value, incorporating blood DNA methylation did not enhance survival prediction of CRC patients beyond clinical features.

## Introduction

Colorectal cancer (CRC) is one of the most common cancers and one of the most common causes of cancer-related deaths globally, accounting for more than 9% of all cancer-related deaths^1^. The prognosis and therapy management of CRC rely on the TNM stage system, with a relative 5-year survival over 90% for localized-stage CRC but dropping below 15% for distant-stage CRC^2^. Nevertheless, the current TNM stage system is insufficient for accurately predicting survival and guiding clinical management, especially among stage II–III patients, resulting in potential over- or undertreatment^3,4^. Consequently, there is a growing need to establish more accurate novel prognostic signatures in predicting survival of CRC patients.

DNA methylation (DNAm) is a crucial epigenetic modification whose genome-wide analysis allows exploration of potentially valuable biomarkers for predicting prognosis in CRC^5–7^. Predictive signatures based on high-dimensional tumor DNAm, such as DNAm from resected tumor tissue and circulating tumor DNA (ctDNA)^8^, using machine-learning approaches have been increasingly proposed. However, the added value in discriminatory ability provided by tumor DNAm-derived signature to traditional clinical variables was unsatisfactory^9^. Additionally, it is not possible to examine the postoperative DNAm profile following the removal of the tumor. DNAm profiles from peripheral whole blood present alternative opportunities to develop predictive signatures and use them to monitor survival over an extended period. DNAm-based scores derived from peripheral whole blood, such as a DNAm mortality risk score and the age acceleration of PhenoAge and GrimAge, have been identified as strongly associated with all-cause mortality^10^. Given that these DNAm scores have been designed for the general population and not specific to CRC patient populations, their associations with CRC-specific mortality were weaker than their associations with all-cause mortality^11–13^. It is furthermore unclear whether and to what extent blood DNAm signatures that are specifically derived for predicting survival of CRC patients may add prognostic value to predictive models based on established prognostic clinical factors.

This study aimed to develop and evaluate blood-DNAm-based prognostic signatures in a large cohort of colorectal cancer patients recruited from a multi-site, population-based prospective study. Comprehensive clinical variables were available, and most blood samples were collected from 1-month before surgery to 1-year post-surgery. This design enabled us to assess the added value of DNAm profiles alongside clinical variables, particularly for monitoring survival after surgery. To minimize potential biases from rapid inflammatory changes shortly after surgery, and to address the clinical need for improved therapeutic decision-making in CRC patients with intermediate TNM stages (II–III), we created two specific subsets for further investigation. Subset 1 included patients whose blood was collected at least 1 month after surgery, and subset 2 focused on CRC patients with intermediate TNM stages. This approach allowed us to thoroughly examine the potential predictive value of blood DNAm in these critical patient groups. To ensure the robustness of the predictive signatures, we employed rigorous nested leave-sites-out cross-validation (nLSOCV) and eXtreme Gradient Boosting (XGBoost). These methods were applied to the total CRC cohort and the two subsets, assuring high reliability of the developed prognostic signatures.

## Results

### Characteristics of study population

The baseline characteristics of study population are summarized Table 1. Median age at CRC diagnosis and blood sample collection were 69 and 70 years, and a slight majority of patients were male (58.9%). The blood samples were collected ≥1-month after surgery in approximately half of all patients (49.2%, Supplementary Fig. 1) and from more than half of patients with intermediate TNM stages (TNM stage II–III, 67.8%). The tumor was located in the distal colon and rectum in 66.2% of patients. The majority of patients were never smokers (40.8%) or former smoker (43.2%). During a median follow-up of 10.3 years, 1166 patients died, of whom 595 died from CRC. We designed two subsets to investigate the potential predictive value of blood DNAm in patients whose blood was collected ≥1-month after surgery (subset 1, N = 1042) and CRC patients with intermediate TNM stages (subset 2, N = 1434). The distribution of characteristics was similar among two subsets and the total CRC cohort.

**Table 1:** Baseline characteristics of the study population

Characteristics	Total^a^	Subset 1^b^	*p*	Subset 2^c^	*p*
*N*	2116	1042	-	1434	-
Age at blood collection (year, median [IQR])	69 [62, 77]	69 [62, 76]	0·21	70 [62, 77]	0.38
Age at CRC diagnosis (year, median [IQR])	70 [63, 77]	70 [62, 77]	0·83	70 [63, 77]	0.38
Sex (female, %)	869 (41.1)	399 (38.3)	0·15	612 (42.7)	0.36
TNM Stage (%)			0.55		-
I	379 (17.9)	200 (19.3)		0 (0.0)	
II	738 (34.9)	370 (35.8)		738 (51.5)	
III	696 (32.9)	343 (33.1)		696 (48.5)	
IV	291 (13.8)	122 (11.8)		0 (0.0)	
Tumor Location (%)			0.44		0.44
Proximal colon	712 (33.6)	369 (35.5)		487 (34.0)	
Distal colon	766 (36.2)	381 (36.7)		549 (38.3)	
Rectum	635 (30.0)	289 (27.8)		396 (27.6)	
Family history of CRC (yes, %)	89 (4.2)	48 (4.6)	0.67	59 (4.1)	0.96
BMI (kg/m2, median [IQR])	26.1 [23.6, 29.0]	26.2 [23.7, 28.9]	0.99	26.1 [23.7, 29.0]	0.99
Education (years, %)			0.76		0.79
≤9	1456 (68.8)	708 (68.1)		1002 (69.9)	
10–11	347 (16.4)	166 (16.0)		237 (16.5)	
≥12	309 (14.6)	165 (15.9)		192 (13.4)	
Smoking status (%)			0.23		0.76
Never	863 (40.8)	385 (37.0)		608 (42.4)	
Former	915 (43.2)	482 (46.3)		607 (42.3)	
Current	336 (15.9)	174 (16.7)		217 (15.1)	
Alcohol consumption (g/d, median [IQR])	8.9 [2.0, 22.5]	9.3 [2.3, 22.0]	0.53	8.7 [2.0, 22.4]	0.82
Average lifetime physical activity (MET-h/wk, median [IQR])	190.7 [130.2, 278.3]	181.0 [121.2, 262.6]	0.006	189.2 [130.9, 275.1]	0.74
Blood collection time (≥1-month post-surgery, %)	1042 (49.2)	1042 (100.0)	-	713 (49.7)	-
Median follow-up time (months [95% CI])	123 (123–125)	119 (118–121)	-	124 (122–126)	-
Deaths (CRC-specific deaths)	1166 (595)	534 (254)	-	735 (314)	-

Distributions of total patients and each subset were compared with Pearson’s chi-square test or Kruskal–Wallis test.

*IQR* interquartile range, *CI* confidence interval, *BMI* body mass index, *MET-h/week* metabolic equivalent hours/week, *CRC* colorectal cancer.

^a^Numbers do not add up to 2116 because of missing values in the following variables (number): BMI (10), pack-years (17), alcohol consumption (7), average lifetime physical activity (40).

^b^Subset 1 includes patients with blood samples collected ≥1-month after surgery.

^c^Subset 2 includes patients with TNM stage II–III.

### Model performance

**Fig. 1:**
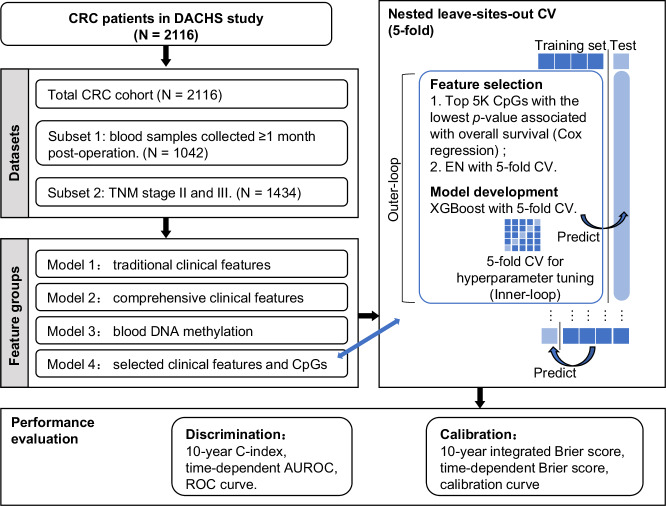
Study design and workflow. Prognostic predictive signatures for survival of CRC patients were developed using three datasets: the total CRC cohort and two subsets stratified by blood collection time and TNM stage. Predictive models were developed in each dataset based on four feature groups using 5-fold nested leave-sites-out cross-validation. In each outer-loop, the training set for Model 1 used traditional clinical features including age, sex, and TNM stages. The training sets for Models 2 and 3 underwent feature selection with their corresponding feature groups. The training set for Model 4 incorporated features selected from Models 2 and 3. Selected features were used to construct predictive signatures using XGBoost, with hyperparameter tuning conducted in inner-loops. Performance evaluation involved discrimination and calibration indicators, aggregating results from each test set in the outer loops. Abbreviation: CRC colorectal cancer, CV cross-validation, EN elastic net, XGBoost eXtreme Gradient Boosting.

Predictive models for survival of CRC patients were developed using XGBoost with a 5-fold nLSOCV (Fig. 1) scheme across four feature groups: *Model 1*: traditional clinical features including TNM stage, *Model 2*: comprehensive clinical features including major tumor markers, *Model 3*: blood-DNAm, and *Model 4*: the inclusion of blood-DNAm with comprehensive clinical features. These four models were developed based on three datasets: the total CRC patient cohort, subset 1, and subset 2.

Table 2 shows the performance of predictive models for overall survival of CRC patients trained on the total CRC cohort (N = 2116). At 1-, 3-, 5-, and 10-year follow-ups, 187 (8.8%), 499 (23.6%), 716 (33.8%), and 1087 (51.4%) of the patients had died in the total CRC cohort. Although Model 3, which was based on blood-DNAm, had some predictive value with a 10-year concordance index (C-index) of 0.64 and time-dependent areas under the receiver operating characteristics curve (AUROCs) ranging from 0.67 to 0.71, it performed worse compared to Models 1 and 2. Models 1 and 2, which were both based on clinical features, achieved higher 10-year C-indexes (0.74–0.75) and AUROCs at all time points (0.78–0.83). Model 4, which combined blood-DNAm with comprehensive clinical features, showed similar performance to Models 1 and 2, with a 10-year C-index of 0.75 and AUROCs ranging from 0.80 to 0.84. The statistical tests in each outer-loop supported these comparisons (Supplementary Table 1). These findings indicate that adding blood DNAm did not improve the prognostic performance compared to models based solely on clinical features. The Kaplan-Meier (KM) curves for the dichotomized signature of each model in the total CRC cohort are displayed in Supplementary Fig. 2. The prognostic performance, time-dependent ROC curve, and time-dependent calibration curve of the models in each outer-loop of the total CRC cohort are displayed in Supplementary Table 2 and Supplementary Figs. 3, 4.

**Table 2:** Performance and calibration of predictive models for overall survival in the total CRC cohort (N = 2116)

Models	C-index (10 y)	Time-dependent AUROC				IBS (10 y)	Time-dependent Brier score			
		1 y	3 y	5 y	10 y		1 y	3 y	5 y	10 y
Model 1	0.74 (0.72–0.75)	0.81 (0.78–0.88)	0.80 (0.77–0.82)	0.78 (0.76–0.82)	0.79 (0.78–0.81)	0.15 (0.14–0.15)	0.07 (0.06–0.09)	0.14 (0.12–0.15)	0.17 (0.15–0.19)	0.19 (0.18–0.19)
Model 2	0.75 (0.74–0.77)	0.83 (0.80–0.88)	0.82 (0.79–0.86)	0.81 (0.78–0.84)	0.82 (0.80–0.83)	0.14 (0.13–0.15)	0.07 (0.06–0.08)	0.13 (0.12–0.15)	0.16 (0.14–0.18)	0.18 (0.17–0.18)
Model 3	0.64 (0.63–0.65)	0.71 (0.67–0.74)	0.67 (0.66–0.69)	0.67 (0.66–0.69)	0.67 (0.63–0.69)	0.18 (0.17–0.19)	0.08 (0.06–0.09)	0.17 (0.16–0.19)	0.21 (0.20–0.22)	0.23 (0.22–0.24)
Model 4	0.75 (0.74–0.77)	0.84 (0.82–0.87)	0.83 (0.80–0.86)	0.81 (0.78–0.84)	0.80 (0.78–0.82)	0.14 (0.13–0.15)	0.07 (0.06–0.08)	0.13 (0.12–0.15)	0.16 (0.14–0.18)	0.18 (0.17–0.19)

Model 1 based on traditional clinical features, Model 2 based on comprehensive clinical features, Model 3 based on blood DNA methylation, Model 4 combined both clinical features and blood DNA methylation. The values are presented as the mean (range) across 5-fold outer-loops.

*AUROC* area under receiver operating characteristic curve, *IBS* integrated Brier score.

Table 3 shows the performance of predictive models for overall survival of CRC patients trained on subset 1 (N = 1042) and subset 2 (N = 1434). At 1-, 3-, 5-, 10-year follow-ups, 80 (7.7%), 223 (21.4%), 321 (30.8%), and 501 (48.1%) patients, respectively, had died in subset 1, while in subset 2, the corresponding numbers were 80 (5.6%), 254 (17.7%), 404 (28.2%), and 672 (46.9). Among patients with intermediate stage, the 10-year AUROCs for Model 1, 2 and 4 were lower, ranging from 0.69 to 0.75, compared to the total CRC cohort, ranging from 0.79 to 0.82. Similar performance patterns were observed in both subsets as in the total CRC cohort, where adding blood DNAm to comprehensive clinical features did not significantly improve the predictive performance over traditional clinical features. The KM curves for dichotomized signature of each model in subset 1 are displayed in Supplementary Fig. 5, the comparison of prognostic performances, performance and calibrations, time-dependent ROC curve, and time-dependent calibration curve of the models in each outer-loop of subset 1 are displayed in Supplementary Tables 3, 4, and Supplementary Figs. 6, 7. Correspondingly, those in each outer-loop of subset 2 are displayed in Supplementary Tables 5, 6, and Supplementary Figs. 8–10.

**Table 3:** Performance and calibration of predictive models for overall survival in the two subsets

Subsets	Models	C-index (10 y)	Time-dependent AUROC				IBS (10 y)	Time-dependent Brier score			
			1 y	3 y	5 y	10 y		1 y	3 y	5 y	10 y
Subset 1^a^ (N = 1042)	Model 1	0.74 (0.72–0.75)	0.82 (0.72–0.91)	0.80 (0.76–0.83)	0.78 (0.75–0.82)	0.78 (0.75–0.79)	0.14 (0.14–0.16)	0.06 (0.06–0.07)	0.13 (0.11–0.16)	0.17 (0.15–0.19)	0.20 (0.19–0.20)
	Model 2	0.75 (0.74–0.76)	0.84 (0.79–0.89)	0.82 (0.79–0.85)	0.80 (0.76–0.82)	0.79 (0.78–0.80)	0.14 (0.13–0.15)	0.06 (0.06–0.07)	0.13 (0.11–0.15)	0.16 (0.15–0.18)	0.19 (0.18–0.20)
	Model 3	0.64 (0.61–0.66)	0.71 (0.65–0.78)	0.67 (0.62–0.71)	0.65 (0.62–0.67)	0.68 (0.66–0.71)	0.17 (0.16–0.18)	0.07 (0.06–0.08)	0.16 (0.13–0.19)	0.20 (0.18–0.22)	0.23 (0.22–0.24)
	Model 4	0.74 (0.73–0.75)	0.86 (0.82–0.91)	0.83 (0.78–0.87)	0.79 (0.77–0.83)	0.78 (0.76–0.80)	0.14 (0.13–0.15)	0.06 (0.05–0.06)	0.12 (0.10–0.14)	0.16 (0.14–0.18)	0.19 (0.18–0.20)
Subset 2^b^ (N = 1434)	Model 1	0.67 (0.66–0.68)	0.75 (0.68–0.84)	0.69 (0.67–0.72)	0.69 (0.65–0.71)	0.73 (0.71–0.73)	0.16 (0.14–0.17)	0.05 (0.04–0.07)	0.14 (0.12–0.16)	0.19 (0.17–0.21)	0.21 (0.21–0.22)
	Model 2	0.70 (0.68–0.72)	0.78 (0.71–0.84)	0.73 (0.69–0.76)	0.72 (0.68–0.75)	0.75 (0.75–0.76)	0.15 (0.13–0.16)	0.05 (0.03–0.06)	0.13 (0.11–0.16)	0.18 (0.16–0.20)	0.20 (0.20–0.21)
	Model 3	0.63 (0.60–0.67)	0.70 (0.68–0.75)	0.65 (0.60–0.69)	0.65 (0.60–0.67)	0.65 (0.59–0.70)	0.17 (0.15–0.20)	0.05 (0.04–0.07)	0.15 (0.12–0.19)	0.20 (0.17–0.25)	0.25 (0.22–0.27)
	Model 4	0.66 (0.64–0.69)	0.74 (0.69–0.82)	0.70 (0.64–0.74)	0.69 (0.63–0.72)	0.69 (0.64–0.73)	0.17 (0.14–0.19)	0.05 (0.04–0.07)	0.15 (0.12–0.18)	0.20 (0.17–0.24)	0.23 (0.21–0.25)

Model 1 based on traditional clinical features, Model 2 based on comprehensive clinical features, Model 3 based on blood DNA methylation, Model 4 combined both clinical features and blood DNA methylation. The values are presented as the mean (range) across 5-fold outer-loops.

*AUROC* area under receiver operating characteristic curve, *IBS* integrated Brier score.

^a^Subset 1 includes patients with blood samples collected ≥1-month after surgery.

^b^Subset 2 includes patients with TNM stage II–III.

Supplementary Table 7 shows the performances and calibrations of four predictive models for CRC-specific survival in the total CRC cohort and two subsets. Similar comparison patterns to overall survival were observed for CRC-specific survival in the total CRC cohort and the two subsets, and no improvements in performance were observed by adding blood DNAm to clinical features for the prediction of CRC-specific survival. The Kaplan-Meier (KM) curves for the dichotomized signature of each model in the total CRC cohort and the two subsets are displayed in Supplementary Figs. 11–13. The comparison of prognostic performances, performance, and calibrations of the models in each outer-loop of the total CRC cohort and two subsets are displayed in Supplementary Tables 8–13.

### Model interpretation and feature importance

**Fig. 2:**
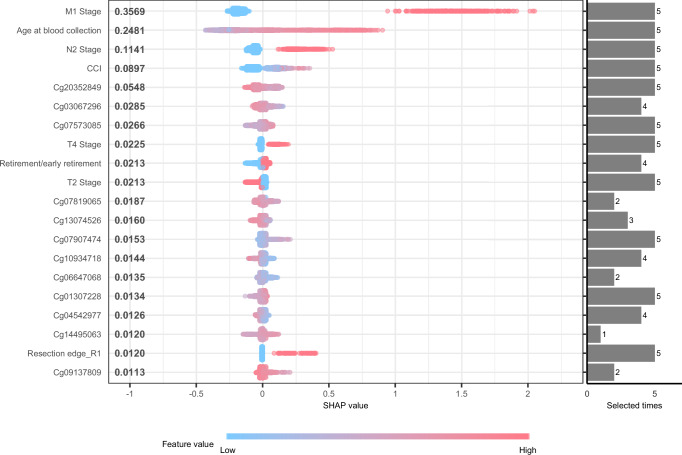
SHAP feature importance analysis. The SHAP analysis is based on a model that incorporates blood DNA methylation along with comprehensive clinical features in the total CRC cohort. On the Y-axis, features are listed in descending order of importance (from top to bottom). On the X-axis, the left represents the SHAP value, and the right shows the number of times features were selected in the outer loops. Colors represent feature values, with red indicating high values and blue indicating low values. Each point corresponds to an individual patient. For features such as the M1 stage, patients with the M1 stage (red) contribute to a high SHAP value, indicating an elevated risk of death. Abbreviations: CCI Charlson Comorbidity Index, SHAP SHapley Additive exPlanations.

Figure 2 displays the top 20 features contributing to the prognostic signature based on Model 4, which combined both comprehensive clinical features and blood DNAm, in the total CRC cohort. The SHAP (SHapley Additive exPlanation) analysis suggests that M1 stage, age at blood collection, and N2 stage were the top contributors to overall survival prediction in CRC patients. The Charlson comorbidity index (CCI) and cg20352849 exhibited much higher contributions compared to T4 and T2 stages. Additionally, cg03067296 and cg07573085, along with retirement/early retirement status, showed similar contributions to the T4 and T2 stages.

## Discussion

In this multi-site large population-based prospective cohort study, incorporating comprehensive clinical features and DNAm data from peripheral whole blood, we found that the inclusion of these features did not lead to a significant enhancement of predictive signatures for survival in CRC patients when compared to those developed with traditional clinical variables only, including age, sex, and TNM stage. M1 stage, age at blood collection, and N2 stage emerged as the top contributors to overall survival prediction in CRC patients. Similar performance of predictive signatures was observed in patients whose blood samples were taken ≥1-month post-surgery and in those with intermediate TNM stages.

Interest in identifying prognostic DNAm biomarkers for survival in CRC patients has seen a steep rise, with the hope that biomarkers derived from novel omics-technologies may hold potential as valuable supplements to established prognostic criteria; however, there is still insufficient evidence to establish their utility in clinical practice^5^. A recent systematic review and external validation have highlighted the insufficient performance and limited generalizability of published prognostic DNAm biomarkers derived from tumor tissue^9^. These limitations could be attributed to relatively small sample sizes, improper handling of missing data, and a lack of evaluation of calibration. In addition, for most of the machine-learning-based epigenome-wide research, a robust method to validate models, such as LSOCV and external validation^14^, has not been applied^15^, an observation that was also made in studies concerning ctDNA and tumor methylation^8,9,15,16^. To our knowledge, no prior study has investigated the prognostic value of DNAm signatures derived from peripheral whole blood in CRC patients. Additionally, there are no available public DNAm datasets that provide DNA methylation array data from whole blood samples for external validation^17^. Most prior studies have focused on ctDNA^8,18–22^, with only one study examining FOXO3 blood DNA methylation, which found no association between FOXO3 CpGs and survival in CRC patients^23^.

In the current study, we developed prognostic signatures using DNAm profiles from peripheral whole blood, integrating comprehensive clinical features to assess the added value of blood DNAm compared to traditional clinical features on model performance. To ensure unbiased generalizability evaluation, we investigated a cohort with a large sample size and employed a nested LSOCV strategy, iteratively training and validating the model on different sites, maintaining independent training and testing datasets. This nested LSOCV approach simulated real-world scenarios, offering an optimal bias-variance trade-off and leveraging the full richness of data during training, including out-of-distribution samples for testing sites. The prognostic signature based on blood DNAm alone showed insufficient performance, with a time-dependent AUROC ranging from 0.67-0.71 for either short-term or long-term follow-up, and provided no added value beyond traditional clinical features, including age, sex, and TNM stage. Similarly, tumor DNAm showed poor performance in the aforementioned systemic review and external validation study^9^, while the effectiveness of ctDNA methylation is still unclear. An improvement in performance from combining ctDNA methylation with clinical features over TNM staging was noted; however, as this combination was not directly compared to clinical features alone, the source of the improvement—whether clinical features or ctDNA methylation—remains unclear^8^. Additionally, we explored prognostic signatures in CRC patients with intermediate TNM stage, requiring further risk stratification due to the survival paradox dilemma^3,4,24^. The predictive value of blood-DNAm profiles remained poor, offering no added value compared to traditional clinical features, and furthermore, it even decreased the discriminatory capability when combined with comprehensive clinical features.

Examining DNAm profiles in peripheral whole blood, spanning from pre-surgery to post-surgery periods, presents a unique opportunity to explore their potential as a tool for monitoring postoperative CRC prognosis. The comparable (albeit rather limited) discriminative performance of predictive signatures based on blood DNAm was evident across both the total CRC cohort and postoperative CRC patients, indicating that DNAm may maintain its predictive capability even after surgical tumor removal. While blood-based DNAm profiles do change post-surgery and during adjuvant therapy ^25,26^, suggesting their potential inclusion in predicting CRC response to therapy^7,27^, the number of patients with DNAm profiles in the current study determined during specific treatment windows, such as the period between surgery and chemotherapy, was limited. This hindered more differentiated assessment of when the prognostic value of blood DNAm might be most notable during treatment. Further research in large cohorts of CRC patients undergoing repeat longitudinal blood sampling is needed to address this important point. Additionally, considering that tumor recurrence may alter blood DNAm profiles, developing a signature for timely monitoring could offer early detection of CRC recurrence^19^.

Despite machine learning’s “black box” reputation, interpreted through the SHAP method, the M1 stage demonstrated the highest contribution, significantly surpassing other features, followed by age and N2 stage. This underscores the ongoing importance of traditional clinical features in survival prediction^28^. CCI ranking as the 4th contributor emphasizes the significant role of comorbidity in predicting survival^29,30^. Additionally, cg20352849, located in the south shelf of the PLCD3 gene, showed a much higher contribution than T4 and T2 stage, indicating its potential value in survival prediction. PLCD3, a phospholipase C family member, which hydrolyze phosphatidylinositol 4,5-diphosphate (PIP2) into diacylglycerol (DAG) and inositol-1,4,5-triphosphate (IP3), initiating Ca2+ release and activating protein kinase C (PKC)^31^. Its role in survival potentially involves the Wnt signaling pathway^32^.

A major strength of our study is its development of predictive signatures for survival in CRC patients, drawing from a large-scale, multi-site, prospective cohort with long-term follow-up and comprehensive clinical variables, including DNAm in blood samples taken over an extended time window and characterization of major tumor subtypes. Additionally, we adapted nested LSOCV and SHAP methods for high generalizability and interoperability. In particular, in contrast to many previous studies, we assessed the predictive value of blood DNAm in models including the best established clinical predictors of prognosis, including TNM stage, enabling assessment of incremental prognostic value beyond those predictors.

However, several limitations should be addressed. Firstly, approximately half of the blood samples were collected within 1 month after surgery, during which DNAm profiles are likely to have been influenced by surgery-related immune and inflammatory factors. This may have compromised the precision of the predictive signature for the entire CRC cohort and prompted us to provide separate analyses for a subset of patients whose blood samples were taken ≥1-month post-surgery. In addition, collection of blood samples at a single point of time prevented longitudinal assessment of changes in DNA methylation as a predictor of colorectal cancer patient survival. Secondly, the limited sample size of CRC patients with blood DNAm before surgery prevented a detailed assessment of the predictive value of presurgery blood DNAm. Thirdly, despite the overall large sample size and multi-site nature of our study, all CRC patients were recruited exclusively from the Rhine-Neckar region in southwest Germany which may limit generalizability. Therefore, external validation in different populations from other countries or with other ethnic composition is necessary. Lastly, some essential prognostic factors, such as lymphovascular invasion (LVI) and presurgery carcinoembryonic antigen (CEA) levels, were not included due to missing values exceeding 50% in this study.

In conclusion, in our multi-site large-scale population-based prospective cohort study, signatures incorporating comprehensive clinical features and blood DNA methylation did not enhance prediction performance compared to algorithms based only on traditional clinical features, including age, sex and TNM stage. This also applied to subsets of patients whose blood samples were taken ≥1-month post-surgery and patients with intermediate TNM stage. M1 stage, age at blood collection, and N2 stage emerged as the top contributors to survival prediction. This rigorously validated finding suggests a limited role for blood DNA methylation in predicting survival in CRC patients. Further research should evaluate the potential use of blood DNA methylation signatures for predicting and monitoring treatment response and CRC recurrence.

## Methods

### Study design and population

Our analysis is based on the DACHS (German name: Darmkrebs: Chancen der Verhütung durch Screening) study, an ongoing population-based case-control study with comprehensive follow-up of CRC cases conducted in the Rhine-Neckar region in southwestern Germany since 2003^33–35^. Briefly, the DACHS study began in 2003 and covered CRC cases from a population of approximately two million people. Eligible participants aged 30 years or older who received a first diagnosis of CRC (ICD-10 codes C18-C20) were recruited from 22 hospitals providing CRC surgery in the study region. Following recruitment by the clinics, personal interviews by trained interviewers were conducted with patients and controls to collect information on lifetime and current exposure to CRC risk and prognostic factors, and blood and tumor samples were collected. Comprehensive follow-up with respect to treatments and overall and disease-specific survival over 10 years after diagnosis was conducted by collecting information from the patients’ treating physicians, record linkage with population registries, and collection of causes of death from public health authorities.

For this analysis, we included 2116 CRC patients who were diagnosed between 2003 and 2010 and from whom DNAm from peripheral whole blood, comprehensive clinical and follow-up data regarding survival outcomes were available (Supplementary Fig. 14). Additionally, we designed two subsets to investigate the potential predictive value of blood DNAm: patients whose blood was collected at least 1 month after surgery (subset 1, N = 1042) and CRC patients with intermediate TNM stages (subset 2, N = 1434).

The DACHS study was approved by the ethics committees of the Medical Faculty of the University of Heidelberg (#310/2001, 06 December 2001), and the Medical Chambers of Baden-Württemberg and Rhineland-Palatinate. Written informed consent was obtained from all participants.

### DNA methylation preprocessing

Peripheral blood samples were collected after the interview and stored at −80 °C. DNA extraction and DNAm assessment based on Infinium MethylationEPIC BeadChip Kit (Illumina, Inc, San Diego, CA, USA), which covers over 850 thousand CpG probes, was conducted according to standard procedures. Details of quality control for samples and CpG probes are displayed in Supplementary Fig. 14^36–38^. Samples that did not meet the quality control criteria, including mismatched sex, low intensity, call-rate <95% on autosome, mean detected *p*-value > 0.01, duplicates, and lacking records of time of blood collection were excluded. Individual CpG probes that did not meet the quality control criteria, including (1) a detection p > 0.01 in any sample; (2) a bead count <3 in at least 5% of samples; (3) not CpG sites; (4) single nucleotide polymorphisms (SNPs)^39^; (5) align to multiple locations^40,41^; and (6) targeting sex chromosomes, were filtered out. Noob correction and beta-mixture quantile (BMIQ)^42^ were applied to normalize beta values (ranging from 0 to 1, i.e., from completely unmethylated to completely methylated) and batch correction were applied before machine learning analysis.

### Machine learning procedure

We constructed the prognostic signatures for overall survival, as well as CRC-specific survival. Survival time was defined as the period from the date of blood collection to the date of death or cancer-related death, or the last follow-up. Living participants were censored at the end of each follow-up period. A 5-fold nLSOCV scheme (Fig. 1), in which XGBoost was applied to develop predictive models for survival of CRC patients, was used separately for the total CRC cohort and the two subsets. CRC patients were split into five groups of approximately equal size according to their hospitals and institutions (Supplementary Table 14). Each group, in turn, served as the test set, with the remaining four subsets being the training set. In the outer-loop, a two-step filtering process was employed for selecting features. Firstly, we conducted Cox regression analysis on CpG sites that have passed quality control measures, aiming to select CpG sites associated with overall survival. This Cox regression adjusted for age at blood collection, sex, TNM stage, smoking status and alcohol consumption. Subsequently, we identified and selected the 5000 CpG sites with the lowest Benjamini-Hochberg adjusted *p* values (BH-adjusted *p*-values) for the corresponding Wald test. The second step aimed to selected predictive features with the elastic net (EN) approach^43^. The predictive features were selected based on comprehensive clinical features and all 5,000 survival-related CpGs selected in the previous step. XGBoost, which consistently achieves state-of-the-art performance in model prediction^44^, was then applied with selected predictive features. Both EN and XGBoost underwent another 5-fold cross-validation for hyperparameter tuning with grid search, called inner-loop. Performance evaluation involved discrimination and calibration indicators, aggregating results from each test set in the outer loops.

We developed 4 predictive models with the nLSOCV scheme using specific feature groups: (1) *Model 1*: traditional clinical features including age at blood collection, sex and separately stages of tumor size and invasion (T), lymph nodes involvement (N), and distant metastasis (M) ; (2) *Model 2*: comprehensive clinical features, incorporating additional features including age at diagnosis, tumor location, family history of CRC, resection edge, tumor differential grading, histological type, CCI^29^, body mass index (BMI), smoking status, pack-years of cigarette consumption, alcohol consumption, average lifetime physical activity, diet quality score^45^, occupational position, employment situation, MSI, KRAS, BRAF, CIMP^46^, neo-chemotherapy, neo-radiotherapy, adjuvant-chemotherapy, adjuvant-radiotherapy, relapse, metastasis, and surgery, adjuvant-chemotherapy and adjuvant-radiotherapy after relapse and metastasis (Supplementary Table 15); (3) *Model 3*: processed blood DNAm; and (4) *Model 4*: features after two-step filtering in each outer-loop, incorporating blood CpGs from Model 3 with clinical features from Model 2. Missing data of clinical features were imputed with the missforest method^47^. MissForest is a non-parametric, iterative imputation technique that utilizes the Random Forest algorithm. This method inherently implements a multiple imputation approach by averaging across numerous unpruned classifications or regression trees, enabling the handling of multivariate data, including both continuous and categorical variables, simultaneously.

### Model evaluation and visualization

We evaluated the model’s performance at several specific follow-up times post-surgery, including at 1, 3, 5, and 10 years, to identify potential drift over time^48^. The model’s discriminatory ability was assessed using the Kaplan-Meier (KM) curve for signatures dichotomized by the median value and time-dependent ROC curves, along with calculating the AUROC. The ROC curve illustrates the trade-off between the true positive rate (TPR) and false positive rate (FPR) across various decision thresholds at specific times. A higher AUROC value indicates better predictive accuracy of the model. To align our findings with studies that employ the C-index, which captures the model’s capability to differentiate among predictions regarding risk, event occurrence, and time in a single metric but may reflect overconfidence in model discrimination^49^, we also measured the C-index within a 10-year time window. The time-dependent AUROCs and C-indexes in each outer-loop of different models were compared. The calibration performance was examined using the time-dependent Brier score and the integrated Brier score (IBS). The Brier score, which ranges from 0 to 1, measures the average squared difference between observed survival status at specific times and the predicted probabilities of survival, with a lower score indicating more accurate predictions. The IBS evaluates the overall accuracy of survival predictions over a specified time window of 10 years, reflecting the squared differences between the observed and predicted survival curves. Calibration curves were plotted; ideally, a perfectly calibrated model would exhibit a curve that closely aligns with the 45-degree diagonal line. The strict setting of nLSOCV ensured that the test set remained unseen during training, limiting predictive models’ performance but enhancing overall.

We used SHAP analysis to identify features with critical contribution in predicting risk of death in XGBoost models^50^. Higher SHAP values correspond to a higher risk of death. A summary plot was used to visualize the features’ contribution to predictive model.

### Statistical analysis

Descriptive statistics were used to characterize the distribution of baseline variables for the total CRC cohort, subset 1 and subset 2. Categorical covariates were summarized using absolute and relative frequencies, while median and interquartile range were presented for continuous variables.

All statistical analyses were performed using R language program (version 4.1.2) with R Studio (version 1.4.1717; Boston, USA). We utilized several R packages for different aspects of the analysis: ChAMP^38^, limma^36^, and minfi^37^ for DNAm preprocessing; missForest^47^ for data imputation; survival and survminer^51^ for Cox regression; grpreg^43^ for developing the EN model; xgboost and survXgboost for developing the XGBoost model^44^; mlr3, mlr3proba, and mlr3extralearners for hyperparameter tuning^52^; pec^53^, Riskregression^48^, and compareC^54^ for model evaluations; and SHAPforxgboost^50^ for SHAP evaluation. Statistically significant *p*-values were defined as those with two-tailed *p* < 0.05, after BH correction for multiple comparisons where necessary.

## Supplementary information

Supplementary Information

## Data Availability

The datasets generated and analyzed during the current study are not publicly available due ethical and legal restrictions but are available from the corresponding author on reasonable request.

## Code availability

The code is available from the corresponding author upon reasonable request.
