# Supplementary material for: Developing survival prediction models in colorectal cancer using epigenome-wide DNA methylation data from whole blood
Source: NPJ Precis Oncol. 2024 Sep 6;8:191. doi: 10.1038/s41698-024-00689-5 (PMC11377733; doi:10.1038/s41698-024-00689-5)
Supplement: Supplementary file 1 — Supplementary Information [file 41698_2024_689_MOESM1_ESM.pdf]

Supplementary Information

Contents

Supplementary Figure 1. Distribution of blood sampling times. 3

Supplementary Figure 2. Kaplan-Meier curves of four predictive models in the total CRC cohort. 4

Supplementary Figure 3. Time-dependent ROC curves of four predictive models in the total CRC cohort. 5

Supplementary Figure 4. Time-dependent calibration curves of four predictive models in the total CRC cohort. 6

Supplementary Figure 5. Kaplan-Meier curves of four predictive models in subset 1. 7

Supplementary Figure 6. Time-dependent ROC curves of four predictive models in subset 1. 8

Supplementary Figure 7. Time-dependent calibration curves of four predictive models in subset 1. 9

Supplementary Figure 8. Kaplan-Meier curves of four predictive models in subset 2. 10

Supplementary Figure 9. Time-dependent ROC curves of four predictive models in subset2. 11

Supplementary Figure 10. Time-dependent calibration curves of four predictive models in subset 2. 12

Supplementary Figure 11. Kaplan-Meier curves of four predictive models for CSS in the total CRC cohort. 13

Supplementary Figure 12. Kaplan-Meier curves of four predictive models for CSS in subset 1. 14

Supplementary Figure 13. Kaplan-Meier curves of four predictive models for CSS in subset 2. 16

Supplementary Figure 14. Sample selection criteria and DNA methylation preprocessing. 17

Supplementary Table 1. Comparison of 10-year C-index and time-dependent AUROC between different predictive models in each outer-loop of the total CRC cohort (N= 2116). 18

Supplementary Table 2. Performance and calibration of predictive models for overall survival in each outer-loop of the total CRC cohort (N= 2116). 20

Supplementary Table 3. Comparison of 10-year C-index and time-dependent AUROC between different predictive models in each outer-loop of subset 1 (N= 1042). 21

Supplementary Table 4. Performance and calibration of predictive models for overall survival in each outer-loop of subset 1 (N= 1042). 22

Supplementary Table 5. Comparison of 10-year C-index and time-dependent AUROC between different predictive models in each outer-loop of subset 2 (N= 1434). 23

Supplementary Table 6. Performance and calibration of predictive models for overall survival in each outer-loop of subset 2 (N= 1434). 24

Supplementary Table 7. Performance and calibration of predictive models for CSS of in the total CRC cohort and two subsets. 25

Supplementary Table 8. Comparison of 10-year C-index and time-dependent AUROC between different predictive models for CSS in each outer-loop of the total CRC cohort (N= 2116). 26

Supplementary Table 9. Performance and calibration of predictive models for CSS in each outer-loop of the total CRC cohort (N= 2116). 28

Supplementary Table 10. Comparison of 10-year C-index and time-dependent AUROC between different predictive models for CSS in each outer-loop of subset 1 (N= 1042). 29

Supplementary Table 11. Performance and calibration of predictive models for CSS in each outer-loop of subset 1 (N= 1042). 30

Supplementary Table 12. Comparison of 10-year C-index and time-dependent AUROC between different predictive models for CSS in each outer-loop of subset 2 (N= 1434). 31

Supplementary Table 13. Performance and calibration of predictive models for CSS in each outer-loop of subset 2 (N= 1434). 32

Supplementary Table 14. Number of patients in leave-sites-out 5-fold nested cross-validation 33

Supplementary Table 15. Characteristics of comprehensive clinical features. 34
TRIPODAI checklist 37

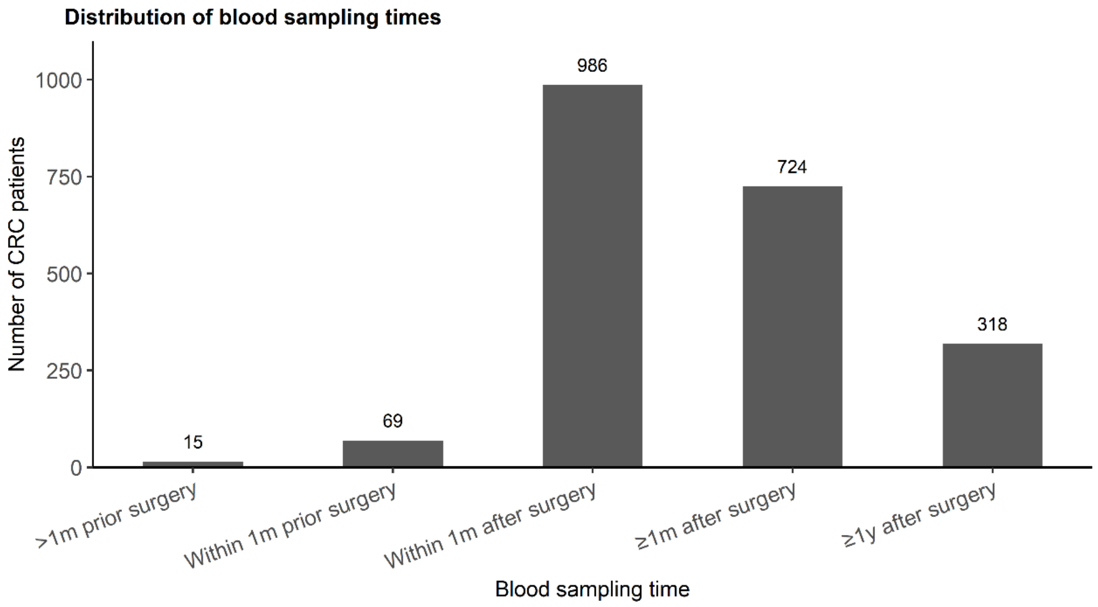

Supplementary Figure 1. Distribution of blood sampling times.

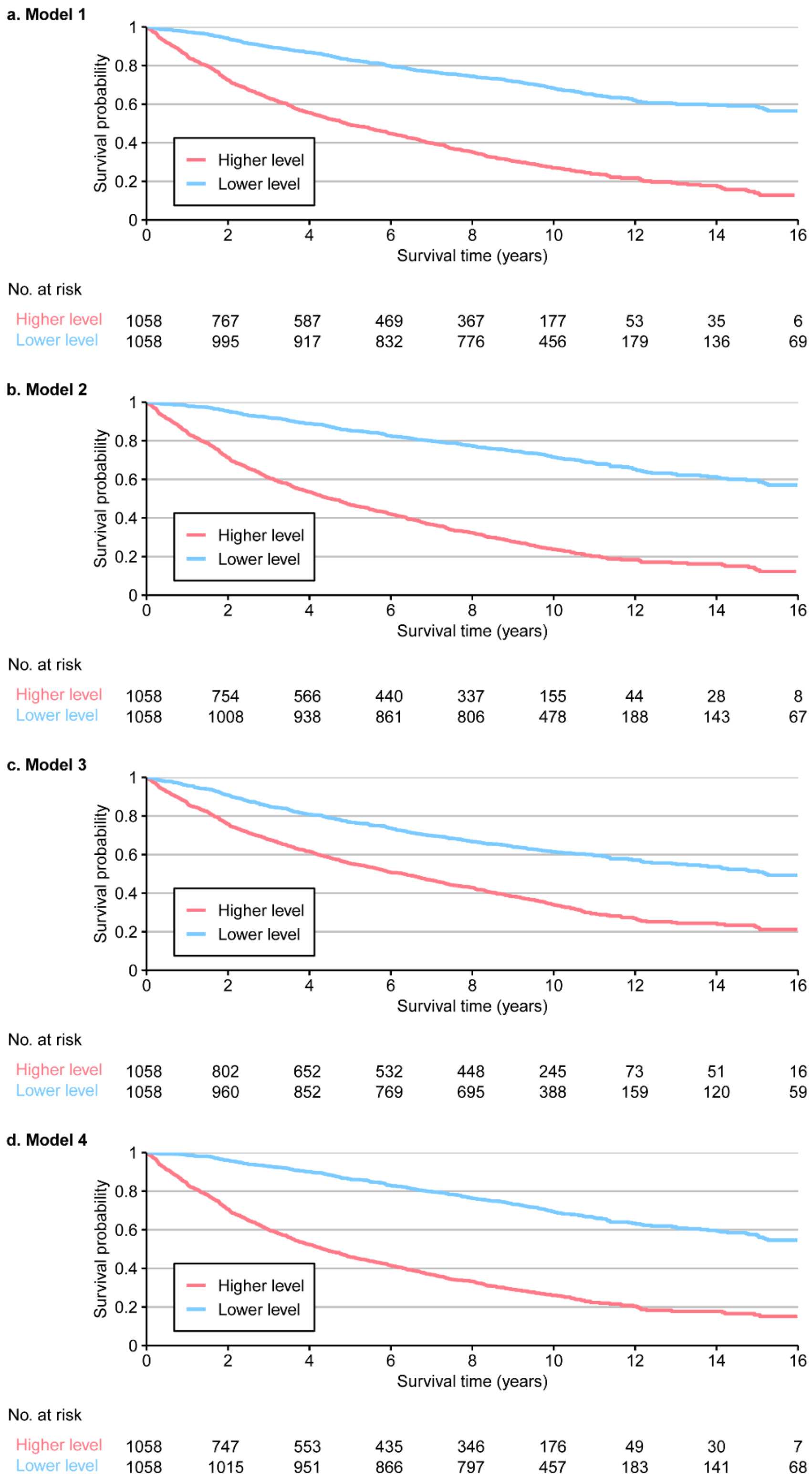

Supplementary Figure 2. Kaplan-Meier curves of four predictive models in the total CRC cohort.

Each model was dichotomized by the median value. The Kaplan-Meier curves illustrate the overall survival probability over time for higher and lower levels of predicted risk for (a) Model 1 based on traditional clinical features, (b) Model 2 based on comprehensive clinical features, (c) Model 3 based on blood DNA methylation, (d) Model 4 combined both clinical features and blood DNA methylation. Abbreviations: CRC, colorectal cancer; ROC, receiver operating characteristic curve.

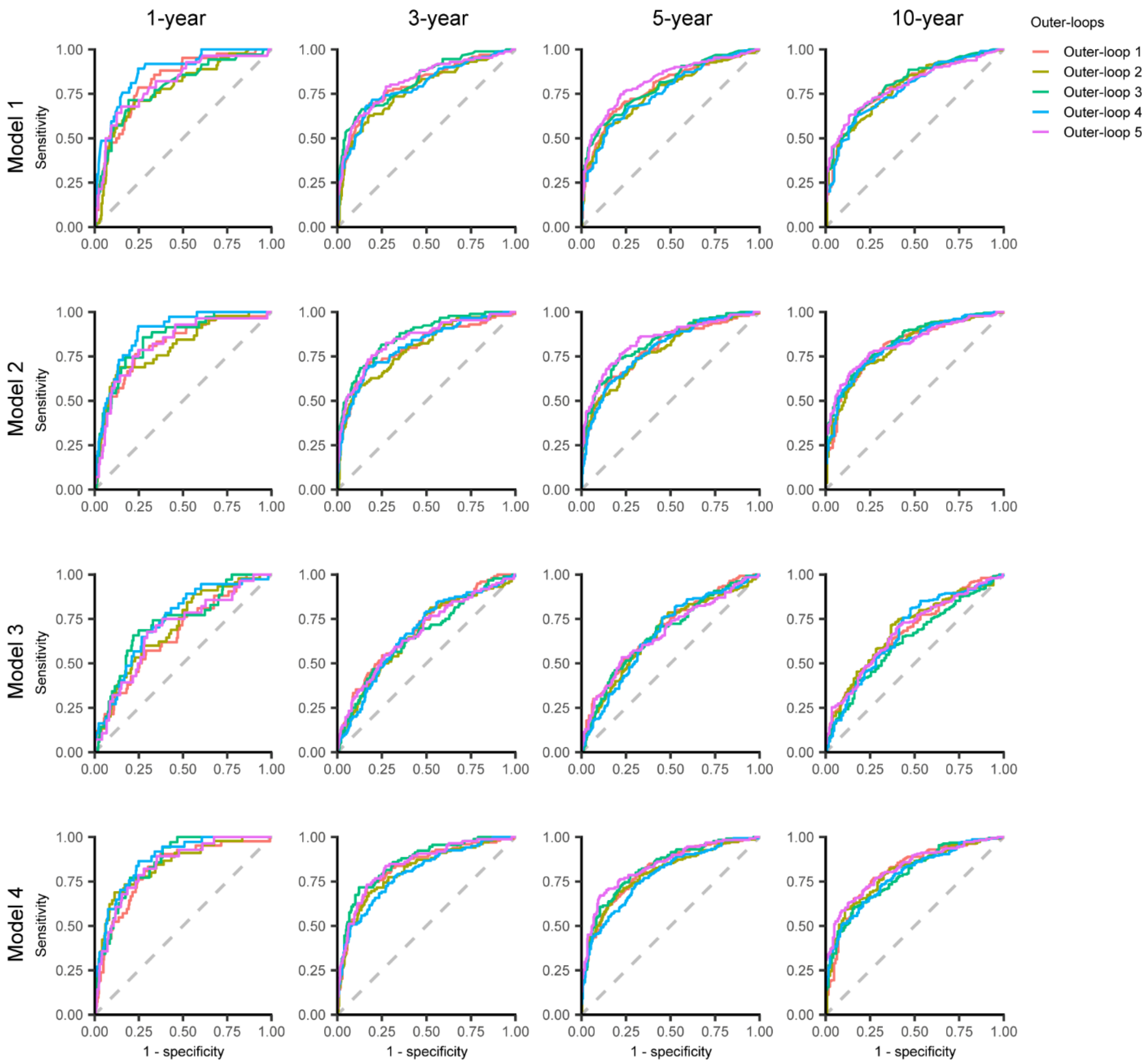

Supplementary Figure 3. Time-dependent ROC curves of four predictive models in the total CRC cohort.

Model 1 based on traditional clinical features, Model 2 based on comprehensive clinical features, Model 3 based on blood DNA methylation, Model 4 combined both clinical features and blood DNA methylation.

Abbreviations: CRC, colorectal cancer; ROC, receiver operating characteristic curve.

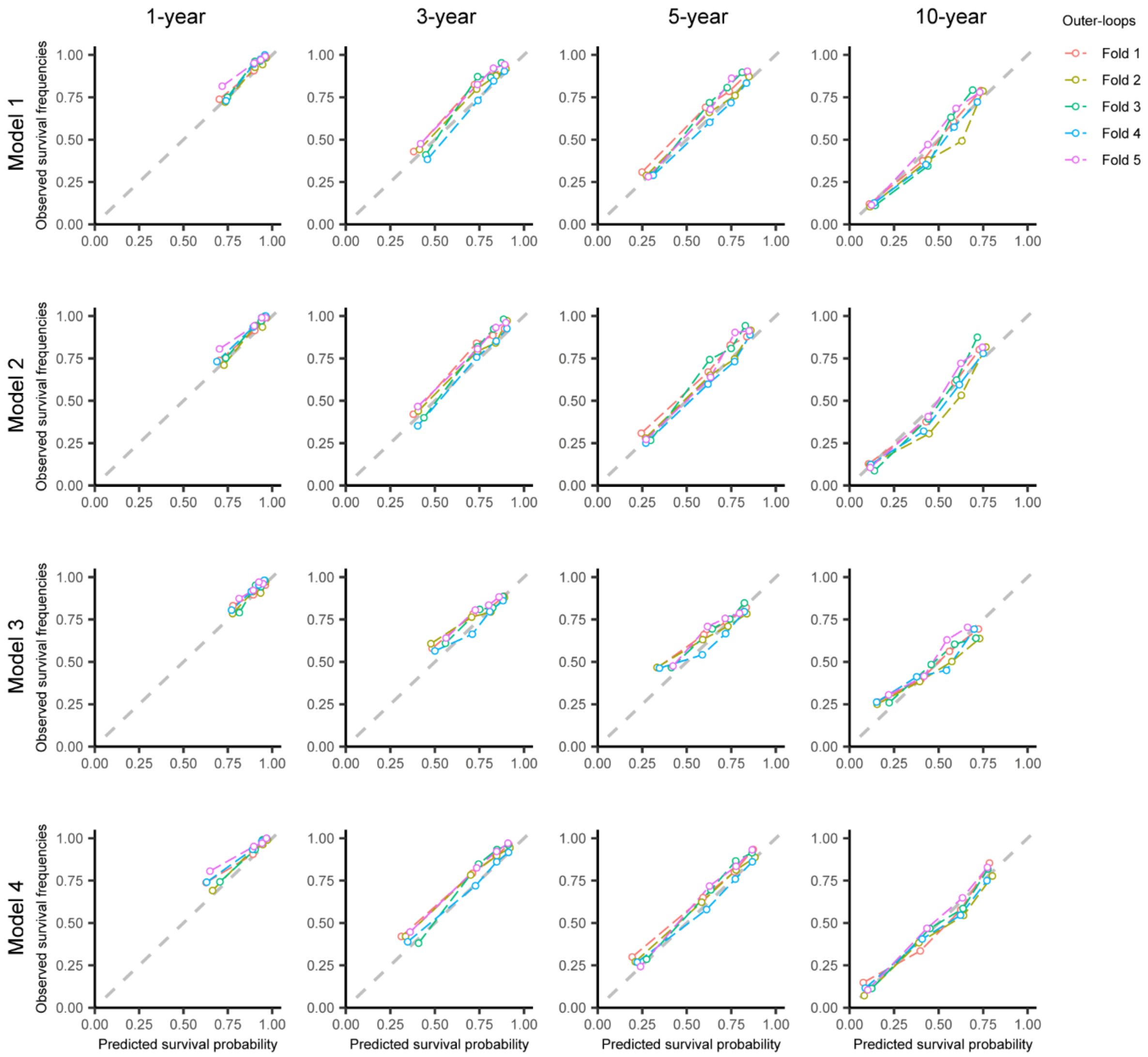

Supplementary Figure 4. Time-dependent calibration curves of four predictive models in the total CRC cohort.

Model 1 based on traditional clinical features, Model 2 based on comprehensive clinical features, Model 3 based on blood DNA methylation, Model 4 combined both clinical features and blood DNA methylation.

Abbreviations: CRC, colorectal cancer.

a. Model 1

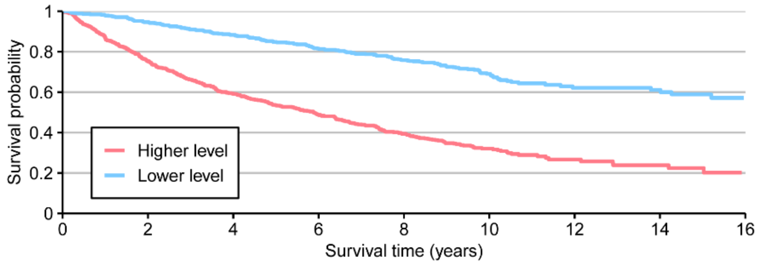

No. at risk

	0	2	4	6	8	10	12	14	16
Higher level	521	392	307	252	204	82	29	17	5
Lower level	521	493	459	416	386	166	80	56	19

b. Model 2

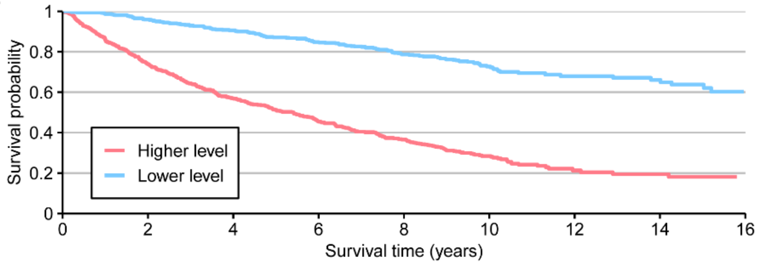

No. at risk

	0	2	4	6	8	10	12	14	16
Higher level	521	385	296	236	190	76	24	15	6
Lower level	521	500	470	432	400	172	85	58	18

c. Model 3

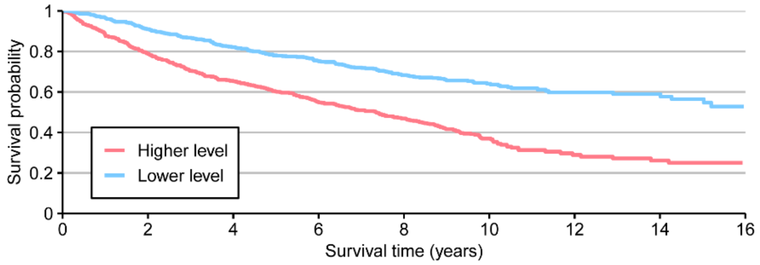

No. at risk

	0	2	4	6	8	10	12	14	16
Higher level	521	411	340	284	241	95	35	25	5
Lower level	521	474	426	384	349	153	74	48	19

d. Model 4

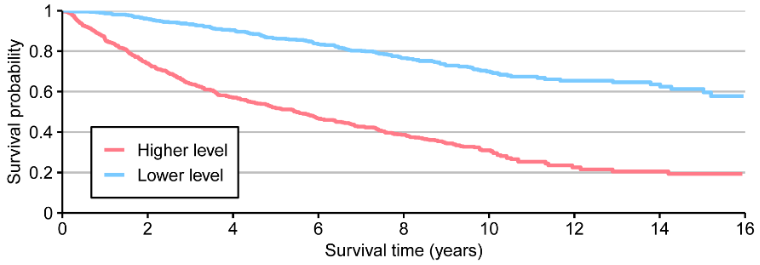

No. at risk

	0	2	4	6	8	10	12	14	16
Higher level	521	385	296	242	199	70	23	17	5
Lower level	521	500	470	426	391	178	86	56	19

Supplementary Figure 5. Kaplan-Meier curves of four predictive models in subset 1.

Subset 1 includes CRC patients with blood samples collected ≥1-month after surgery. Each model was dichotomized by the median value. The Kaplan-Meier curves illustrate the overall survival probability over time for higher and lower levels of predicted risk for (a) Model 1 based on traditional clinical features, (b) Model 2 based on comprehensive clinical features, (c) Model 3 based on blood DNA methylation, (d) Model 4 combined both clinical features and blood DNA methylation. Abbreviations: CRC, colorectal cancer; ROC, receiver operating characteristic curve.

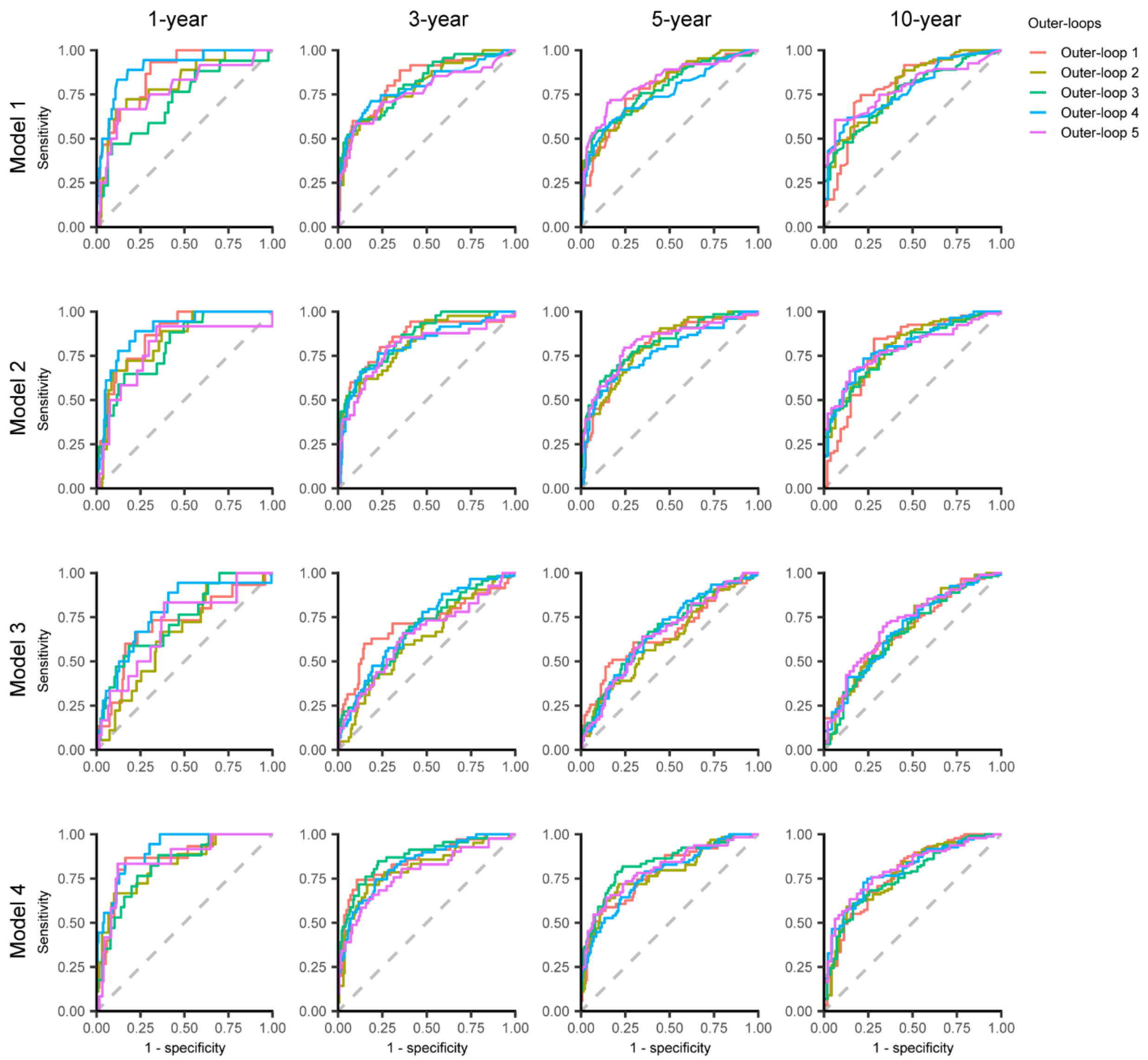

Supplementary Figure 6. Time-dependent ROC curves of four predictive models in subset 1.

Subset 1 includes CRC patients with blood samples collected ≥1-month after surgery. Model 1 based on traditional clinical features, Model 2 based on comprehensive clinical features, Model 3 based on blood DNA methylation, Model 4 combined both clinical features and blood DNA methylation. Abbreviations: CRC, colorectal cancer; ROC, receiver operating characteristic curve.

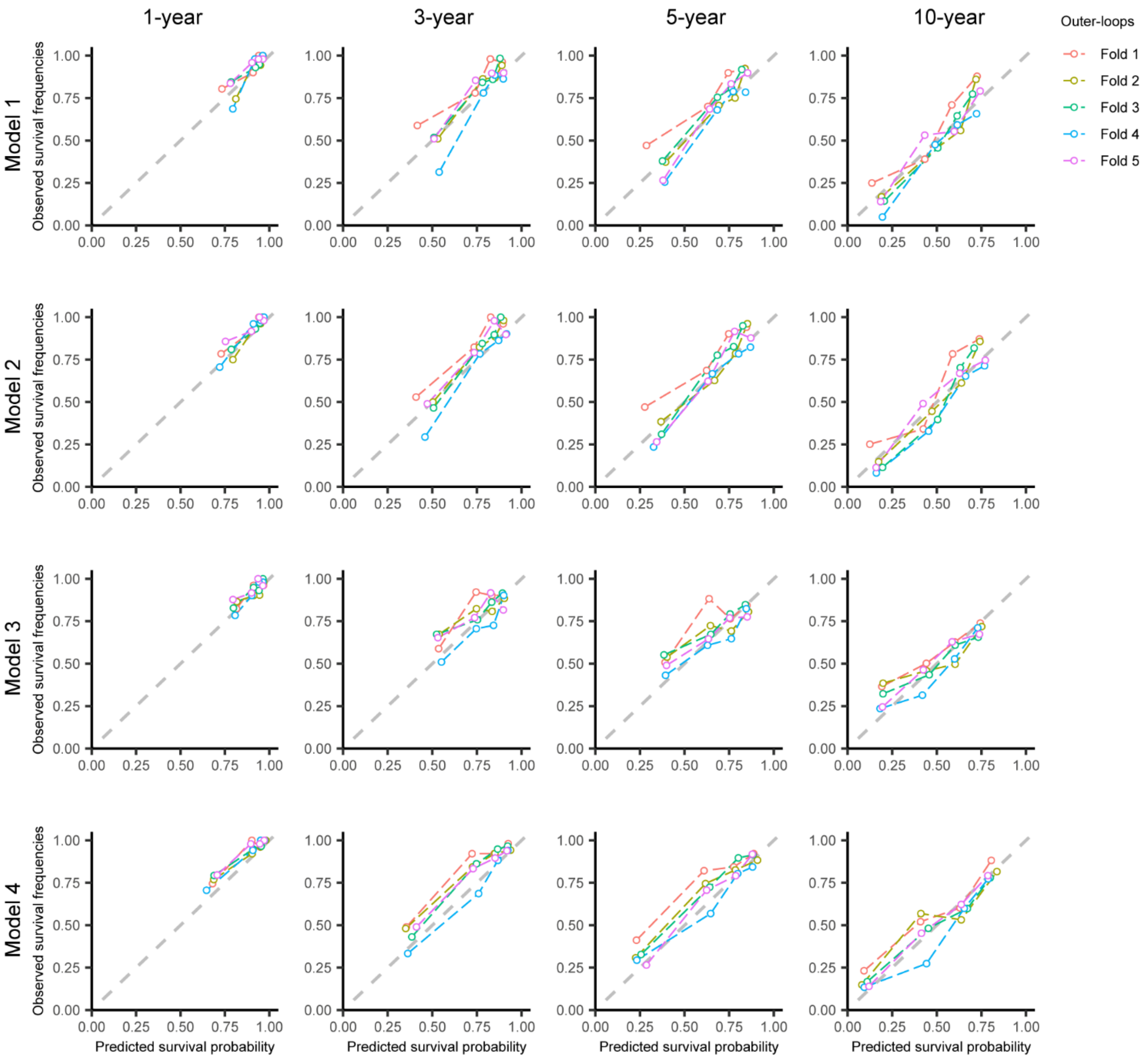

Supplementary Figure 7. Time-dependent calibration curves of four predictive models in subset 1.

Subset 1 includes patients with blood samples collected ≥1-month after surgery. Model 1 based on traditional clinical features, Model 2 based on comprehensive clinical features, Model 3 based on blood DNA methylation, Model 4 combined both clinical features and blood DNA methylation.
Abbreviations: CRC, colorectal cancer.

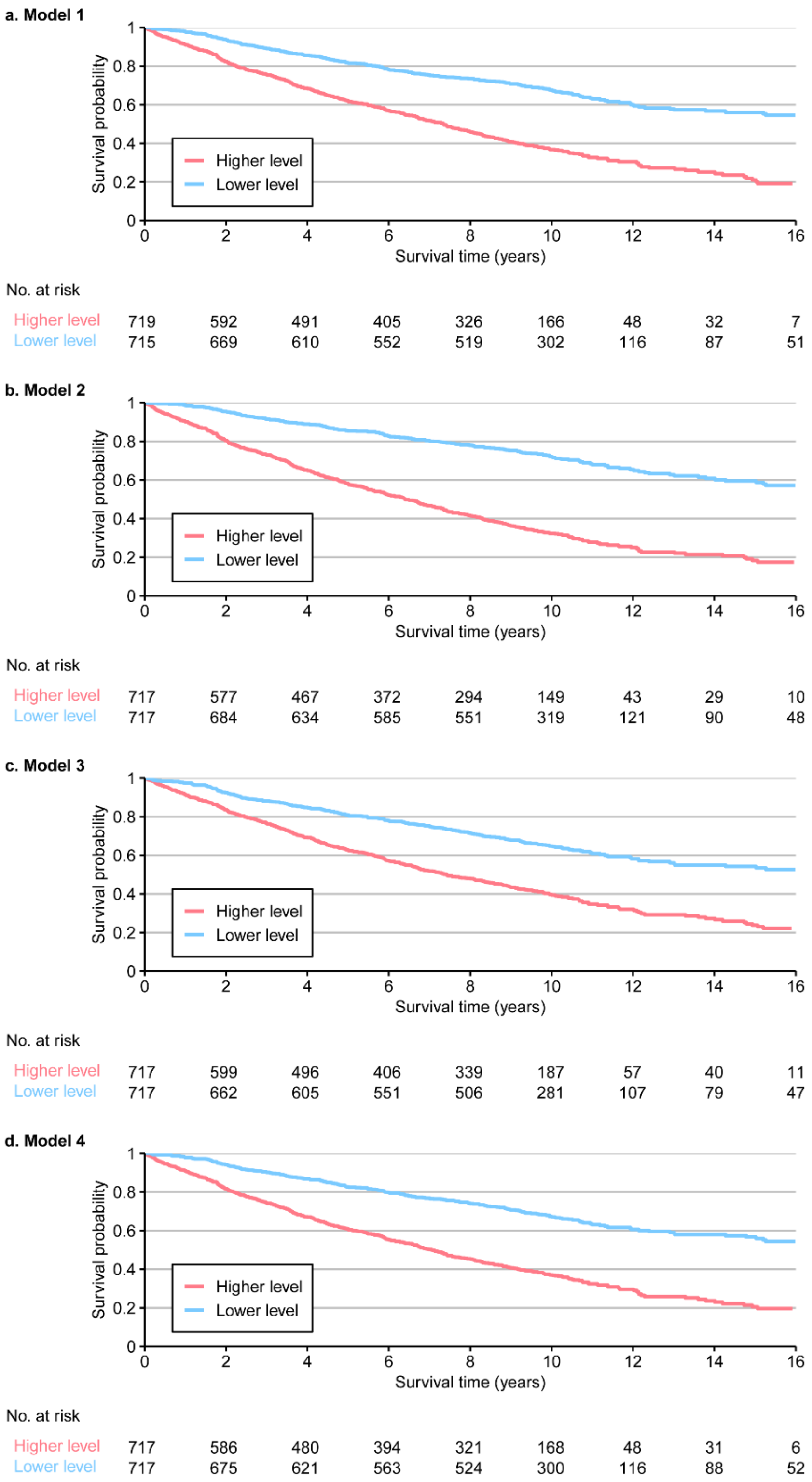

Supplementary Figure 8. Kaplan-Meier curves of four predictive models in subset 2.

Subset 2 includes CRC patients with TNM stage II-III. Each model was dichotomized by the median value. The Kaplan-Meier curves illustrate the overall survival probability over time for higher and lower levels of predicted risk for (a) Model 1 based on traditional clinical features, (b) Model 2 based on comprehensive clinical features, (c) Model 3 based on blood DNA methylation, (d) Model 4 combined both clinical features and blood DNA methylation.
Abbreviations: CRC, colorectal cancer; ROC, receiver operating characteristic curve.

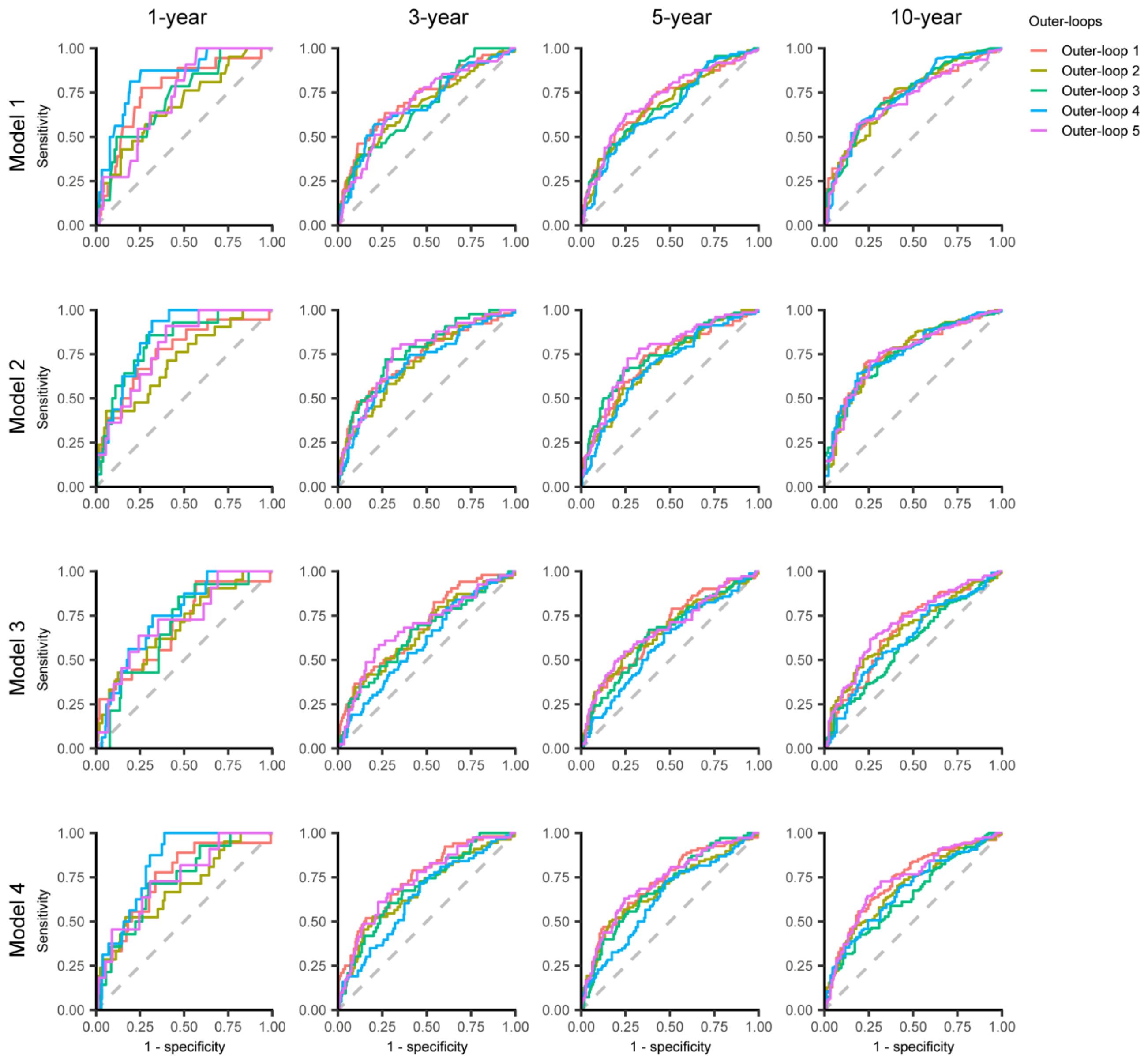

Supplementary Figure 9. Time-dependent ROC curves of four predictive models in subset2.

Subset 2 includes CRC patients with TNM stage II-III. Model 1 based on traditional clinical features, Model 2 based on comprehensive clinical features, Model 3 based on blood DNA methylation, Model 4 combined both clinical features and blood DNA methylation.
Abbreviations: CRC, colorectal cancer; ROC, receiver operating characteristic curve.

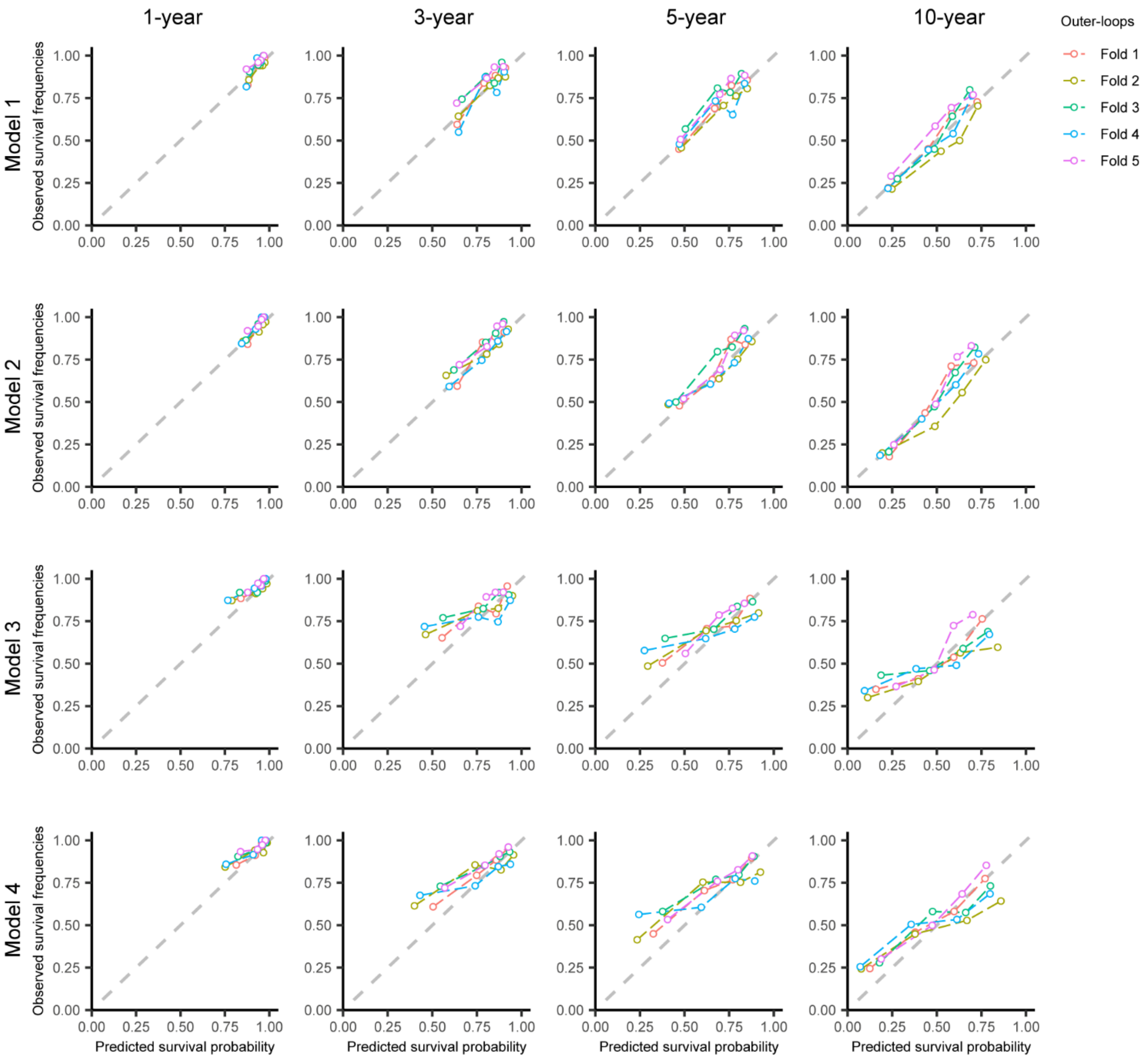

Supplementary Figure 10. Time-dependent calibration curves of four predictive models in subset 2.

Subset 2 includes patients with TNM stage II-III. Model 1 based on traditional clinical features, Model 2 based on comprehensive clinical features, Model 3 based on blood DNA methylation, Model 4 combined both clinical features and blood DNA methylation.
Abbreviations: CRC, colorectal cancer.

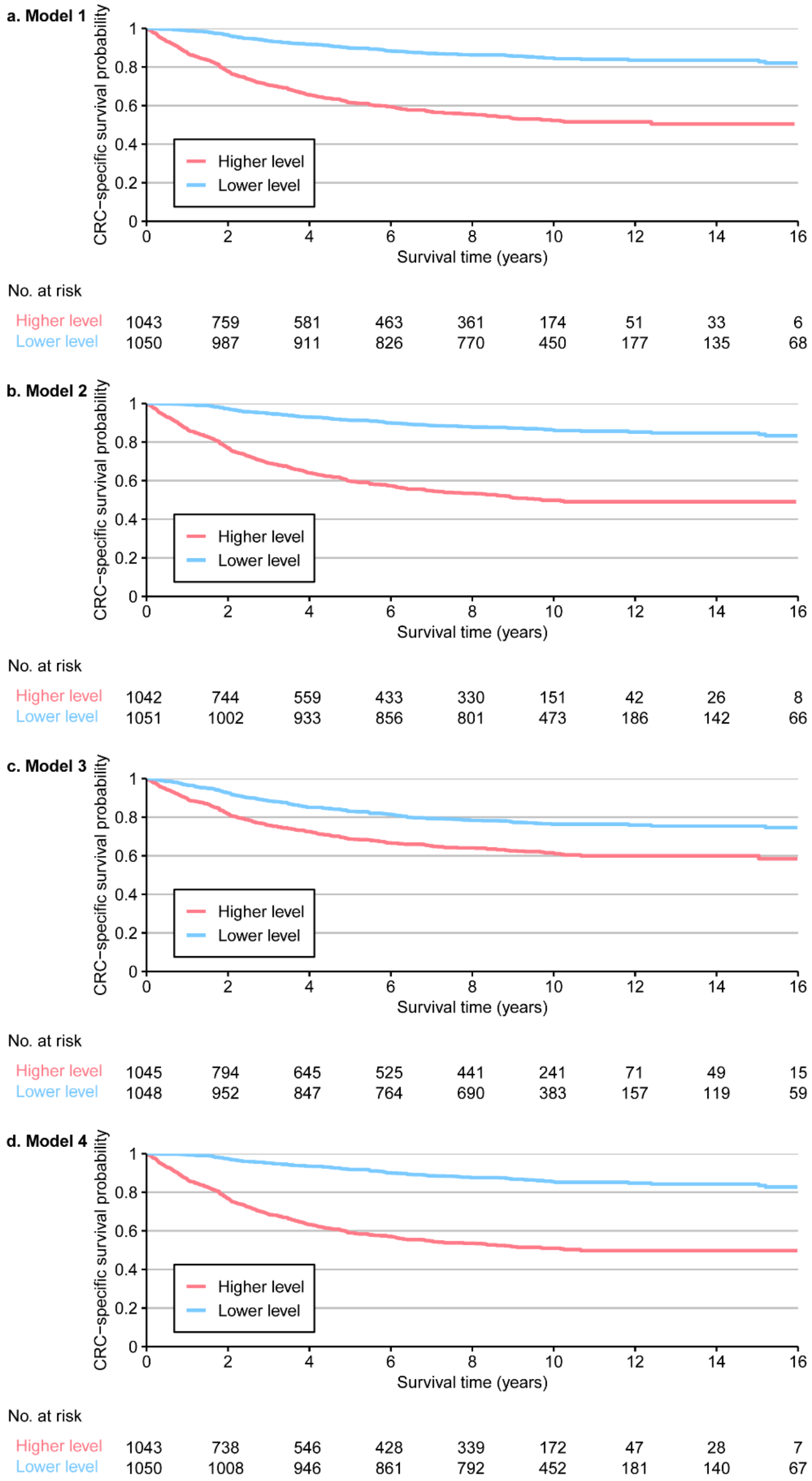

Supplementary Figure 11. Kaplan-Meier curves of four predictive models for CSS in the total CRC cohort.

Each model was dichotomized by the median value. The Kaplan-Meier curves illustrate the CRC-specific survival probability over time for higher and lower levels of predicted risk for (a) Model 1 based on traditional clinical features, (b) Model 2 based on comprehensive clinical features, (c) Model 3 based on blood DNA methylation, (d) Model 4 combined both clinical features and blood DNA methylation.

Abbreviations: CSS, CRC-specific survival; CRC, colorectal cancer; ROC, receiver operating

characteristic curve.

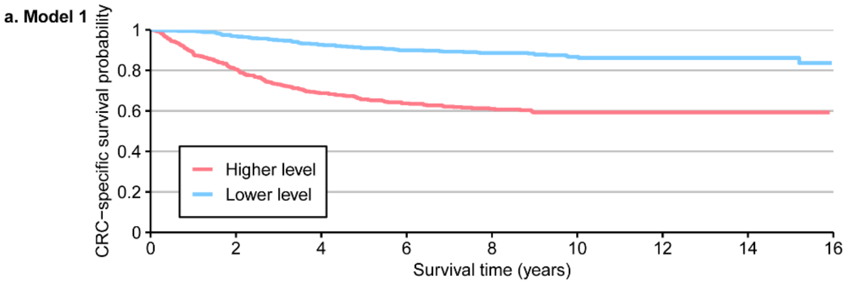

No. at risk

	0	2	4	6	8	10	12	14	16
Higher level	515	390	305	250	202	81	29	17	5
Lower level	517	489	456	413	383	163	80	56	19

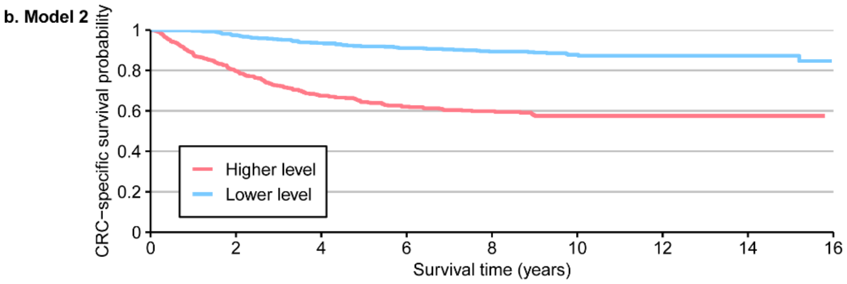

No. at risk

	0	2	4	6	8	10	12	14	16
Higher level	514	382	293	233	187	74	24	15	6
Lower level	518	497	468	430	398	170	85	58	18

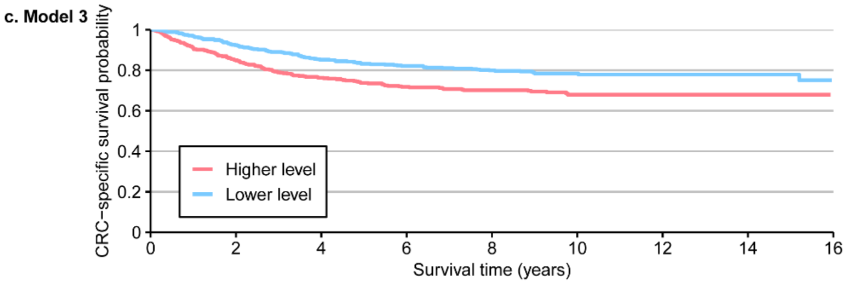

No. at risk

	0	2	4	6	8	10	12	14	16
Higher level	517	409	338	282	239	94	35	25	5
Lower level	515	470	423	381	346	150	74	48	19

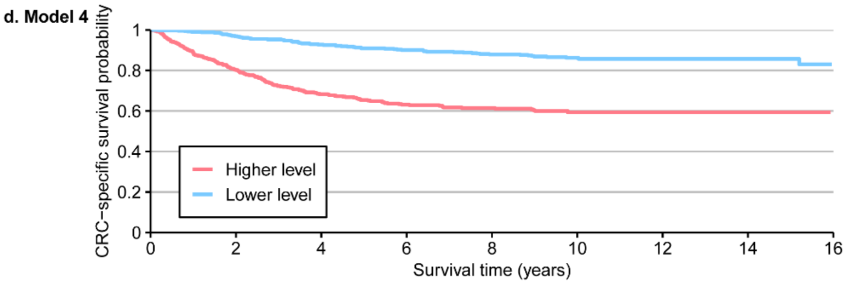

No. at risk

	0	2	4	6	8	10	12	14	16
Higher level	516	383	294	240	197	69	23	17	5
Lower level	516	496	467	423	388	175	86	56	19

Supplementary Figure 12. Kaplan-Meier curves of four predictive models for CSS in subset 1.

Subset 1 includes patients with blood samples collected ≥1-month after surgery. Each model was dichotomized by the median value. The Kaplan-Meier curves illustrate the CRC-specific survival probability over time for higher and lower levels of predicted risk for (a) Model 1 based on traditional clinical features, (b) Model 2 based on comprehensive clinical features, (c) Model 3 based on blood DNA methylation, (d) Model 4 combined both clinical features and blood DNA methylation.

Abbreviations: CSS, CRC-specific survival; CRC, colorectal cancer; ROC, receiver operating characteristic curve.

a. Model 1

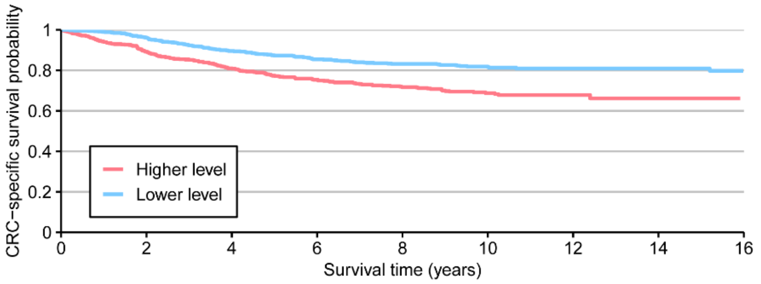

No. at risk

	0	2	4	6	8	10	12	14	16
Higher level	709	586	486	400	321	164	47	31	7
Lower level	707	661	604	546	513	296	114	86	50

b. Model 2

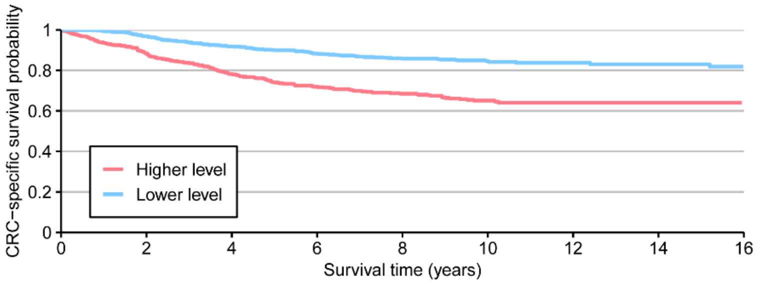

No. at risk

	0	2	4	6	8	10	12	14	16
Higher level	704	568	459	364	286	144	41	27	9
Lower level	712	679	631	582	548	316	120	90	48

c. Model 3

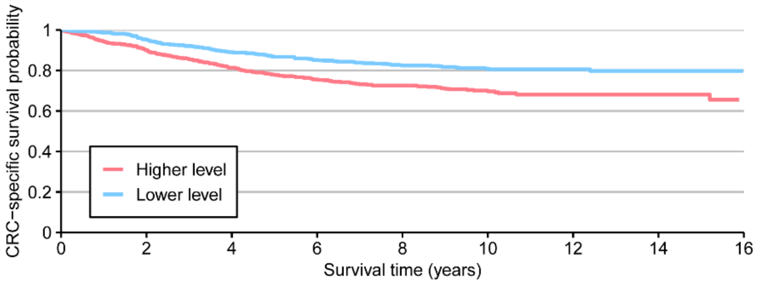

No. at risk

	0	2	4	6	8	10	12	14	16
Higher level	706	591	489	399	332	183	55	38	10
Lower level	710	656	601	547	502	277	106	79	47

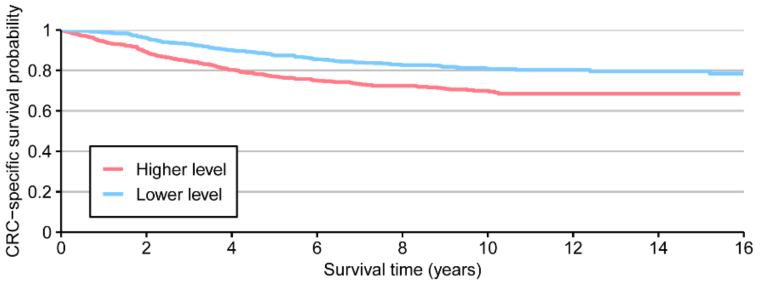

No. at risk

	0	2	4	6	8	10	12	14	16
Higher level	705	578	473	387	314	164	46	29	5
Lower level	711	669	617	559	520	296	115	88	52

.

Supplementary Figure 13. Kaplan-Meier curves of four predictive models for CSS in subset 2.

Subset 2 includes patients with TNM stage II-III. Each model was dichotomized by the median value. The Kaplan-Meier curves illustrate the CRC-specific survival probability over time for higher and lower levels of predicted risk for (a) Model 1 based on traditional clinical features, (b) Model 2 based on comprehensive clinical features, (c) Model 3 based on blood DNA methylation, (d) Model 4 combined both clinical features and blood DNA methylation.
Abbreviations: CSS, CRC-specific survival; CRC, colorectal cancer; ROC, receiver operating characteristic curve.

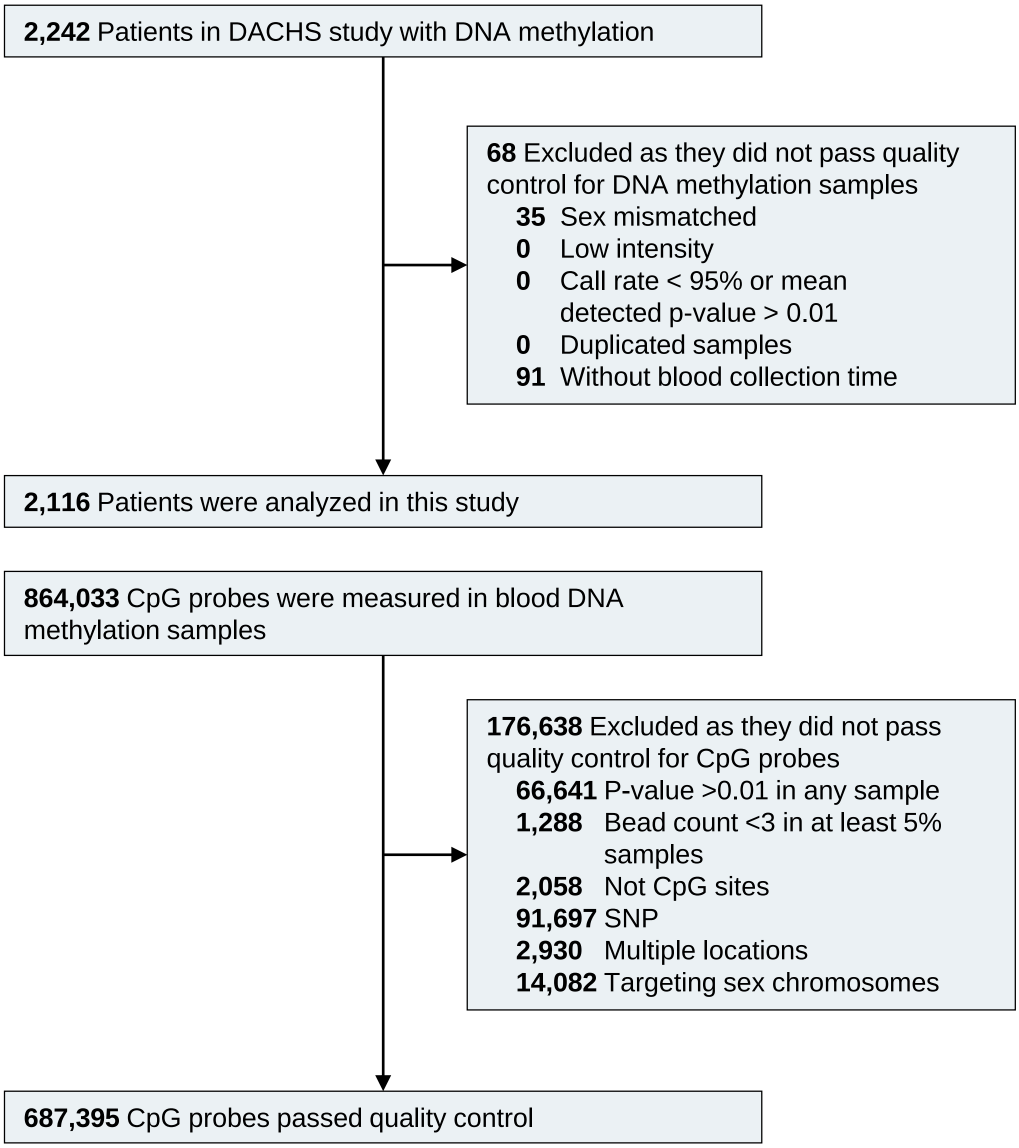

Supplementary Figure 14. Sample selection criteria and DNA methylation preprocessing.

The sum of excluded CpG number is not equal to 176,638 because some are duplicated among different exclusion criteria.
Abbreviations: CRC, colorectal cancer; DNAm, DNA methylation; SNP, single nucleotide polymorphisms.

Supplementary Table 1. Comparison of 10-year C-index and time-dependent AUROC between different predictive models in each outer-loop of the total CRC cohort (N= 2116).

**Models**	**Outer-loop 1**		**Outer-loop 2**		**Outer-loop 3**		**Outer-loop 4**		**Outer-loop 5**	
	**ΔValue (SE)**	***p***	**ΔValue (SE)**	***p***	**ΔValue (SE)**	***p***	**ΔValue (SE)**	***p***	**ΔValue (SE)**	***p***
Model 2 vs. Model 1										
C-index (10y)	0.0043 (0.0080)	0.59	0.0173 (0.0078)	0.03	**0.0233 (0.0078)**	**<0.01**	**0.0217 (0.0072)**	**<0.01**	**0.0221 (0.0073)**	**<0.01**
1y AUROC	-0.0025 (0.0148)	0.87	0.0239 (0.0160)	0.14	**0.0530 (0.0186)**	**<0.01**	0.0057 (0.0099)	0.56	0.0069 (0.0157)	0.66
3y AUROC	0.0023 (0.0114)	0.84	0.0225 (0.0136)	0.1	**0.0380 (0.0125)**	**<0.01**	0.0193 (0.0114)	0.09	**0.0230 (0.0102)**	**0.02**
5y AUROC	0.0057 (0.0114)	0.62	0.0219 (0.0116)	0.06	**0.0327 (0.0115)**	**<0.01**	**0.0303 (0.0107)**	**<0.01**	0.0172 (0.0094)	0.07
10y AUROC	0.0140 (0.0122)	0.25	0.0183 (0.0112)	0.1	**0.0228 (0.0111)**	**0.04**	**0.0356 (0.0107)**	**<0.01**	**0.0260 (0.0103)**	**0.01**
Model 3 vs. Model 1										
C-index (10y)	**-0.0887 (0.0207)**	**<0.01**	**-0.0811 (0.0207)**	**<0.01**	**-0.1175 (0.0242)**	**<0.01**	**-0.0825 (0.0206)**	**<0.01**	**-0.0988 (0.0233)**	**<0.01**
1y AUROC	**-0.1507 (0.0472)**	**<0.01**	-0.0706 (0.0502)	0.16	-0.0624 (0.0642)	0.33	**-0.1374 (0.0466)**	**<0.01**	-0.1189 (0.0650)	0.07
3y AUROC	**-0.1081 (0.0362)**	**<0.01**	**-0.1083 (0.0388)**	**<0.01**	**-0.1663 (0.0425)**	**<0.01**	**-0.1196 (0.0323)**	**<0.01**	**-0.1449 (0.0402)**	**<0.01**
5y AUROC	**-0.0974 (0.0315)**	**<0.01**	**-0.0964 (0.0317)**	**<0.01**	**-0.1247 (0.0363)**	**<0.01**	**-0.0961 (0.0294)**	**<0.01**	**-0.1518 (0.0336)**	**<0.01**
10y AUROC	**-0.1173 (0.0318)**	**<0.01**	**-0.0938 (0.0299)**	**<0.01**	**-0.1764 (0.0342)**	**<0.01**	**-0.0994 (0.0335)**	**<0.01**	**-0.1131 (0.0327)**	**<0.01**
Model 4 vs. Model 1										
C-index (10y)	0.0152 (0.0105)	0.15	**0.0255 (0.0108)**	**0.02**	0.0086 (0.0131)	0.51	0.0100 (0.0112)	0.37	**0.0234 (0.0116)**	**0.04**
1y AUROC	-0.0025 (0.0217)	0.91	**0.0654 (0.0185)**	**<0.01**	**0.0657 (0.0279)**	**0.02**	-0.0015 (0.0174)	0.93	0.0271 (0.0233)	0.24
3y AUROC	0.0282 (0.0162)	0.08	**0.0375 (0.0189)**	**<0.05**	0.0362 (0.0203)	0.08	0.0079 (0.0175)	0.65	0.0261 (0.0172)	0.13
5y AUROC	0.0257 (0.0152)	0.09	**0.0372 (0.0157)**	**0.02**	0.0317 (0.0188)	0.09	0.0228 (0.0165)	0.17	0.0179 (0.0155)	0.25
10y AUROC	0.0121 (0.0151)	0.42	0.0160 (0.0171)	0.35	-0.0231 (0.0202)	0.25	0.0074 (0.0193)	0.7	0.0274 (0.0159)	0.08
Model 3 vs. Model 2										
C-index (10y)	**-0.0930 (0.0211)**	**<0.01**	**-0.0984 (0.0205)**	**<0.01**	**-0.1408 (0.0222)**	**<0.01**	**-0.1042 (0.0196)**	**<0.01**	**-0.1209 (0.0225)**	**<0.01**
1y AUROC	**-0.1482 (0.0486)**	**<0.01**	-0.0945 (0.0513)	0.07	**-0.1154 (0.0563)**	**0.04**	**-0.1432 (0.0439)**	**<0.01**	-0.1257 (0.0678)	0.06
3y AUROC	**-0.1104 (0.0364)**	**<0.01**	**-0.1308 (0.0365)**	**<0.01**	**-0.2043 (0.0385)**	**<0.01**	**-0.1389 (0.0306)**	**<0.01**	**-0.1679 (0.0394)**	**<0.01**
5y AUROC	**-0.1031 (0.0317)**	**<0.01**	**-0.1182 (0.0304)**	**<0.01**	**-0.1575 (0.0338)**	**<0.01**	**-0.1264 (0.0281)**	**<0.01**	**-0.1690 (0.0325)**	**<0.01**
10y AUROC	**-0.1313 (0.0319)**	**<0.01**	**-0.1122 (0.0289)**	**<0.01**	**-0.1992 (0.0328)**	**<0.01**	**-0.1349 (0.0320)**	**<0.01**	**-0.1391 (0.0313)**	**<0.01**
Model 4 vs. Model 2										
C-index (10y)	0.0109 (0.0094)	0.25	0.0082 (0.0098)	0.4	-0.0148 (0.0104)	0.16	-0.0117 (0.0096)	0.22	0.0013 (0.0103)	0.9
1y AUROC	-0.0001 (0.0201)	1	**0.0415 (0.0209)**	**<0.05**	0.0127 (0.0177)	0.47	-0.0072 (0.0142)	0.61	0.0202 (0.0282)	0.47
3y AUROC	0.0260 (0.0138)	0.06	0.0151 (0.0159)	0.34	-0.0018 (0.0148)	0.9	-0.0113 (0.0140)	0.42	0.0032 (0.0160)	0.84
5y AUROC	0.0199 (0.0135)	0.14	0.0153 (0.0139)	0.27	-0.0010 (0.0146)	0.94	-0.0074 (0.0137)	0.59	0.0007 (0.0134)	0.96
10y AUROC	-0.0019 (0.0136)	0.89	-0.0023 (0.0150)	0.88	**-0.0459 (0.0175)**	**<0.01**	-0.0282 (0.0168)	0.09	0.0014 (0.0135)	0.92
Model 4 vs. Model 3										
C-index (10y)	**0.1039 (0.0158)**	**<0.01**	**0.1067 (0.0145)**	**<0.01**	**0.1261 (0.0163)**	**<0.01**	**0.0925 (0.0137)**	**<0.01**	**0.1222 (0.0162)**	**<0.01**
1y AUROC	**0.1481 (0.0356)**	**<0.01**	**0.1360 (0.0388)**	**<0.01**	**0.1281 (0.0450)**	**<0.01**	**0.1360 (0.0368)**	**<0.01**	**0.1459 (0.0468)**	**<0.01**
3y AUROC	**0.1363 (0.0279)**	**<0.01**	**0.1459 (0.0273)**	**<0.01**	**0.2025 (0.0302)**	**<0.01**	**0.1276 (0.0218)**	**<0.01**	**0.1710 (0.0286)**	**<0.01**
5y AUROC	**0.1231 (0.0233)**	**<0.01**	**0.1336 (0.0221)**	**<0.01**	**0.1564 (0.0250)**	**<0.01**	**0.1190 (0.0191)**	**<0.01**	**0.1697 (0.0242)**	**<0.01**
10y AUROC	**0.1294 (0.0250)**	**<0.01**	**0.1098 (0.0186)**	**<0.01**	**0.1533 (0.0219)**	**<0.01**	**0.1067 (0.0202)**	**<0.01**	**0.1405 (0.0226)**	**<0.01**

Note: Bold denotes statistical significance (p<0.05).

Abbreviations: AUROC, area under receiver operating characteristic curve; SE, standard error.

Supplementary Table 2. Performance and calibration of predictive models for overall survival in each outer-loop of the total CRC cohort (N= 2116).

Models	Outer-loop	C-index (10y)	Time-dependent AUROC (95% CI)				IBS (10y)	Time-dependent Brier score (95% CI)			
			1y	3y	5y	10y		1y	3y	5y	10y
Model 1	1	0.74	0.82 (0.75-0.88)	0.80 (0.75-0.85)	0.79 (0.74-0.83)	0.79 (0.75-0.84)	0.15	0.08 (0.06-0.09)	0.14 (0.12-0.16)	0.17 (0.15-0.19)	0.19 (0.17-0.21)
	2	0.72	0.78 (0.70-0.85)	0.77 (0.72-0.83)	0.76 (0.71-0.81)	0.79 (0.74-0.83)	0.15	0.09 (0.07-0.11)	0.15 (0.13-0.17)	0.18 (0.16-0.20)	0.19 (0.17-0.21)
	3	0.75	0.79 (0.71-0.87)	0.82 (0.77-0.87)	0.80 (0.75-0.84)	0.81 (0.76-0.85)	0.14	0.07 (0.05-0.08)	0.12 (0.10-0.14)	0.16 (0.14-0.17)	0.18 (0.17-0.20)
	4	0.73	0.88 (0.82-0.93)	0.79 (0.74-0.84)	0.76 (0.71-0.81)	0.78 (0.73-0.83)	0.15	0.06 (0.05-0.08)	0.15 (0.13-0.17)	0.19 (0.17-0.20)	0.19 (0.18-0.21)
	5	0.74	0.81 (0.72-0.90)	0.82 (0.76-0.87)	0.82 (0.77-0.87)	0.80 (0.75-0.84)	0.14	0.06 (0.04-0.08)	0.12 (0.11-0.14)	0.15 (0.14-0.17)	0.18 (0.16-0.20)
Model 2	1	0.74	0.82 (0.75-0.88)	0.80 (0.75-0.86)	0.79 (0.74-0.84)	0.81 (0.76-0.85)	0.15	0.08 (0.06-0.09)	0.13 (0.12-0.15)	0.17 (0.15-0.19)	0.18 (0.16-0.20)
	2	0.74	0.80 (0.73-0.87)	0.79 (0.74-0.84)	0.78 (0.74-0.83)	0.80 (0.76-0.85)	0.15	0.08 (0.06-0.10)	0.14 (0.12-0.16)	0.17 (0.15-0.19)	0.18 (0.16-0.20)
	3	0.77	0.84 (0.78-0.91)	0.86 (0.82-0.90)	0.83 (0.79-0.87)	0.83 (0.79-0.87)	0.13	0.07 (0.05-0.08)	0.12 (0.10-0.13)	0.15 (0.13-0.17)	0.17 (0.16-0.19)
	4	0.75	0.88 (0.84-0.93)	0.81 (0.76-0.86)	0.79 (0.75-0.83)	0.81 (0.77-0.86)	0.15	0.06 (0.05-0.08)	0.15 (0.13-0.17)	0.18 (0.16-0.20)	0.18 (0.16-0.20)
	5	0.77	0.82 (0.73-0.90)	0.84 (0.79-0.89)	0.84 (0.79-0.88)	0.82 (0.78-0.87)	0.13	0.06 (0.05-0.08)	0.12 (0.10-0.13)	0.14 (0.13-0.16)	0.17 (0.15-0.19)
Model 3	1	0.65	0.67 (0.58-0.75)	0.69 (0.63-0.75)	0.69 (0.63-0.74)	0.68 (0.62-0.73)	0.18	0.09 (0.06-0.11)	0.17 (0.15-0.19)	0.20 (0.18-0.22)	0.23 (0.21-0.25)
	2	0.64	0.71 (0.63-0.78)	0.66 (0.60-0.72)	0.66 (0.61-0.72)	0.69 (0.63-0.75)	0.18	0.09 (0.07-0.11)	0.18 (0.15-0.20)	0.22 (0.20-0.24)	0.22 (0.20-0.25)
	3	0.63	0.73 (0.64-0.81)	0.66 (0.59-0.72)	0.67 (0.62-0.73)	0.63 (0.57-0.69)	0.18	0.07 (0.05-0.09)	0.16 (0.14-0.18)	0.20 (0.18-0.22)	0.24 (0.22-0.26)
	4	0.64	0.74 (0.66-0.82)	0.67 (0.61-0.73)	0.66 (0.61-0.72)	0.68 (0.62-0.74)	0.19	0.07 (0.06-0.09)	0.19 (0.17-0.21)	0.22 (0.20-0.24)	0.23 (0.21-0.25)
	5	0.65	0.69 (0.59-0.79)	0.67 (0.61-0.74)	0.67 (0.61-0.73)	0.68 (0.63-0.74)	0.17	0.06 (0.04-0.08)	0.16 (0.14-0.17)	0.20 (0.18-0.22)	0.23 (0.21-0.24)
Model 4	1	0.75	0.82 (0.75-0.88)	0.83 (0.78-0.88)	0.81 (0.77-0.86)	0.81 (0.76-0.85)	0.15	0.08 (0.06-0.10)	0.13 (0.11-0.15)	0.16 (0.14-0.18)	0.18 (0.16-0.21)
	2	0.74	0.84 (0.78-0.90)	0.81 (0.76-0.86)	0.80 (0.75-0.84)	0.80 (0.76-0.85)	0.15	0.07 (0.06-0.09)	0.14 (0.12-0.16)	0.17 (0.15-0.19)	0.18 (0.16-0.20)
	3	0.76	0.86 (0.80-0.91)	0.86 (0.82-0.90)	0.83 (0.79-0.87)	0.78 (0.73-0.83)	0.13	0.06 (0.05-0.08)	0.12 (0.10-0.13)	0.15 (0.13-0.17)	0.19 (0.17-0.21)
	4	0.74	0.87 (0.82-0.93)	0.80 (0.75-0.85)	0.78 (0.74-0.83)	0.78 (0.74-0.83)	0.15	0.06 (0.05-0.08)	0.15 (0.13-0.17)	0.18 (0.16-0.20)	0.19 (0.17-0.21)
	5	0.77	0.84 (0.77-0.91)	0.84 (0.80-0.89)	0.84 (0.80-0.88)	0.82 (0.78-0.87)	0.13	0.07 (0.05-0.08)	0.12 (0.10-0.14)	0.14 (0.13-0.16)	0.17 (0.15-0.19)

Note: Model 1 based on traditional clinical features, Model 2 based on comprehensive clinical features. Model 3 based on blood DNA methylation, Model 4 combined both clinical features and blood DNA methylation.
Abbreviations: AUROC, area under receiver operating characteristic curve; CI, confidence interval; IBS, integrated Brier score.

Supplementary Table 3. Comparison of 10-year C-index and time-dependent AUROC between different predictive models in each outer-loop of subset 1 (N= 1042).

**Models**	**Outer-loop 1**		**Outer-loop 2**		**Outer-loop 3**		**Outer-loop 4**		**Outer-loop 5**	
	**ΔValue (SE)**	***p***	**ΔValue (SE)**	***p***	**ΔValue (SE)**	***p***	**ΔValue (SE)**	***p***	**ΔValue (SE)**	***p***
Model 2 vs. Model 1										
C-index (10y)	0.0135 (0.0140)	0.33	0.0122 (0.0119)	0.31	**0.0281 (0.0124)**	**0.02**	0.0095 (0.0112)	0.4	0.0123 (0.0116)	0.29
1y AUROC	-0.0021 (0.0301)	0.94	0.0203 (0.0266)	0.45	**0.0881 (0.0417)**	**0.03**	-0.0151 (0.0123)	0.22	0.0087 (0.0358)	0.81
3y AUROC	0.0130 (0.0187)	0.49	0.0235 (0.0178)	0.19	0.0416 (0.0226)	0.07	0.0153 (0.0186)	0.41	0.0306 (0.0194)	0.11
5y AUROC	0.0180 (0.0197)	0.36	0.0229 (0.0175)	0.19	0.0353 (0.0185)	0.06	0.0128 (0.0172)	0.46	0.0002 (0.0155)	0.99
10y AUROC	-0.0090 (0.0229)	0.69	0.0060 (0.0193)	0.75	0.0273 (0.0190)	0.15	0.0255 (0.0183)	0.16	0.0046 (0.0198)	0.82
Model 3 vs. Model 1										
C-index (10y)	**-0.1001 (0.0370)**	**<0.01**	**-0.1314 (0.0304)**	**<0.01**	**-0.1076 (0.0365)**	**<0.01**	**-0.0699 (0.0318)**	**0.03**	**-0.0945 (0.0333)**	**<0.01**
1y AUROC	**-0.1661 (0.0839)**	**<0.05**	**-0.1673 (0.0742)**	**0.02**	0.0147 (0.0973)	0.88	-0.1286 (0.0675)	0.06	-0.0778 (0.1146)	0.5
3y AUROC	-0.1151 (0.0683)	0.09	**-0.1752 (0.0530)**	**<0.01**	**-0.1333 (0.0623)**	**0.03**	**-0.0987 (0.0491)**	**0.04**	**-0.1178 (0.0587)**	**0.04**
5y AUROC	**-0.1183 (0.0567)**	**0.04**	**-0.1634 (0.0448)**	**<0.01**	**-0.1156 (0.0548)**	**0.03**	-0.0843 (0.0446)	0.06	**-0.1662 (0.0456)**	**<0.01**
10y AUROC	**-0.1152 (0.0537)**	**0.03**	**-0.1162 (0.0502)**	**0.02**	-0.0936 (0.0511)	0.07	-0.0952 (0.0539)	0.08	-0.0752 (0.0498)	0.13
Model 4 vs. Model 1										
C-index (10y)	-0.0019 (0.0193)	0.92	-0.0095 (0.0184)	0.6	0.0083 (0.0238)	0.73	0.0166 (0.0196)	0.4	0.0097 (0.0207)	0.64
1y AUROC	-0.0081 (0.0454)	0.86	0.0179 (0.0329)	0.59	0.1068 (0.0633)	0.09	0.0034 (0.0269)	0.9	0.0778 (0.0603)	0.2
3y AUROC	0.0262 (0.0326)	0.42	0.0092 (0.0265)	0.73	0.0573 (0.0369)	0.12	0.0364 (0.0298)	0.22	0.0179 (0.0328)	0.59
5y AUROC	0.0025 (0.0288)	0.93	-0.0044 (0.0260)	0.86	0.0502 (0.0340)	0.14	0.0193 (0.0276)	0.48	-0.0159 (0.0269)	0.55
10y AUROC	-0.0209 (0.0279)	0.45	-0.0192 (0.0333)	0.56	0.0056 (0.0370)	0.88	0.0168 (0.0336)	0.62	0.0154 (0.0312)	0.62
Model 3 vs. Model 2										
C-index (10y)	**-0.1136 (0.0382)**	**<0.01**	**-0.1436 (0.0306)**	**<0.01**	**-0.1356 (0.0324)**	**<0.01**	**-0.0794 (0.0310)**	**0.01**	**-0.1068 (0.0326)**	**<0.01**
1y AUROC	-0.1640 (0.0908)	0.07	**-0.1875 (0.0725)**	**<0.01**	-0.0734 (0.0788)	0.35	-0.1135 (0.0661)	0.09	-0.0865 (0.1151)	0.45
3y AUROC	-0.1281 (0.0697)	0.07	**-0.1987 (0.0535)**	**<0.01**	**-0.1748 (0.0532)**	**<0.01**	**-0.1139 (0.0459)**	**0.01**	**-0.1484 (0.0579)**	**0.01**
5y AUROC	**-0.1364 (0.0583)**	**0.02**	**-0.1864 (0.0455)**	**<0.01**	**-0.1509 (0.0492)**	**<0.01**	**-0.0971 (0.0427)**	**0.02**	**-0.1664 (0.0448)**	**<0.01**
10y AUROC	-0.1063 (0.0556)	0.06	**-0.1222 (0.0468)**	**<0.01**	**-0.1209 (0.0488)**	**0.01**	**-0.1207 (0.0528)**	**0.02**	**-0.0798 (0.0479)**	**0.1**
Model 4 vs. Model 2										
C-index (10y)	-0.0154 (0.0210)	0.46	-0.0217 (0.0186)	0.24	-0.0198 (0.0190)	0.3	0.0071 (0.0182)	0.7	-0.0026 (0.0208)	0.9
1y AUROC	-0.0060 (0.0505)	0.91	-0.0024 (0.0353)	0.95	0.0187 (0.0330)	0.57	0.0185 (0.0241)	0.44	0.0691 (0.0633)	0.27
3y AUROC	0.0132 (0.0347)	0.7	-0.0143 (0.0276)	0.6	0.0157 (0.0233)	0.5	0.0211 (0.0254)	0.41	-0.0128 (0.0343)	0.71
5y AUROC	-0.0155 (0.0321)	0.63	-0.0273 (0.0261)	0.3	0.0150 (0.0252)	0.55	0.0065 (0.0247)	0.79	-0.0162 (0.0269)	0.55
10y AUROC	-0.0119 (0.0295)	0.69	-0.0252 (0.0285)	0.38	-0.0216 (0.0320)	0.5	-0.0087 (0.0320)	0.79	0.0109 (0.0310)	0.73
Model 4 vs. Model 3										
C-index (10y)	**0.0982 (0.0252)**	**<0.01**	**0.1218 (0.0209)**	**<0.01**	**0.1158 (0.0225)**	**<0.01**	**0.0865 (0.0195)**	**<0.01**	**0.1042 (0.0202)**	**<0.01**
1y AUROC	**0.1580 (0.0620)**	**0.01**	**0.1852 (0.0587)**	**<0.01**	0.0920 (0.0604)	0.13	**0.1320 (0.0527)**	**0.01**	**0.1557 (0.0668)**	**0.02**
3y AUROC	**0.1413 (0.0468)**	**<0.01**	**0.1844 (0.0397)**	**<0.01**	**0.1905 (0.0419)**	**<0.01**	**0.1350 (0.0295)**	**<0.01**	**0.1357 (0.0363)**	**<0.01**
5y AUROC	**0.1208 (0.0375)**	**<0.01**	**0.1590 (0.0304)**	**<0.01**	**0.1658 (0.0353)**	**<0.01**	**0.1036 (0.0266)**	**<0.01**	**0.1503 (0.0282)**	**<0.01**
10y AUROC	**0.0943 (0.0357)**	**<0.01**	**0.0970 (0.0274)**	**<0.01**	**0.0992 (0.0268)**	**<0.01**	**0.1120 (0.0301)**	**<0.01**	**0.0907 (0.0271)**	**<0.01**

Note: Subset 1 includes patients with blood samples collected ≥1-month after surgery. Bold denotes statistical significance (p<0.05).
Abbreviations: AUROC, area under receiver operating characteristic curve; SE, standard error.

Supplementary Table 4. Performance and calibration of predictive models for overall survival in each outer-loop of subset 1 (N= 1042).

Models	Outer-loop	C-index (10y)	Time-dependent AUROC (95% CI)				IBS (10y)	Time-dependent Brier score (95% CI)			
			1y	3y	5y	10y		1y	3y	5y	10y
Model 1	1	0.75	0.87 (0.79-0.95)	0.83 (0.75-0.91)	0.78 (0.70-0.85)	0.79 (0.71-0.87)	0.14	0.06 (0.04-0.08)	0.12 (0.09-0.14)	0.17 (0.14-0.20)	0.20 (0.16-0.24)
	2	0.74	0.81 (0.71-0.92)	0.80 (0.72-0.87)	0.78 (0.72-0.85)	0.79 (0.72-0.87)	0.14	0.07 (0.04-0.10)	0.13 (0.10-0.15)	0.16 (0.14-0.19)	0.19 (0.17-0.22)
	3	0.73	0.72 (0.58-0.85)	0.81 (0.74-0.88)	0.78 (0.71-0.85)	0.75 (0.68-0.83)	0.14	0.06 (0.04-0.09)	0.11 (0.09-0.14)	0.15 (0.13-0.18)	0.20 (0.18-0.22)
	4	0.73	0.91 (0.84-0.97)	0.80 (0.72-0.87)	0.75 (0.68-0.82)	0.78 (0.70-0.85)	0.16	0.06 (0.04-0.09)	0.16 (0.12-0.19)	0.19 (0.16-0.21)	0.20 (0.17-0.22)
	5	0.72	0.78 (0.62-0.93)	0.76 (0.67-0.86)	0.82 (0.75-0.89)	0.78 (0.71-0.86)	0.14	0.06 (0.03-0.08)	0.13 (0.11-0.16)	0.16 (0.13-0.18)	0.19 (0.16-0.21)
Model 2	1	0.76	0.87 (0.79-0.94)	0.84 (0.76-0.92)	0.80 (0.72-0.87)	0.79 (0.70-0.87)	0.14	0.06 (0.04-0.08)	0.12 (0.09-0.14)	0.16 (0.14-0.19)	0.20 (0.16-0.24)
	2	0.75	0.83 (0.75-0.92)	0.82 (0.75-0.89)	0.81 (0.74-0.87)	0.80 (0.72-0.87)	0.14	0.07 (0.04-0.10)	0.12 (0.10-0.15)	0.16 (0.13-0.18)	0.19 (0.16-0.21)
	3	0.75	0.81 (0.71-0.90)	0.85 (0.80-0.91)	0.82 (0.76-0.88)	0.78 (0.71-0.85)	0.13	0.06 (0.04-0.08)	0.11 (0.09-0.13)	0.15 (0.12-0.17)	0.19 (0.17-0.21)
	4	0.74	0.89 (0.82-0.96)	0.81 (0.74-0.88)	0.76 (0.69-0.83)	0.80 (0.73-0.87)	0.15	0.06 (0.04-0.09)	0.15 (0.12-0.19)	0.18 (0.15-0.22)	0.19 (0.16-0.22)
	5	0.74	0.79 (0.63-0.94)	0.79 (0.70-0.88)	0.82 (0.75-0.89)	0.79 (0.72-0.86)	0.14	0.06 (0.04-0.08)	0.13 (0.10-0.15)	0.15 (0.13-0.18)	0.18 (0.15-0.21)
Model 3	1	0.65	0.70 (0.55-0.85)	0.71 (0.60-0.82)	0.66 (0.57-0.75)	0.68 (0.59-0.77)	0.16	0.07 (0.04-0.09)	0.13 (0.10-0.16)	0.18 (0.16-0.21)	0.23 (0.20-0.26)
	2	0.61	0.65 (0.53-0.77)	0.62 (0.53-0.71)	0.62 (0.54-0.70)	0.68 (0.58-0.77)	0.18	0.08 (0.05-0.11)	0.17 (0.14-0.20)	0.21 (0.18-0.24)	0.23 (0.20-0.27)
	3	0.62	0.73 (0.61-0.85)	0.68 (0.59-0.77)	0.67 (0.59-0.74)	0.66 (0.57-0.75)	0.17	0.06 (0.04-0.09)	0.15 (0.12-0.17)	0.19 (0.17-0.22)	0.24 (0.21-0.27)
	4	0.66	0.78 (0.66-0.89)	0.70 (0.62-0.77)	0.67 (0.59-0.74)	0.68 (0.59-0.77)	0.18	0.07 (0.05-0.10)	0.19 (0.16-0.22)	0.22 (0.19-0.25)	0.23 (0.20-0.26)
	5	0.63	0.70 (0.55-0.85)	0.64 (0.54-0.74)	0.65 (0.57-0.74)	0.71 (0.62-0.80)	0.18	0.06 (0.03-0.08)	0.16 (0.13-0.19)	0.21 (0.18-0.24)	0.22 (0.19-0.25)
Model 4	1	0.75	0.86 (0.76-0.96)	0.85 (0.78-0.93)	0.78 (0.70-0.86)	0.77 (0.69-0.85)	0.14	0.06 (0.04-0.08)	0.11 (0.09-0.14)	0.17 (0.14-0.20)	0.20 (0.16-0.24)
	2	0.73	0.83 (0.73-0.93)	0.81 (0.72-0.89)	0.78 (0.71-0.85)	0.77 (0.69-0.86)	0.14	0.06 (0.04-0.09)	0.13 (0.10-0.16)	0.17 (0.13-0.20)	0.20 (0.15-0.24)
	3	0.74	0.82 (0.73-0.92)	0.87 (0.81-0.93)	0.83 (0.77-0.89)	0.76 (0.68-0.84)	0.13	0.06 (0.04-0.08)	0.10 (0.08-0.13)	0.14 (0.12-0.16)	0.20 (0.16-0.24)
	4	0.75	0.91 (0.85-0.97)	0.83 (0.77-0.89)	0.77 (0.70-0.84)	0.79 (0.72-0.87)	0.15	0.05 (0.03-0.07)	0.14 (0.11-0.17)	0.18 (0.15-0.21)	0.19 (0.16-0.22)
	5	0.73	0.85 (0.75-0.96)	0.78 (0.69-0.87)	0.80 (0.73-0.87)	0.80 (0.73-0.87)	0.14	0.06 (0.04-0.08)	0.13 (0.10-0.16)	0.16 (0.13-0.18)	0.18 (0.15-0.21)

Note: Subset 1 includes patients with blood samples collected ≥1-month after surgery. Model 1 based on traditional clinical features, Model 2 based on comprehensive clinical features. Model 3 based on blood DNA methylation, Model 4 combined both clinical features and blood DNA methylation.
Abbreviations: AUROC, area under receiver operating characteristic curve; CI, confidence interval; IBS, integrated Brier score.

Supplementary Table 5. Comparison of 10-year C-index and time-dependent AUROC between different predictive models in each outer-loop of subset 2 (N= 1434).

**Models**	**Outer-loop 1**		**Outer-loop 2**		**Outer-loop 3**		**Outer-loop 4**		**Outer-loop 5**	
	**ΔValue (SE)**	***p***	**ΔValue (SE)**	***p***	**ΔValue (SE)**	***p***	**ΔValue (SE)**	***p***	**ΔValue (SE)**	***p***
Model 2 vs. Model 1										
C-index (10y)	0.0132 (0.0143)	0.35	0.0214 (0.0145)	0.14	**0.0323 (0.0134)**	**0.02**	0.0161 (0.0115)	0.16	**0.0442 (0.0134)**	**<0.01**
1y AUROC	-0.0043 (0.0330)	0.9	0.0306 (0.0425)	0.47	0.0947 (0.0550)	0.08	0.0050 (0.0308)	0.87	0.0599 (0.0445)	0.18
3y AUROC	0.0085 (0.0237)	0.72	0.0287 (0.0267)	0.28	**0.0772 (0.0290)**	**<0.01**	0.0129 (0.0185)	0.48	**0.0592 (0.0265)**	**0.03**
5y AUROC	0.0199 (0.0212)	0.35	0.0231 (0.0219)	0.29	**0.0614 (0.0220)**	**<0.01**	0.0268 (0.0171)	0.12	**0.0424 (0.0194)**	**0.03**
10y AUROC	0.0300 (0.0208)	0.15	0.0306 (0.0198)	0.12	0.0161 (0.0184)	0.38	0.0211 (0.0172)	0.22	**0.0456 (0.0177)**	**0.01**
Model 3 vs. Model 1										
C-index (10y)	-0.0373 (0.0252)	0.14	-0.0286 (0.0209)	0.17	**-0.0708 (0.0249)**	**<0.01**	**-0.0718 (0.0273)**	**<0.01**	-0.0101 (0.0277)	0.72
1y AUROC	-0.0735 (0.0656)	0.26	0.0078 (0.0434)	0.86	-0.0436 (0.0563)	0.44	-0.0922 (0.0765)	0.23	-0.0100 (0.0783)	0.9
3y AUROC	-0.0264 (0.0437)	0.55	-0.0194 (0.0396)	0.62	-0.0359 (0.0521)	0.49	-0.0855 (0.0459)	0.06	-0.0097 (0.0492)	0.84
5y AUROC	-0.0283 (0.0379)	0.46	-0.0175 (0.0319)	0.58	-0.0347 (0.0400)	0.39	-0.0586 (0.0397)	0.14	-0.0410 (0.0403)	0.31
10y AUROC	-0.0613 (0.0372)	0.1	-0.0605 (0.0342)	0.08	**-0.1413 (0.0356)**	**<0.01**	**-0.1153 (0.0440)**	**<0.01**	-0.0041 (0.0390)	0.92
Model 4 vs. Model 1										
C-index (10y)	0.0052 (0.0188)	0.78	-0.0095 (0.0176)	0.59	-0.0348 (0.0197)	0.08	-0.0316 (0.0206)	0.13	0.0214 (0.0200)	0.29
1y AUROC	-0.0197 (0.0515)	0.7	0.0072 (0.0398)	0.86	0.0000 (0.0469)	1	-0.0171 (0.0540)	0.75	0.0113 (0.0601)	0.85
3y AUROC	0.0261 (0.0333)	0.43	0.0036 (0.0343)	0.92	0.0146 (0.0392)	0.71	-0.0457 (0.0346)	0.19	0.0371 (0.0351)	0.29
5y AUROC	0.0190 (0.0280)	0.5	0.0046 (0.0269)	0.86	0.0131 (0.0303)	0.67	-0.0272 (0.0302)	0.37	0.0097 (0.0290)	0.74
10y AUROC	0.0017 (0.0267)	0.95	-0.0364 (0.0275)	0.19	**-0.0931 (0.0288)**	**<0.01**	-0.0607 (0.0340)	0.07	0.0221 (0.0281)	0.43
Model 3 vs. Model 2										
C-index (10y)	**-0.0505 (0.0255)**	**<0.05**	**-0.0500 (0.0228)**	**0.03**	**-0.1031 (0.0251)**	**<0.01**	**-0.0879 (0.0255)**	**<0.01**	**-0.0543 (0.0273)**	**<0.05**
1y AUROC	-0.0691 (0.0758)	0.36	-0.0228 (0.0596)	0.7	**-0.1383 (0.0698)**	**<0.05**	-0.0972 (0.0650)	0.13	-0.0699 (0.0861)	0.42
3y AUROC	-0.0349 (0.0410)	0.4	-0.0481 (0.0407)	0.24	**-0.1132 (0.0500)**	**0.02**	**-0.0985 (0.0419)**	**0.02**	-0.0689 (0.0515)	0.18
5y AUROC	-0.0482 (0.0373)	0.2	-0.0405 (0.0343)	0.24	**-0.0961 (0.0410)**	**0.02**	**-0.0854 (0.0376)**	**0.02**	**-0.0834 (0.0392)**	**0.03**
10y AUROC	**-0.0913 (0.0370)**	**0.01**	**-0.0911 (0.0343)**	**<0.01**	**-0.1574 (0.0360)**	**<0.01**	**-0.1364 (0.0421)**	**<0.01**	-0.0497 (0.0380)	0.19
Model 4 vs. Model 2										
C-index (10y)	-0.0080 (0.0190)	0.67	-0.0309 (0.0193)	0.11	**-0.0671 (0.0194)**	**<0.01**	**-0.0478 (0.0192)**	**0.01**	-0.0228 (0.0195)	0.24
1y AUROC	-0.0153 (0.0602)	0.8	-0.0233 (0.0546)	0.67	-0.0947 (0.0603)	0.12	-0.0222 (0.0443)	0.62	-0.0486 (0.0676)	0.47
3y AUROC	0.0176 (0.0298)	0.55	-0.0251 (0.0347)	0.47	-0.0626 (0.0365)	0.09	-0.0586 (0.0311)	0.06	-0.0220 (0.0394)	0.58
5y AUROC	-0.0009 (0.0276)	0.97	-0.0184 (0.0293)	0.53	-0.0483 (0.0310)	0.12	-0.0540 (0.0286)	0.06	-0.0328 (0.0270)	0.23
10y AUROC	-0.0282 (0.0262)	0.28	-0.0670 (0.0277)	0.02	**-0.1093 (0.0282)**	**<0.01**	**-0.0819 (0.0324)**	**0.01**	-0.0234 (0.0260)	0.37
Model 4 vs. Model 3										
C-index (10y)	**0.0424 (0.0113)**	**<0.01**	**0.0191 (0.0078)**	**0.01**	**0.0359 (0.0100)**	**<0.01**	**0.0401 (0.0108)**	**<0.01**	**0.0315 (0.0148)**	**0.03**
1y AUROC	0.0538 (0.0306)	0.08	-0.0006 (0.0188)	0.98	0.0436 (0.0253)	0.09	**0.0751 (0.0308)**	**0.01**	0.0213 (0.0373)	0.57
3y AUROC	**0.0525 (0.0191)**	**<0.01**	0.0230 (0.0137)	0.09	**0.0506 (0.0206)**	**0.01**	**0.0399 (0.0188)**	**0.03**	0.0468 (0.0240)	0.05
5y AUROC	**0.0473 (0.0167)**	**<0.01**	0.0221 (0.0115)	0.05	**0.0478 (0.0160)**	**<0.01**	0.0314 (0.0166)	0.06	**0.0507 (0.0224)**	**0.02**
10y AUROC	**0.0631 (0.0174)**	**<0.01**	0.0241 (0.0138)	0.08	**0.0481 (0.0149)**	**<0.01**	**0.0546 (0.0172)**	**<0.01**	0.0262 (0.0218)	0.23

Note: Subset 2 includes patients with TNM stage II-III. Bold denotes statistical significance (p<0.05).
Abbreviations: AUROC, area under receiver operating characteristic curve; SE, standard error

Supplementary Table 6. Performance and calibration of predictive models for overall survival in each outer-loop of subset 2 (N= 1434).

Models	Outer-loop	C-index (10y)	Time-dependent AUROC (95% CI)				IBS (10y)	Time-dependent Brier score (95% CI)			
			1y	3y	5y	10y		1y	3y	5y	10y
Model 1	1	0.68	0.76 (0.65-0.88)	0.72 (0.63-0.80)	0.70 (0.63-0.77)	0.73 (0.66-0.80)	0.16	0.06 (0.03-0.08)	0.14 (0.11-0.16)	0.18 (0.16-0.21)	0.21 (0.19-0.23)
	2	0.66	0.68 (0.56-0.80)	0.67 (0.59-0.76)	0.68 (0.61-0.75)	0.73 (0.66-0.79)	0.17	0.07 (0.04-0.09)	0.15 (0.12-0.17)	0.20 (0.17-0.22)	0.22 (0.20-0.24)
	3	0.68	0.72 (0.60-0.85)	0.68 (0.60-0.76)	0.68 (0.61-0.75)	0.73 (0.67-0.80)	0.14	0.04 (0.02-0.06)	0.12 (0.10-0.14)	0.17 (0.15-0.19)	0.21 (0.20-0.23)
	4	0.67	0.84 (0.75-0.93)	0.68 (0.60-0.76)	0.65 (0.59-0.72)	0.73 (0.67-0.80)	0.17	0.05 (0.03-0.07)	0.16 (0.13-0.19)	0.21 (0.18-0.23)	0.21 (0.19-0.23)
	5	0.67	0.73 (0.61-0.84)	0.69 (0.60-0.78)	0.71 (0.64-0.78)	0.71 (0.64-0.77)	0.14	0.04 (0.02-0.05)	0.12 (0.10-0.14)	0.17 (0.15-0.19)	0.22 (0.20-0.24)
Model 2	1	0.69	0.76 (0.64-0.88)	0.72 (0.64-0.81)	0.72 (0.65-0.79)	0.76 (0.69-0.82)	0.15	0.06 (0.03-0.08)	0.13 (0.11-0.16)	0.18 (0.16-0.20)	0.20 (0.18-0.22)
	2	0.68	0.71 (0.59-0.83)	0.70 (0.63-0.78)	0.70 (0.64-0.77)	0.76 (0.69-0.82)	0.16	0.06 (0.04-0.09)	0.14 (0.12-0.17)	0.19 (0.17-0.22)	0.21 (0.18-0.23)
	3	0.71	0.82 (0.72-0.92)	0.76 (0.68-0.83)	0.74 (0.67-0.81)	0.75 (0.69-0.81)	0.14	0.04 (0.02-0.06)	0.11 (0.09-0.13)	0.16 (0.14-0.18)	0.20 (0.18-0.22)
	4	0.69	0.84 (0.78-0.91)	0.69 (0.62-0.77)	0.68 (0.62-0.75)	0.76 (0.69-0.82)	0.16	0.05 (0.03-0.07)	0.16 (0.13-0.18)	0.20 (0.18-0.23)	0.20 (0.18-0.23)
	5	0.72	0.79 (0.68-0.89)	0.75 (0.67-0.83)	0.75 (0.69-0.82)	0.75 (0.69-0.81)	0.13	0.03 (0.02-0.05)	0.11 (0.09-0.13)	0.16 (0.14-0.18)	0.20 (0.19-0.22)
Model 3	1	0.64	0.69 (0.56-0.82)	0.69 (0.61-0.77)	0.67 (0.60-0.74)	0.67 (0.59-0.74)	0.17	0.06 (0.04-0.08)	0.14 (0.12-0.17)	0.20 (0.17-0.22)	0.24 (0.21-0.27)
	2	0.63	0.69 (0.57-0.81)	0.65 (0.57-0.74)	0.66 (0.59-0.73)	0.67 (0.59-0.74)	0.18	0.07 (0.04-0.09)	0.17 (0.14-0.19)	0.21 (0.18-0.24)	0.25 (0.21-0.28)
	3	0.61	0.68 (0.56-0.80)	0.64 (0.55-0.74)	0.64 (0.57-0.72)	0.59 (0.52-0.67)	0.17	0.05 (0.03-0.07)	0.13 (0.11-0.16)	0.19 (0.17-0.22)	0.27 (0.24-0.30)
	4	0.6	0.75 (0.64-0.85)	0.60 (0.52-0.67)	0.60 (0.53-0.67)	0.62 (0.54-0.69)	0.2	0.06 (0.04-0.08)	0.19 (0.16-0.22)	0.25 (0.22-0.28)	0.27 (0.23-0.30)
	5	0.67	0.72 (0.57-0.86)	0.68 (0.59-0.78)	0.67 (0.60-0.75)	0.70 (0.64-0.77)	0.15	0.04 (0.02-0.05)	0.12 (0.09-0.14)	0.17 (0.15-0.19)	0.22 (0.20-0.24)
Model 4	1	0.68	0.74 (0.63-0.86)	0.74 (0.67-0.82)	0.72 (0.65-0.79)	0.73 (0.66-0.80)	0.16	0.06 (0.04-0.08)	0.14 (0.11-0.16)	0.19 (0.16-0.21)	0.21 (0.18-0.24)
	2	0.65	0.69 (0.57-0.81)	0.68 (0.59-0.76)	0.68 (0.61-0.76)	0.69 (0.62-0.76)	0.18	0.07 (0.04-0.09)	0.17 (0.14-0.19)	0.21 (0.18-0.24)	0.24 (0.21-0.28)
	3	0.65	0.72 (0.60-0.85)	0.69 (0.61-0.78)	0.69 (0.62-0.76)	0.64 (0.57-0.71)	0.16	0.05 (0.03-0.07)	0.13 (0.10-0.15)	0.18 (0.16-0.21)	0.25 (0.22-0.28)
	4	0.64	0.82 (0.75-0.89)	0.64 (0.56-0.71)	0.63 (0.56-0.70)	0.67 (0.60-0.74)	0.19	0.05 (0.04-0.07)	0.18 (0.15-0.21)	0.24 (0.21-0.27)	0.25 (0.21-0.28)
	5	0.69	0.74 (0.59-0.88)	0.73 (0.65-0.81)	0.72 (0.65-0.79)	0.73 (0.66-0.79)	0.14	0.04 (0.02-0.05)	0.12 (0.09-0.14)	0.17 (0.14-0.19)	0.21 (0.19-0.24)

Note: Subset 2 includes patients with TNM stage II-III. Model 1 based on traditional clinical features, Model 2 based on comprehensive clinical features. Model 3 based on blood DNA methylation, Model 4 combined both clinical features and blood DNA methylation.
Abbreviations: AUROC, area under receiver operating characteristic curve; CI, confidence interval; IBS, integrated Brier score.

Supplementary Table 7. Performance and calibration of predictive models for CSS of in the total CRC cohort and two subsets.

Models	C-index (10y)	Time-dependent AUROC				IBS (10y)	Time-dependent Brier score			
		1y	3y	5y	10y		1y	3y	5y	10y
**Total CRC patient (N= 2116)**										
Model 1	0.75 (0.73-0.80)	0.89 (0.84-0.90)	0.84 (0.81-0.86)	0.80 (0.79-0.85)	0.81 (0.79-0.86)	0.17 (0.17-0.19)	0.05 (0.05-0.07)	0.11 (0.10-0.12)	0.15 (0.13-0.15)	0.17 (0.17-0.19)
Model 2	0.77 (0.75-0.81)	0.89 (0.88-0.91)	0.85 (0.83-0.88)	0.81 (0.81-0.87)	0.83 (0.81-0.88)	0.17 (0.15-0.18)	0.05 (0.05-0.06)	0.11 (0.10-0.12)	0.14 (0.12-0.15)	0.17 (0.15-0.18)
Model 3	0.61 (0.58-0.65)	0.71 (0.67-0.77)	0.66 (0.62-0.68)	0.66 (0.64-0.68)	0.66 (0.58-0.69)	0.25 (0.24-0.25)	0.07 (0.05-0.07)	0.15 (0.13-0.16)	0.19 (0.18-0.20)	0.25 (0.24-0.25)
Model 4	0.77 (0.75-0.82)	0.89 (0.88-0.90)	0.85 (0.81-0.87)	0.82 (0.81-0.86)	0.81 (0.80-0.88)	0.18 (0.15-0.18)	0.06 (0.05-0.06)	0.11 (0.10-0.12)	0.14 (0.12-0.15)	0.18 (0.15-0.18)
**Subset 1 (N= 1042)**										
Model 1	0.75 (0.72-0.81)	0.90 (0.86-0.90)	0.83 (0.80-0.88)	0.80 (0.74-0.88)	0.80 (0.77-0.87)	0.18 (0.17-0.21)	0.04 (0.04-0.06)	0.10 (0.09-0.12)	0.14 (0.11-0.16)	0.18 (0.17-0.21)
Model 2	0.77 (0.75-0.82)	0.89 (0.88-0.92)	0.84 (0.82-0.90)	0.82 (0.77-0.89)	0.81 (0.80-0.86)	0.17 (0.16-0.22)	0.05 (0.04-0.06)	0.10 (0.08-0.12)	0.13 (0.11-0.15)	0.17 (0.16-0.22)
Model 3	0.59 (0.57-0.65)	0.64 (0.63-0.77)	0.63 (0.61-0.69)	0.63 (0.59-0.67)	0.65 (0.61-0.72)	0.24 (0.22-0.25)	0.05 (0.05-0.07)	0.13 (0.12-0.16)	0.19 (0.17-0.19)	0.24 (0.22-0.25)
Model 4	0.76 (0.73-0.79)	0.87 (0.84-0.91)	0.84 (0.81-0.89)	0.79 (0.76-0.87)	0.80 (0.77-0.86)	0.18 (0.16-0.21)	0.05 (0.04-0.05)	0.11 (0.09-0.12)	0.15 (0.11-0.16)	0.18 (0.16-0.21)
**Subset 2 (N= 1434)**										
Model 1	0.63 (0.57-0.67)	0.78 (0.70-0.87)	0.65 (0.62-0.71)	0.66 (0.63-0.70)	0.68 (0.64-0.72)	0.22 (0.21-0.22)	0.03 (0.02-0.04)	0.10 (0.09-0.13)	0.16 (0.14-0.17)	0.22 (0.21-0.22)
Model 2	0.64 (0.59-0.71)	0.82 (0.78-0.85)	0.70 (0.68-0.72)	0.68 (0.66-0.74)	0.70 (0.67-0.76)	0.21 (0.20-0.21)	0.03 (0.02-0.04)	0.10 (0.08-0.13)	0.16 (0.13-0.18)	0.21 (0.20-0.21)
Model 3	0.58 (0.54-0.68)	0.69 (0.66-0.78)	0.63 (0.58-0.70)	0.60 (0.58-0.66)	0.60 (0.53-0.72)	0.26 (0.21-0.29)	0.04 (0.02-0.05)	0.12 (0.09-0.17)	0.18 (0.14-0.22)	0.26 (0.21-0.29)
Model 4	0.60 (0.55-0.70)	0.72 (0.68-0.85)	0.65 (0.61-0.72)	0.63 (0.60-0.71)	0.63 (0.56-0.74)	0.26 (0.21-0.27)	0.04 (0.02-0.05)	0.11 (0.09-0.16)	0.18 (0.14-0.22)	0.26 (0.21-0.27)

Note: Subset 1 includes patients with blood samples collected ≥1-month after surgery. Subset 2 includes patients with TNM stage II-III. Model 1 based on traditional clinical features, Model 2 based on comprehensive clinical features, Model 3 based on blood DNA methylation, Model 4 combined both clinical features and blood DNA methylation. The values are presented as the mean (range) across 5-fold outer loops.

Abbreviations: CSS, CRC-specific survival; AUROC, area under receiver operating characteristic curve; IBS, integrated Brier score.

Supplementary Table 8. Comparison of 10-year C-index and time-dependent AUROC between different predictive models for CSS in each outer-loop of the total CRC cohort (N= 2116).

**Models**	**Outer-loop 1**		**Outer-loop 2**		**Outer-loop 3**		**Outer-loop 4**		**Outer-loop 5**	
	**ΔValue (SE)**	***p***	**ΔValue (SE)**	***p***	**ΔValue (SE)**	***p***	**ΔValue (SE)**	***p***	**ΔValue (SE)**	***p***
Model 2 vs. Model 1										
C-index (10y)	0.0076 (0.0107)	0.48	0.0202 (0.0105)	0.05	0.0111 (0.0091)	0.22	0.0133 (0.0092)	0.15	0.0096 (0.0086)	0.26
1y AUROC	0.0066 (0.0157)	0.68	0.0409 (0.0184)	0.03	0.0288 (0.0186)	0.12	0.0011 (0.0096)	0.91	-0.0098 (0.0167)	0.56
3y AUROC	0.0032 (0.0127)	0.8	0.0253 (0.0151)	0.09	**0.0236 (0.0119)**	**<0.05**	0.0155 (0.0136)	0.25	0.0207 (0.0118)	0.08
5y AUROC	0.0121 (0.0141)	0.39	0.0218 (0.0133)	0.1	0.0181 (0.0111)	0.1	0.0202 (0.0123)	0.1	0.0126 (0.0104)	0.23
10y AUROC	0.0118 (0.0136)	0.39	0.0165 (0.0128)	0.2	0.0102 (0.0112)	0.36	0.0265 (0.0121)	0.03	0.0165 (0.0103)	0.11
Model 3 vs. Model 1										
C-index (10y)	**-0.1404 (0.0295)**	**<0.01**	**-0.1373 (0.0315)**	**<0.01**	**-0.2106 (0.0317)**	**<0.01**	**-0.1091 (0.0265)**	**<0.01**	**-0.1612 (0.0304)**	**<0.01**
1y AUROC	**-0.2278 (0.0518)**	**<0.01**	**-0.1292 (0.0592)**	**0.03**	**-0.1588 (0.0635)**	**0.01**	**-0.1135 (0.0502)**	**0.02**	**-0.2158 (0.0606)**	**<0.01**
3y AUROC	**-0.1785 (0.0413)**	**<0.01**	**-0.1665 (0.0493)**	**<0.01**	**-0.2339 (0.0443)**	**<0.01**	**-0.1365 (0.0351)**	**<0.01**	**-0.1591 (0.0442)**	**<0.01**
5y AUROC	**-0.1334 (0.0376)**	**<0.01**	**-0.1510 (0.0398)**	**<0.01**	**-0.2171 (0.0384)**	**<0.01**	**-0.1154 (0.0327)**	**<0.01**	**-0.1974 (0.0370)**	**<0.01**
10y AUROC	**-0.1597 (0.0387)**	**<0.01**	**-0.1349 (0.0379)**	**<0.01**	**-0.2714 (0.0421)**	**<0.01**	**-0.1314 (0.0370)**	**<0.01**	**-0.1695 (0.0372)**	**<0.01**
Model 4 vs. Model 1										
C-index (10y)	0.0181 (0.0136)	0.18	0.0273 (0.0153)	0.07	-0.0136 (0.0141)	0.34	0.0016 (0.0139)	0.91	0.0129 (0.0132)	0.33
1y AUROC	-0.0210 (0.0244)	0.39	**0.0635 (0.0201)**	**<0.01**	0.0210 (0.0206)	0.31	0.0062 (0.0189)	0.74	-0.0103 (0.0192)	0.59
3y AUROC	0.0091 (0.0168)	0.59	0.0375 (0.0237)	0.11	0.0105 (0.0185)	0.57	0.0001 (0.0191)	1	0.0284 (0.0184)	0.12
5y AUROC	0.0239 (0.0174)	0.17	0.0315 (0.0197)	0.11	-0.0008 (0.0161)	0.96	0.0131 (0.0176)	0.46	0.0091 (0.0157)	0.56
10y AUROC	0.0200 (0.0170)	0.24	0.0156 (0.0191)	0.41	-0.0405 (0.0202)	0.04	-0.0070 (0.0199)	0.72	0.0216 (0.0157)	0.17
Model 3 vs. Model 2										
C-index (10y)	**-0.1480 (0.0297)**	**<0.01**	**-0.1575 (0.0300)**	**<0.01**	**-0.2217 (0.0310)**	**<0.01**	**-0.1224 (0.0246)**	**<0.01**	**-0.1708 (0.0299)**	**<0.01**
1y AUROC	**-0.2344 (0.0491)**	**<0.01**	**-0.1701 (0.0567)**	**<0.01**	**-0.1876 (0.0597)**	**<0.01**	**-0.1146 (0.0471)**	**0.01**	**-0.2060 (0.0676)**	**<0.01**
3y AUROC	**-0.1817 (0.0412)**	**<0.01**	**-0.1918 (0.0445)**	**<0.01**	**-0.2576 (0.0425)**	**<0.01**	**-0.1520 (0.0327)**	**<0.01**	**-0.1798 (0.0431)**	**<0.01**
5y AUROC	**-0.1455 (0.0381)**	**<0.01**	**-0.1728 (0.0375)**	**<0.01**	**-0.2351 (0.0382)**	**<0.01**	**-0.1356 (0.0305)**	**<0.01**	**-0.2100 (0.0366)**	**<0.01**
10y AUROC	**-0.1715 (0.0397)**	**<0.01**	**-0.1514 (0.0368)**	**<0.01**	**-0.2816 (0.0419)**	**<0.01**	**-0.1579 (0.0346)**	**<0.01**	**-0.1860 (0.0365)**	**<0.01**
Model 4 vs. Model 2										
C-index (10y)	0.0105 (0.0117)	0.37	0.0070 (0.0127)	0.58	**-0.0247 (0.0126)**	**<0.05**	-0.0117 (0.0112)	0.3	0.0032 (0.0123)	0.79
1y AUROC	-0.0276 (0.0168)	0.1	0.0226 (0.0200)	0.26	-0.0078 (0.0133)	0.56	0.0051 (0.0141)	0.72	-0.0005 (0.0318)	0.99
3y AUROC	0.0058 (0.0131)	0.65	0.0122 (0.0174)	0.48	-0.0132 (0.0158)	0.41	-0.0154 (0.0135)	0.26	0.0077 (0.0169)	0.65
5y AUROC	0.0118 (0.0154)	0.45	0.0097 (0.0163)	0.55	-0.0189 (0.0148)	0.2	-0.0071 (0.0132)	0.59	-0.0034 (0.0140)	0.81
10y AUROC	0.0082 (0.0162)	0.61	-0.0010 (0.0167)	0.95	**-0.0507 (0.0186)**	**<0.01**	-0.0335 (0.0166)	0.04	0.0051 (0.0138)	0.71
Model 4 vs. Model 3										
C-index (10y)	**0.1585 (0.0233)**	**<0.01**	**0.1646 (0.0228)**	**<0.01**	**0.1970 (0.0248)**	**<0.01**	**0.1108 (0.0186)**	**<0.01**	**0.1740 (0.0228)**	**<0.01**
1y AUROC	**0.2068 (0.0409)**	**<0.01**	**0.1927 (0.0453)**	**<0.01**	**0.1798 (0.0521)**	**<0.01**	**0.1197 (0.0392)**	**<0.01**	**0.2055 (0.0475)**	**<0.01**
3y AUROC	**0.1876 (0.0339)**	**<0.01**	**0.2040 (0.0345)**	**<0.01**	**0.2444 (0.0347)**	**<0.01**	**0.1366 (0.0253)**	**<0.01**	**0.1875 (0.0324)**	**<0.01**
5y AUROC	**0.1573 (0.0288)**	**<0.01**	**0.1825 (0.0280)**	**<0.01**	**0.2162 (0.0303)**	**<0.01**	**0.1285 (0.0231)**	**<0.01**	**0.2066 (0.0287)**	**<0.01**
10y AUROC	**0.1797 (0.0314)**	**<0.01**	**0.1505 (0.0268)**	**<0.01**	**0.2309 (0.0320)**	**<0.01**	**0.1244 (0.0241)**	**<0.01**	**0.1911 (0.0283)**	**<0.01**

Note: Bold denotes statistical significance (p<0.05).

Abbreviations: CSS, CRC-specific survival; AUROC, area under receiver operating characteristic curve; SE, standard error.

Supplementary Table 9. Performance and calibration of predictive models for CSS in each outer-loop of the total CRC cohort (N= 2116).

Models	Outer-loop	C-index (10y)	Time-dependent AUROC (95% CI)				IBS (10y)	Time-dependent Brier score (95% CI)			
			1y	3y	5y	10y		1y	3y	5y	10y
Model 1	1	0.75	0.90 (0.86-0.95)	0.84 (0.78-0.90)	0.80 (0.74-0.86)	0.80 (0.75-0.86)	0.13	0.05 (0.04-0.07)	0.11 (0.09-0.13)	0.15 (0.13-0.17)	0.19 (0.16-0.21)
	2	0.73	0.84 (0.76-0.91)	0.82 (0.75-0.88)	0.79 (0.73-0.85)	0.79 (0.74-0.85)	0.13	0.07 (0.05-0.08)	0.11 (0.10-0.13)	0.15 (0.13-0.17)	0.17 (0.15-0.20)
	3	0.8	0.87 (0.80-0.94)	0.86 (0.80-0.91)	0.85 (0.81-0.90)	0.85 (0.81-0.90)	0.12	0.05 (0.04-0.07)	0.10 (0.09-0.12)	0.13 (0.11-0.14)	0.17 (0.15-0.19)
	4	0.75	0.89 (0.83-0.94)	0.81 (0.75-0.87)	0.79 (0.74-0.85)	0.81 (0.75-0.86)	0.13	0.06 (0.04-0.07)	0.12 (0.10-0.14)	0.15 (0.13-0.17)	0.18 (0.16-0.20)
	5	0.8	0.89 (0.82-0.95)	0.84 (0.78-0.90)	0.85 (0.81-0.90)	0.86 (0.81-0.91)	0.12	0.05 (0.03-0.06)	0.10 (0.09-0.12)	0.13 (0.11-0.14)	0.17 (0.15-0.19)
Model 2	1	0.75	0.91 (0.87-0.95)	0.85 (0.79-0.91)	0.81 (0.75-0.87)	0.81 (0.76-0.87)	0.13	0.05 (0.04-0.07)	0.11 (0.09-0.12)	0.15 (0.13-0.17)	0.18 (0.16-0.21)
	2	0.75	0.88 (0.82-0.93)	0.84 (0.79-0.90)	0.81 (0.76-0.86)	0.81 (0.76-0.87)	0.12	0.06 (0.05-0.08)	0.11 (0.09-0.13)	0.14 (0.12-0.16)	0.17 (0.15-0.19)
	3	0.81	0.90 (0.84-0.95)	0.88 (0.83-0.93)	0.87 (0.83-0.92)	0.86 (0.82-0.91)	0.11	0.05 (0.04-0.07)	0.10 (0.08-0.11)	0.12 (0.11-0.14)	0.16 (0.15-0.18)
	4	0.77	0.89 (0.84-0.93)	0.83 (0.77-0.88)	0.81 (0.76-0.86)	0.83 (0.78-0.88)	0.13	0.06 (0.04-0.07)	0.12 (0.10-0.14)	0.14 (0.13-0.16)	0.17 (0.14-0.19)
	5	0.81	0.88 (0.81-0.94)	0.86 (0.81-0.92)	0.87 (0.82-0.91)	0.88 (0.83-0.92)	0.11	0.05 (0.04-0.06)	0.10 (0.08-0.11)	0.12 (0.10-0.14)	0.15 (0.13-0.17)
Model 3	1	0.61	0.67 (0.58-0.77)	0.66 (0.60-0.73)	0.67 (0.60-0.73)	0.64 (0.57-0.71)	0.17	0.07 (0.05-0.08)	0.15 (0.13-0.17)	0.19 (0.17-0.21)	0.25 (0.22-0.28)
	2	0.6	0.71 (0.62-0.79)	0.65 (0.58-0.72)	0.64 (0.57-0.70)	0.66 (0.59-0.73)	0.17	0.07 (0.05-0.09)	0.15 (0.13-0.17)	0.20 (0.18-0.22)	0.24 (0.21-0.27)
	3	0.58	0.71 (0.61-0.81)	0.62 (0.55-0.69)	0.64 (0.57-0.70)	0.58 (0.51-0.66)	0.16	0.06 (0.04-0.08)	0.15 (0.13-0.17)	0.18 (0.16-0.20)	0.25 (0.23-0.28)
	4	0.64	0.77 (0.69-0.85)	0.67 (0.61-0.74)	0.68 (0.62-0.74)	0.68 (0.61-0.74)	0.17	0.07 (0.05-0.08)	0.16 (0.14-0.18)	0.20 (0.18-0.22)	0.25 (0.22-0.28)
	5	0.65	0.67 (0.55-0.80)	0.68 (0.61-0.76)	0.66 (0.59-0.73)	0.69 (0.63-0.76)	0.16	0.05 (0.03-0.06)	0.13 (0.12-0.15)	0.18 (0.16-0.20)	0.24 (0.22-0.26)
Model 4	1	0.77	0.88 (0.82-0.94)	0.85 (0.80-0.91)	0.82 (0.77-0.87)	0.82 (0.77-0.88)	0.13	0.06 (0.05-0.08)	0.11 (0.09-0.13)	0.15 (0.13-0.17)	0.18 (0.15-0.21)
	2	0.76	0.90 (0.85-0.95)	0.85 (0.80-0.91)	0.82 (0.77-0.87)	0.81 (0.76-0.87)	0.13	0.06 (0.04-0.07)	0.11 (0.10-0.13)	0.14 (0.12-0.16)	0.17 (0.14-0.20)
	3	0.78	0.89 (0.84-0.94)	0.87 (0.82-0.92)	0.85 (0.81-0.90)	0.81 (0.76-0.87)	0.12	0.05 (0.04-0.06)	0.10 (0.09-0.12)	0.13 (0.11-0.15)	0.18 (0.15-0.20)
	4	0.75	0.89 (0.85-0.94)	0.81 (0.76-0.87)	0.81 (0.75-0.86)	0.80 (0.75-0.86)	0.13	0.06 (0.04-0.07)	0.12 (0.10-0.14)	0.15 (0.13-0.17)	0.18 (0.15-0.20)
	5	0.82	0.88 (0.80-0.95)	0.87 (0.82-0.92)	0.86 (0.82-0.91)	0.88 (0.84-0.93)	0.11	0.05 (0.04-0.07)	0.10 (0.09-0.12)	0.12 (0.11-0.14)	0.15 (0.13-0.17)

Note: Model 1 based on traditional clinical features, Model 2 based on comprehensive clinical features. Model 3 based on blood DNA methylation, Model 4 combined both clinical features and blood DNA methylation.
Abbreviations: CSS, CRC-specific survival; AUROC, area under receiver operating characteristic curve; CI, confidence interval; IBS, integrated Brier score.

Supplementary Table 10. Comparison of 10-year C-index and time-dependent AUROC between different predictive models for CSS in each outer-loop of subset 1 (N= 1042).

Models	Outer-loop 1		Outer-loop 2		Outer-loop 3		Outer-loop 4		Outer-loop 5	
	**ΔValue (SE)**	***p***	**ΔValue (SE)**	***p***	**ΔValue (SE)**	***p***	**ΔValue (SE)**	***p***	**ΔValue (SE)**	***p***
Model 2 vs. Model 1										
C-index (10y)	0.0229 (0.0193)	0.23	0.0102 (0.0170)	0.55	0.0115 (0.0164)	0.48	0.0165 (0.0164)	0.31	-0.0049 (0.0138)	0.72
1y AUROC	0.0136 (0.0356)	0.7	0.0340 (0.0304)	0.26	0.0396 (0.0427)	0.35	-0.0173 (0.0133)	0.19	-0.0095 (0.0237)	0.69
3y AUROC	0.0159 (0.0211)	0.45	0.0292 (0.0193)	0.13	0.0189 (0.0214)	0.38	0.0173 (0.0238)	0.47	0.0186 (0.0199)	0.35
5y AUROC	0.0340 (0.0231)	0.14	0.0224 (0.0207)	0.28	0.0171 (0.0190)	0.37	0.0209 (0.0232)	0.37	-0.0064 (0.0174)	0.71
10y AUROC	-0.0001 (0.0236)	1	-0.0026 (0.0211)	0.9	0.0149 (0.0193)	0.44	0.0408 (0.0247)	0.1	-0.0111 (0.0203)	0.59
Model 3 vs. Model 1										
C-index (10y)	**-0.1579 (0.0521)**	**<0.01**	**-0.1928 (0.0435)**	**<0.01**	**-0.2378 (0.0486)**	**<0.01**	-0.0833 (0.0457)	0.07	**-0.1659 (0.0415)**	**<0.01**
1y AUROC	**-0.2753 (0.0909)**	**<0.01**	**-0.2209 (0.0790)**	**<0.01**	**-0.1751 (0.0756)**	**0.02**	-0.1278 (0.0748)	0.09	**-0.2634 (0.0796)**	**<0.01**
3y AUROC	**-0.1611 (0.0783)**	**0.04**	**-0.2239 (0.0656)**	**<0.01**	**-0.2486 (0.0634)**	**<0.01**	**-0.1147 (0.0566)**	**0.04**	**-0.1822 (0.0587)**	**<0.01**
5y AUROC	**-0.1463 (0.0650)**	**0.02**	**-0.2106 (0.0535)**	**<0.01**	**-0.2499 (0.0575)**	**<0.01**	-0.0784 (0.0549)	0.15	**-0.2031 (0.0492)**	**<0.01**
10y AUROC	**-0.1689 (0.0635)**	**<0.01**	**-0.1516 (0.0606)**	**0.01**	**-0.2283 (0.0618)**	**<0.01**	-0.0910 (0.0606)	0.13	**-0.1452 (0.0521)**	**<0.01**
Model 4 vs. Model 1										
C-index (10y)	-0.0177 (0.0260)	0.5	-0.0185 (0.0239)	0.44	-0.0296 (0.0295)	0.32	0.0339 (0.0276)	0.22	-0.0096 (0.0190)	0.61
1y AUROC	-0.0580 (0.0519)	0.26	0.0167 (0.0318)	0.6	0.0219 (0.0384)	0.57	0.0052 (0.0296)	0.86	-0.0236 (0.0319)	0.46
3y AUROC	-0.0033 (0.0382)	0.93	0.0142 (0.0310)	0.65	0.0092 (0.0322)	0.78	0.0399 (0.0355)	0.26	-0.0077 (0.0239)	0.75
5y AUROC	-0.0115 (0.0318)	0.72	-0.0226 (0.0298)	0.45	-0.0026 (0.0318)	0.94	0.0447 (0.0339)	0.19	-0.0225 (0.0221)	0.31
10y AUROC	-0.0340 (0.0310)	0.27	-0.0169 (0.0357)	0.64	-0.0390 (0.0398)	0.33	0.0504 (0.0379)	0.18	-0.0060 (0.0235)	0.8
Model 3 vs. Model 2										
C-index (10y)	**-0.1808 (0.0527)**	**<0.01**	**-0.2031 (0.0437)**	**<0.01**	**-0.2493 (0.0468)**	**<0.01**	**-0.0997 (0.0423)**	**0.02**	**-0.1610 (0.0410)**	**<0.01**
1y AUROC	**-0.2889 (0.0931)**	**<0.01**	**-0.2550 (0.0723)**	**<0.01**	**-0.2146 (0.0833)**	**0.01**	-0.1105 (0.0735)	0.13	**-0.2539 (0.0916)**	**<0.01**
3y AUROC	**-0.1769 (0.0799)**	**0.03**	**-0.2531 (0.0649)**	**<0.01**	**-0.2674 (0.0601)**	**<0.01**	**-0.1320 (0.0508)**	**<0.01**	**-0.2008 (0.0572)**	**<0.01**
5y AUROC	**-0.1803 (0.0666)**	**<0.01**	**-0.2330 (0.0554)**	**<0.01**	**-0.2670 (0.0566)**	**<0.01**	**-0.0993 (0.0504)**	**<0.05**	**-0.1967 (0.0479)**	**<0.01**
10y AUROC	**-0.1689 (0.0654)**	**<0.01**	**-0.1491 (0.0616)**	**0.02**	**-0.2433 (0.0610)**	**<0.01**	**-0.1318 (0.0576)**	**0.02**	**-0.1341 (0.0509)**	**<0.01**
Model 4 vs. Model 2										
C-index (10y)	-0.0407 (0.0293)	0.16	-0.0287 (0.0247)	0.24	-0.0411 (0.0251)	0.1	0.0174 (0.0241)	0.47	-0.0047 (0.0192)	0.8
1y AUROC	-0.0716 (0.0511)	0.16	-0.0174 (0.0346)	0.62	-0.0176 (0.0154)	0.25	0.0225 (0.0265)	0.4	-0.0141 (0.0513)	0.78
3y AUROC	-0.0191 (0.0407)	0.64	-0.0151 (0.0315)	0.63	-0.0097 (0.0236)	0.68	0.0226 (0.0282)	0.42	-0.0263 (0.0244)	0.28
5y AUROC	-0.0455 (0.0375)	0.22	-0.0450 (0.0313)	0.15	-0.0196 (0.0257)	0.45	0.0238 (0.0287)	0.41	-0.0161 (0.0208)	0.44
10y AUROC	-0.0340 (0.0341)	0.32	-0.0143 (0.0342)	0.68	-0.0539 (0.0363)	0.14	0.0096 (0.0345)	0.78	0.0051 (0.0242)	0.83
Model 4 vs. Model 3										
C-index (10y)	**0.1402 (0.0369)**	**<0.01**	**0.1743 (0.0316)**	**<0.01**	**0.2083 (0.0373)**	**<0.01**	**0.1172 (0.0284)**	**<0.01**	**0.1563 (0.0319)**	**<0.01**
1y AUROC	**0.2173 (0.0743)**	**<0.01**	**0.2376 (0.0617)**	**<0.01**	**0.1970 (0.0745)**	**<0.01**	**0.1330 (0.0587)**	**0.02**	**0.2399 (0.0669)**	**<0.01**
3y AUROC	**0.1578 (0.0549)**	**<0.01**	**0.2380 (0.0491)**	**<0.01**	**0.2577 (0.0517)**	**<0.01**	**0.1545 (0.0347)**	**<0.01**	**0.1745 (0.0462)**	**<0.01**
5y AUROC	**0.1348 (0.0443)**	**<0.01**	**0.1880 (0.0386)**	**<0.01**	**0.2473 (0.0460)**	**<0.01**	**0.1231 (0.0335)**	**<0.01**	**0.1806 (0.0371)**	**<0.01**
10y AUROC	**0.1349 (0.0447)**	**<0.01**	**0.1348 (0.0377)**	**<0.01**	**0.1894 (0.0407)**	**<0.01**	**0.1414 (0.0347)**	**<0.01**	**0.1392 (0.0370)**	**<0.01**

Note: Subset 1 includes patients with blood samples collected ≥1-month after surgery. Bold denotes statistical significance (p<0.05).
Abbreviations: CSS, CRC-specific survival; AUROC, area under receiver operating characteristic curve; SE, standard error

Supplementary Table 11. Performance and calibration of predictive models for CSS in each outer-loop of subset 1 (N= 1042).

Models	Outer-loop	C-index (10y)	Time-dependent AUROC (95% CI)				IBS (10y)	Time-dependent Brier score (95% CI)			
			1y	3y	5y	10y		1y	3y	5y	10y
Model 1	1	0.75	0.90 (0.82-0.99)	0.83 (0.72-0.93)	0.77 (0.68-0.86)	0.80 (0.72-0.89)	0.13	0.04 (0.03-0.06)	0.10 (0.08-0.13)	0.16 (0.13-0.19)	0.21 (0.16-0.26)
	2	0.75	0.86 (0.75-0.97)	0.83 (0.74-0.92)	0.80 (0.72-0.87)	0.80 (0.71-0.88)	0.12	0.06 (0.04-0.08)	0.10 (0.08-0.12)	0.14 (0.11-0.16)	0.18 (0.15-0.21)
	3	0.81	0.86 (0.76-0.96)	0.88 (0.82-0.94)	0.88 (0.82-0.93)	0.84 (0.76-0.91)	0.11	0.04 (0.02-0.06)	0.09 (0.07-0.11)	0.11 (0.10-0.13)	0.18 (0.15-0.20)
	4	0.72	0.90 (0.83-0.98)	0.80 (0.71-0.89)	0.74 (0.65-0.84)	0.77 (0.68-0.86)	0.13	0.06 (0.03-0.08)	0.12 (0.09-0.15)	0.15 (0.12-0.17)	0.17 (0.14-0.20)
	5	0.79	0.90 (0.81-0.98)	0.81 (0.70-0.93)	0.84 (0.76-0.92)	0.87 (0.79-0.94)	0.11	0.04 (0.02-0.06)	0.10 (0.08-0.12)	0.12 (0.10-0.14)	0.17 (0.14-0.19)
Model 2	1	0.77	0.92 (0.85-0.98)	0.84 (0.74-0.94)	0.80 (0.72-0.89)	0.80 (0.71-0.89)	0.13	0.04 (0.03-0.06)	0.10 (0.08-0.13)	0.15 (0.13-0.18)	0.22 (0.16-0.27)
	2	0.75	0.89 (0.83-0.95)	0.86 (0.79-0.94)	0.82 (0.74-0.89)	0.80 (0.71-0.88)	0.12	0.06 (0.04-0.08)	0.10 (0.08-0.12)	0.13 (0.11-0.16)	0.18 (0.14-0.21)
	3	0.82	0.90 (0.80-1.00)	0.90 (0.84-0.96)	0.89 (0.84-0.95)	0.85 (0.78-0.93)	0.1	0.04 (0.02-0.06)	0.08 (0.07-0.10)	0.11 (0.09-0.13)	0.17 (0.14-0.19)
	4	0.75	0.88 (0.81-0.96)	0.82 (0.74-0.90)	0.77 (0.68-0.85)	0.81 (0.73-0.89)	0.13	0.06 (0.04-0.08)	0.12 (0.09-0.15)	0.15 (0.12-0.18)	0.16 (0.13-0.20)
	5	0.79	0.89 (0.82-0.96)	0.83 (0.73-0.93)	0.84 (0.76-0.91)	0.86 (0.78-0.93)	0.11	0.05 (0.03-0.06)	0.10 (0.07-0.12)	0.12 (0.10-0.15)	0.16 (0.13-0.19)
Model 3	1	0.59	0.63 (0.44-0.81)	0.66 (0.54-0.79)	0.62 (0.52-0.73)	0.63 (0.52-0.74)	0.15	0.05 (0.03-0.08)	0.12 (0.10-0.15)	0.17 (0.15-0.20)	0.24 (0.20-0.28)
	2	0.57	0.64 (0.51-0.77)	0.61 (0.50-0.72)	0.59 (0.49-0.68)	0.65 (0.54-0.75)	0.17	0.07 (0.04-0.10)	0.14 (0.12-0.17)	0.19 (0.16-0.22)	0.24 (0.19-0.28)
	3	0.57	0.68 (0.53-0.83)	0.63 (0.52-0.73)	0.63 (0.53-0.73)	0.61 (0.50-0.72)	0.16	0.05 (0.03-0.07)	0.13 (0.11-0.16)	0.18 (0.15-0.20)	0.25 (0.20-0.29)
	4	0.65	0.77 (0.65-0.90)	0.69 (0.60-0.77)	0.67 (0.58-0.76)	0.68 (0.57-0.78)	0.16	0.07 (0.04-0.09)	0.16 (0.13-0.19)	0.19 (0.16-0.22)	0.23 (0.19-0.28)
	5	0.63	0.63 (0.46-0.81)	0.63 (0.51-0.75)	0.64 (0.54-0.74)	0.72 (0.62-0.83)	0.16	0.05 (0.03-0.07)	0.13 (0.11-0.16)	0.19 (0.16-0.21)	0.22 (0.18-0.26)
Model 4	1	0.73	0.84 (0.72-0.97)	0.82 (0.73-0.92)	0.76 (0.67-0.85)	0.77 (0.68-0.86)	0.14	0.05 (0.03-0.07)	0.11 (0.08-0.13)	0.16 (0.13-0.19)	0.21 (0.15-0.26)
	2	0.73	0.87 (0.79-0.96)	0.85 (0.76-0.93)	0.77 (0.68-0.86)	0.78 (0.69-0.87)	0.13	0.05 (0.03-0.07)	0.11 (0.09-0.14)	0.15 (0.12-0.18)	0.19 (0.14-0.25)
	3	0.77	0.88 (0.78-0.98)	0.89 (0.81-0.96)	0.87 (0.81-0.94)	0.80 (0.71-0.89)	0.11	0.04 (0.03-0.06)	0.09 (0.07-0.11)	0.11 (0.09-0.14)	0.18 (0.13-0.23)
	4	0.76	0.91 (0.85-0.97)	0.84 (0.77-0.91)	0.79 (0.71-0.87)	0.82 (0.74-0.90)	0.12	0.05 (0.03-0.07)	0.12 (0.09-0.15)	0.15 (0.12-0.18)	0.17 (0.13-0.20)
	5	0.79	0.87 (0.75-1.00)	0.81 (0.69-0.92)	0.82 (0.74-0.90)	0.86 (0.78-0.94)	0.12	0.05 (0.03-0.07)	0.10 (0.08-0.13)	0.13 (0.10-0.15)	0.16 (0.12-0.20)

Note: Subset 1 includes patients with blood samples collected ≥1-month after surgery. Model 1 based on traditional clinical features, Model 2 based on comprehensive clinical features. Model 3 based on blood DNA methylation, Model 4 combined both clinical features and blood DNA methylation.
Abbreviations: CSS, CRC-specific survival; AUROC, area under receiver operating characteristic curve; CI, confidence interval; IBS, integrated Brier score.

Supplementary Table 12. Comparison of 10-year C-index and time-dependent AUROC between different predictive models for CSS in each outer-loop of subset 2 (N= 1434).

**Models**	**Outer-loop 1**		**Outer-loop 2**		**Outer-loop 3**		**Outer-loop 4**		**Outer-loop 5**	
	**ΔValue (SE)**	***p***	**ΔValue (SE)**	***p***	**ΔValue (SE)**	***p***	**ΔValue (SE)**	***p***	**ΔValue (SE)**	***p***
Model 2 vs. Model 1										
C-index (10y)	0.0097 (0.0234)	0.68	0.0260 (0.0255)	0.31	0.0330 (0.0234)	0.16	0.0050 (0.0162)	0.76	0.0388 (0.0220)	0.08
1y AUROC	-0.0077 (0.0504)	0.88	0.0816 (0.0800)	0.31	0.0875 (0.0765)	0.25	-0.0170 (0.0278)	0.54	0.0417 (0.0651)	0.52
3y AUROC	-0.0102 (0.0322)	0.75	0.0395 (0.0396)	0.32	**0.0840 (0.0375)**	**0.03**	-0.0004 (0.0224)	0.99	0.0678 (0.0401)	0.09
5y AUROC	0.0210 (0.0292)	0.47	0.0287 (0.0321)	0.37	0.0670 (0.0302)	0.03	0.0139 (0.0201)	0.49	0.0419 (0.0275)	0.13
10y AUROC	0.0263 (0.0291)	0.37	0.0268 (0.0273)	0.33	0.0192 (0.0263)	0.47	0.0038 (0.0231)	0.87	0.0397 (0.0243)	0.1
Model 3 vs. Model 1										
C-index (10y)	-0.0057 (0.0373)	0.88	-0.0280 (0.0367)	0.45	**-0.0849 (0.0388)**	**0.03**	-0.0548 (0.0377)	0.15	-0.0076 (0.0378)	0.84
1y AUROC	-0.1076 (0.0624)	0.08	-0.0098 (0.0666)	0.88	-0.0698 (0.0622)	0.26	-0.0810 (0.0765)	0.29	-0.0818 (0.0662)	0.22
3y AUROC	-0.0632 (0.0552)	0.25	-0.0273 (0.0632)	0.67	-0.0344 (0.0641)	0.59	-0.0858 (0.0523)	0.1	0.0503 (0.0533)	0.35
5y AUROC	-0.0218 (0.0479)	0.65	-0.0421 (0.0482)	0.38	-0.0803 (0.0511)	0.12	-0.0487 (0.0465)	0.29	-0.0415 (0.0470)	0.38
10y AUROC	-0.0151 (0.0482)	0.75	-0.0478 (0.0467)	0.31	**-0.1592 (0.0485)**	**<0.01**	**-0.1174 (0.0515)**	**0.02**	0.0073 (0.0482)	0.88
Model 4 vs. Model 1										
C-index (10y)	0.0079 (0.0270)	0.77	-0.0252 (0.0321)	0.43	**-0.0604 (0.0304)**	**<0.05**	-0.0316 (0.0297)	0.29	0.0231 (0.0247)	0.35
1y AUROC	-0.0789 (0.0501)	0.12	0.0028 (0.0651)	0.97	-0.0498 (0.0535)	0.35	-0.0190 (0.0581)	0.74	-0.0535 (0.0483)	0.27
3y AUROC	-0.0224 (0.0393)	0.57	-0.0093 (0.0580)	0.87	-0.0051 (0.0488)	0.92	-0.0530 (0.0409)	0.19	**0.0708 (0.0357)**	**<0.05**
5y AUROC	0.0065 (0.0332)	0.84	-0.0302 (0.0423)	0.47	-0.0323 (0.0382)	0.4	-0.0309 (0.0369)	0.4	0.0130 (0.0311)	0.67
10y AUROC	0.0231 (0.0349)	0.51	-0.0393 (0.0395)	0.32	**-0.1256 (0.0401)**	**<0.01**	-0.0801 (0.0412)	0.05	0.0240 (0.0301)	0.43
Model 3 vs. Model 2										
C-index (10y)	-0.0154 (0.0390)	0.69	-0.0540 (0.0390)	0.17	**-0.1179 (0.0428)**	**<0.01**	-0.0598 (0.0334)	0.07	-0.0464 (0.0411)	0.26
1y AUROC	-0.0999 (0.0800)	0.21	-0.0914 (0.0952)	0.34	**-0.1574 (0.0965)**	**0.1**	-0.0641 (0.0649)	0.32	-0.1235 (0.0912)	0.18
3y AUROC	-0.0529 (0.0546)	0.33	-0.0668 (0.0609)	0.27	-0.1184 (0.0671)	0.08	-0.0854 (0.0449)	0.06	-0.0176 (0.0645)	0.79
5y AUROC	-0.0428 (0.0501)	0.39	-0.0708 (0.0511)	0.17	**-0.1473 (0.0558)**	**<0.01**	-0.0626 (0.0406)	0.12	-0.0835 (0.0510)	0.1
10y AUROC	-0.0414 (0.0506)	0.41	-0.0746 (0.0478)	0.12	**-0.1784 (0.0515)**	**<0.01**	**-0.1213 (0.0492)**	**0.01**	-0.0324 (0.0511)	0.53
Model 4 vs. Model 2										
C-index (10y)	-0.0018 (0.0296)	0.95	-0.0511 (0.0337)	0.13	**-0.0934 (0.0335)**	**<0.01**	-0.0366 (0.0256)	0.15	-0.0157 (0.0277)	0.57
1y AUROC	-0.0712 (0.0692)	0.3	-0.0788 (0.0947)	0.41	-0.1373 (0.0861)	0.11	-0.0020 (0.0471)	0.97	-0.0951 (0.0792)	0.23
3y AUROC	-0.0122 (0.0394)	0.76	-0.0488 (0.0538)	0.36	-0.0890 (0.0503)	0.08	-0.0526 (0.0337)	0.12	0.0030 (0.0478)	0.95
5y AUROC	-0.0145 (0.0375)	0.7	-0.0589 (0.0445)	0.19	**-0.0994 (0.0427)**	**0.02**	-0.0448 (0.0310)	0.15	-0.0289 (0.0334)	0.39
10y AUROC	-0.0032 (0.0385)	0.93	-0.0661 (0.0399)	0.1	**-0.1448 (0.0416)**	**<0.01**	-0.0839 (0.0385)	0.03	-0.0157 (0.0308)	0.61
Model 4 vs. Model 3										
C-index (10y)	0.0136 (0.0172)	0.43	0.0028 (0.0113)	0.8	0.0245 (0.0159)	0.12	0.0232 (0.0146)	0.11	0.0307 (0.0229)	0.18
1y AUROC	0.0287 (0.0378)	0.45	0.0126 (0.0182)	0.49	0.0200 (0.0281)	0.48	**0.0621 (0.0281)**	**0.03**	0.0284 (0.0540)	0.6
3y AUROC	0.0407 (0.0276)	0.14	0.0179 (0.0149)	0.23	0.0294 (0.0254)	0.25	0.0328 (0.0213)	0.12	0.0206 (0.0279)	0.46
5y AUROC	0.0283 (0.0218)	0.19	0.0119 (0.0139)	0.39	**0.0480 (0.0208)**	**0.02**	0.0178 (0.0191)	0.35	0.0546 (0.0295)	0.06
10y AUROC	0.0382 (0.0218)	0.08	0.0086 (0.0182)	0.64	0.0336 (0.0198)	0.09	0.0374 (0.0201)	0.06	0.0167 (0.0317)	0.6

Note: Subset 2 includes patients with TNM stage II-III. Bold denotes statistical significance (p<0.05).

Abbreviations: CSS, CRC-specific survival; AUROC, area under receiver operating characteristic curve; SE, standard error

Supplementary Table 13. Performance and calibration of predictive models for CSS in each outer-loop of subset 2 (N= 1434).

Models	Outer-loop	C-index (10y)	Time-dependent AUROC (95% CI)				IBS (10y)	Time-dependent Brier score (95% CI)			
			1y	3y	5y	10y		1y	3y	5y	10y
Model 1	1	0.61	0.85 (0.78-0.92)	0.71 (0.61-0.82)	0.66 (0.56-0.75)	0.67 (0.58-0.76)	0.14	0.03 (0.02-0.05)	0.10 (0.08-0.12)	0.16 (0.14-0.18)	0.22 (0.19-0.24)
	2	0.57	0.70 (0.51-0.89)	0.65 (0.54-0.77)	0.63 (0.54-0.72)	0.64 (0.55-0.73)	0.13	0.03 (0.02-0.05)	0.10 (0.07-0.12)	0.16 (0.13-0.18)	0.21 (0.18-0.24)
	3	0.63	0.73 (0.55-0.90)	0.62 (0.52-0.72)	0.66 (0.57-0.75)	0.68 (0.60-0.77)	0.13	0.03 (0.01-0.05)	0.10 (0.08-0.12)	0.14 (0.12-0.16)	0.22 (0.19-0.24)
	4	0.64	0.87 (0.78-0.95)	0.68 (0.58-0.77)	0.64 (0.56-0.72)	0.71 (0.63-0.79)	0.15	0.04 (0.02-0.06)	0.13 (0.10-0.15)	0.17 (0.15-0.20)	0.22 (0.19-0.24)
	5	0.67	0.78 (0.61-0.94)	0.65 (0.52-0.77)	0.70 (0.61-0.79)	0.72 (0.63-0.80)	0.12	0.02 (0.01-0.03)	0.09 (0.07-0.11)	0.14 (0.12-0.16)	0.22 (0.19-0.24)
Model 2	1	0.62	0.84 (0.77-0.92)	0.70 (0.60-0.81)	0.68 (0.59-0.77)	0.70 (0.61-0.78)	0.14	0.03 (0.02-0.05)	0.10 (0.08-0.12)	0.16 (0.13-0.18)	0.21 (0.19-0.24)
	2	0.59	0.78 (0.62-0.94)	0.69 (0.59-0.80)	0.66 (0.57-0.74)	0.67 (0.58-0.76)	0.13	0.03 (0.02-0.05)	0.10 (0.08-0.12)	0.16 (0.13-0.18)	0.21 (0.17-0.24)
	3	0.66	0.81 (0.67-0.96)	0.70 (0.60-0.80)	0.73 (0.64-0.82)	0.70 (0.62-0.79)	0.12	0.03 (0.01-0.04)	0.10 (0.08-0.11)	0.14 (0.12-0.15)	0.21 (0.18-0.23)
	4	0.64	0.85 (0.78-0.92)	0.68 (0.59-0.77)	0.66 (0.58-0.74)	0.72 (0.64-0.80)	0.15	0.04 (0.02-0.06)	0.13 (0.11-0.16)	0.18 (0.15-0.20)	0.21 (0.18-0.24)
	5	0.71	0.82 (0.65-0.99)	0.72 (0.61-0.83)	0.74 (0.66-0.82)	0.76 (0.67-0.84)	0.11	0.02 (0.01-0.03)	0.08 (0.06-0.10)	0.13 (0.11-0.15)	0.20 (0.18-0.22)
Model 3	1	0.61	0.74 (0.61-0.88)	0.65 (0.56-0.74)	0.63 (0.55-0.72)	0.65 (0.57-0.74)	0.15	0.04 (0.02-0.05)	0.11 (0.09-0.14)	0.18 (0.15-0.20)	0.25 (0.21-0.29)
	2	0.55	0.69 (0.52-0.86)	0.63 (0.51-0.74)	0.58 (0.48-0.69)	0.60 (0.50-0.69)	0.17	0.04 (0.02-0.06)	0.13 (0.10-0.16)	0.19 (0.16-0.22)	0.26 (0.21-0.30)
	3	0.54	0.66 (0.49-0.82)	0.58 (0.46-0.70)	0.58 (0.48-0.68)	0.53 (0.43-0.62)	0.15	0.04 (0.02-0.05)	0.12 (0.09-0.14)	0.17 (0.15-0.20)	0.28 (0.24-0.32)
	4	0.58	0.78 (0.68-0.89)	0.59 (0.50-0.68)	0.60 (0.51-0.68)	0.60 (0.51-0.69)	0.19	0.05 (0.03-0.07)	0.17 (0.14-0.20)	0.22 (0.19-0.25)	0.29 (0.24-0.33)
	5	0.68	0.69 (0.49-0.90)	0.70 (0.58-0.82)	0.66 (0.56-0.76)	0.72 (0.64-0.80)	0.12	0.02 (0.01-0.04)	0.09 (0.07-0.11)	0.14 (0.12-0.16)	0.21 (0.19-0.24)
Model 4	1	0.62	0.77 (0.65-0.90)	0.69 (0.60-0.78)	0.66 (0.58-0.75)	0.69 (0.61-0.78)	0.15	0.04 (0.02-0.05)	0.11 (0.09-0.14)	0.18 (0.15-0.20)	0.23 (0.19-0.27)
	2	0.55	0.70 (0.53-0.87)	0.65 (0.53-0.76)	0.60 (0.50-0.69)	0.60 (0.51-0.70)	0.17	0.04 (0.02-0.06)	0.14 (0.11-0.16)	0.20 (0.17-0.23)	0.26 (0.21-0.31)
	3	0.56	0.68 (0.50-0.85)	0.61 (0.51-0.72)	0.63 (0.53-0.72)	0.56 (0.46-0.65)	0.14	0.04 (0.02-0.05)	0.11 (0.09-0.14)	0.16 (0.14-0.19)	0.26 (0.22-0.29)
	4	0.6	0.85 (0.78-0.92)	0.62 (0.53-0.72)	0.61 (0.53-0.70)	0.63 (0.55-0.72)	0.18	0.05 (0.03-0.06)	0.16 (0.13-0.19)	0.22 (0.19-0.25)	0.27 (0.23-0.32)
	5	0.7	0.72 (0.49-0.96)	0.72 (0.61-0.83)	0.71 (0.62-0.80)	0.74 (0.66-0.82)	0.12	0.02 (0.01-0.03)	0.09 (0.07-0.11)	0.14 (0.12-0.16)	0.21 (0.17-0.24)

Note: Subset 2 includes patients with TNM stage II-III. Model 1 based on traditional clinical features, Model 2 based on comprehensive clinical features. Model 3 based on blood DNA methylation, Model 4 combined both clinical features and blood DNA methylation.
Abbreviations: CSS, CRC-specific survival; AUROC, area under receiver operating characteristic curve; CI, confidence interval; IBS, integrated Brier score.

Supplementary Table 14. Number of patients in leave-sites-out 5-fold nested cross-validation

Groups	Total (%)	Subset 1 (%) [a]	Subset 2 (%) [b]
N	2116	1042	1434
Group 1	426 (20.1)	204 (19.6)	275 (19.2)
Group 2	427 (20.2)	207 (19.9)	278 (19.4)
Group 3	420 (19.8)	233 (22.4)	296 (20.6)
Group 4	431 (20.4)	204 (19.6)	284 (19.8)
Group 5	412 (19.5)	194 (18.6)	301 (21.0)

[a] Subset 1 includes patients with blood samples collected ≥1-month after surgery.
[b] Subset 2 includes patients with TNM stage II-III.
Note: Each group includes four hospitals and institutions.

Supplementary Table 15. Characteristics of comprehensive clinical features.

**Variable**	**Level**	**Total**	**Subset 1** [a]	***p***	**Subset 2** [b]	***p***
N	-	2116	1042	-	1434	-
Age at diagnosis (year, median [IQR])	-	69 [62, 77]	69 [62, 76]	0·21	70 [62, 77]	0·38
Age at blood collection (year median [IQR])	-	70 [63, 77]	70 [62, 77]	0·83	70·0 [63, 77]	0·38
Sex (female, %)	Female	869 (41·1)	399 (38·3)	0·15	612 (42·7)	0·36
T stage (%)	T1	124 (5·9)	72 (6·9)	0·60	12 (0·8)	<0·001
	T2	365 (17·2)	190 (18·3)		89 (6·2)	
	T3	1340 (63·3)	656 (63·1)		1155 (80·5)	
	T4	269 (12·7)	115 (11·1)		174 (12·1)	
	TX	14 (0·7)	6 (0·6)		4 (0·3)	
N stage (%)	N0	1145 (54·1)	576 (55·5)	0·92	738 (51·5)	<0·001
	N1	493 (23·3)	244 (23·5)		405 (28·2)	
	N2	446 (21·1)	207 (19·9)		290 (20·2)	
	N3	1 (0·0)	0 (0·0)		1 (0·1)	
	NX	25 (1·2)	11 (1·1)		0 (0·0)	
M stage (%)	M0	1294 (61·2)	626 (61·5)	0·15	1014 (70·7)	<0·001
	M1	291 (13·8)	122 (12·0)		0 (0·0)	
	MX	481 (22·7)	270 (26·5)		377 (26·3)	
Tumor location (%)	Distal colon	712 (33·6)	369 (35·5)	0·44	487 (34·0)	0·44
	Proximal colon	766 (36·2)	381 (36·7)		549 (38·3)	
	Rectum	635 (30·0)	289 (27·8)		396 (27·6)	
First-degree family history of colorectal cancer (%)	Yes	89 (4·2)	48 (4·6)	0·67	59 (4·1)	0·96
Charlson comorbidity index (median [IQR])	-	0·0 [0·0, 1·0]	0·0 [0·0, 1·0]	0·19	0·0 [0·0, 1·0]	0·49
BMI (kg/m^2, median [IQR])	-	26·1 [23·6, 29·0]	26·20 [23·7, 28·9]	0·99	26·1 [23·7, 29·0]	0·99
Smoking status (%)	Never	863 (40·8)	385 (37·0)	0·23	608 (42·4)	0·76
	Former	915 (43·2)	482 (46·3)		607 (42·3)	
	Current	336 (15·9)	174 (16·7)		217 (15·1)	
Pack years of smoking (median [IQR])	-	2·0 [0·0, 17·0]	3·0 [0·0, 19·0]	0·11	1·0 [0·0, 17·2]	0·50

[a] Subset 1 includes patients with blood samples collected ≥1-month after surgery.
[b] Subset 2 includes patients with TNM stage II-III.
Note: Distributions of total patients and each subset were compared with Pearson’s chi-square test or Kruskal–Wallis test.
Abbreviations: IQR, interquartile range; CI, confidence interval; BMI, body mass index; MET-h/week, metabolic equivalent hours/week; CRC, colorectal cancer; MSI, microsatellite instability; CIMP, CpG island methylator phenotype; CEA, carcinoembryonic antigen; Mut, mutation; Wt, wild-type.

Supplementary Table 15. Characteristics of comprehensive clinical features (continue).

Variable	Level	Total	Subset 1 [a]	*p*	Subset 2 [b]	*p*
Alcohol consumption (g/day, median [IQR])	-	8·9 [2·0, 22·5]	9·3 [2·0, 22·0]	0·53	8·7 [2·0, 22·4]	0·82
Average lifetime physical activity (ET-h/wk, median [IQR])	-	190·7 [130·2, 278·3]	181·0 [121·2, 262·6]	0·006	189·2 [130·9, 275·1]	0·74
Diet quality score (median [IQR])	-	25·0 [21·0, 29·0]	25·0 [21·0, 29·0]	0·71	25·0 [21·0, 29·0]	0·78
Education (%)	≤9y	1456 (68·8)	708 (68·14)	0·76	1033 (69·75)	0·79
	10-11y	347 (16·4)	166 (16·0)		248 (16·8)	
	≥12y	309 (14·6)	165 (15·9)		200 (13·5)	
Occupational position (%)	Junior	878 (41·5)	409 (45·1)	0·66	611 (42·6)	0·92
	Middle	826 (39·0)	424 (46·8)		548 (38·2)	
	Senior	140 (6·6)	74 (8·2)		91 (6·3)	
Employment situation (%)	Employed full time	290 (13·7)	148 (14·2)	0·95	190 (13·2)	1
	Employed part time	82 (3·9)	40 (3·8)		55 (3·8)	
	Self-employed	51 (2·4)	25 (2·4)		34 (2·4)	
	Housewife/ -husband	197 (9·3)	91 (8·7)		130 (9·1)	
	Retirement/early retirement	1458 (68·9)	714 (68·5)		995 (69·4)	
	Unemployed	25 (1·2)	15 (1·4)		20 (1·4)	
	Other	13 (0·6)	9 (0·9)		9 (0·6)	
MSI (Instability vs. stability, %)	Instability	209 (9·9)	104 (11·2)	0·69	175 (12·2)	0·08
KRAS (Mut vs. Wt, %)	Mut	634 (30·0)	334 (34·4)	0·39	434 (30·3)	0·87
BRAF (Mut vs. Wt, %)	Mut	159 (7·5)	85 (8·8)	0·65	111 (7·7)	0·97
CIMP (%)	Negative	727 (34·4)	353 (34·2)	0·87	474 (33·1)	0·85
	Low	1022 (48·3)	496 (48·0)		704 (49·1)	
	High	348 (16·4)	184 (17·8)		241 (16·8)	

[a] Subset 1 includes patients with blood samples collected ≥1-month after surgery.
[b] Subset 2 includes patients with TNM stage II-III.
Note: Distributions of total patients and each subset were compared with Pearson's chi-square test or Kruskal–Wallis test.
Abbreviations: IQR, interquartile range; CI, confidence interval; BMI, body mass index; MET-h/week, metabolic equivalent hours/week; CRC, colorectal cancer; MSI, microsatellite instability; CIMP, CpG island methylator phenotype; CEA, carcinoembryonic antigen; Mut, mutation; Wt, wild-type.

Supplementary Table 15. Characteristics of comprehensive clinical features (continued).

Variable	Level	Total	Subset 1 [a]	*p*	Subset 2 [b]	*p*
Resection edge (%)	R0	1968 (93·0)	990 (97·0)	0·16	1367 (95·3)	0·01
	R1	55 (2·6)	22 (2·2)		31 (2·2)	
	R2	27 (1·3)	9 (0·9)		5 (0·3)	
Differential grading (%)	G1	43 (2·0)	24 (2·4)	0·89	17 (1·2)	0·44
	G2	1451 (68·6)	715 (70·4)		995 (69·4)	
	G3	556 (26·3)	274 (27·0)		378 (26·4)	
	G4	11 (0·5)	3 (0·3)		7 (0·5)	
Histological type (%)	Adeno-Ca, unspecified	1599 (75·6)	778 (74·7)	0·71	1055 (73·6)	0·84
	Adeno-Ca, mucus-forming	494 (23·3)	252 (24·2)		363 (25·3)	
	Squamous-Ca	3 (0·1)	0 (0·0)		2 (0·1)	
	Signet ring-Ca	1 (0·0)	8 (0·8)		1 (0·1)	
	Other type	13 (0·6)	1 (0·1)		8 (0·6)	
	Not specified	6 (0·3)	3 (0·3)		5 (0·3)	
Neo-chemotherapy (%)	Yes	121 (5·7)	65 (6·2)	0·62	95 (6·6)	0·15
Neo-radiotherapy (%)	Yes	180 (8·5)	90 (8·6)	0·96	143 (10·0)	0·15
Adjuvant-chemotherapy (%)	Yes	473 (22·4)	432 (41·5)	<0·001	352 (24·5)	0·31
Adjuvant-radiotherapy (%)	Yes	78 (3·7)	78 (7·5)	<0·001	71 (5·0)	0·18
Relapse (%)	Yes	15 (0·7)	15 (1·4)	0·1	10 (0·7)	0·50
Metastasis (%)	Yes	36 (1·7)	36 (3·5)	0·006	27 (1·9)	0·47
Surgery after relapse (%)	Yes	11 (0·5)	11 (1·1)	0·16	8 (0·6)	0·49
Chemotherapy after relapse (%)	Yes	7 (0·3)	7 (0·7)	0·28	2 (0·1)	0·27
Radiotherapy after relapse (%)	Yes	5 (0·2)	5 (0·5)	0·36	3 (0·2)	0·49
Surgery after metastasis (%)	Yes	14 (0·7)	14 (1·3)	0·11	12 (0·8)	0·42
Chemotherapy after metastasis (%)	Yes	31 (1·5)	31 (3·0)	0·011	22 (1·5)	0·49
Radiotherapy after metastasis (%)	Yes	2 (0·1)	2 (0·2)	0·54	2 (0·1)	0·46

[a] Subset 1 includes patients with blood samples collected ≥1-month after surgery.
[b] Subset 2 includes patients with TNM stage II-III.
Note: Distributions of total patients and each subset were compared with Pearson's chi-square test or Kruskal–Wallis test.
Abbreviations: IQR, interquartile range; CI, confidence interval; BMI, body mass index; MET-h/week, metabolic equivalent hours/week; CRC, colorectal cancer; MSI, microsatellite instability; CIMP, CpG island methylator phenotype; CEA, carcinoembryonic antigen; Mut, mutation; Wt, wild-type.

Section/Topic	Item	Development / evaluation[1]	Checklist item	Reported on page
**TITLE**				
Title	1	D;E	Identify the study as developing or evaluating the performance of a multivariable prediction model, the target population, and the outcome to be predicted	1
**ABSTRACT**				
Abstract	2	D;E	See TRIPOD+AI for Abstracts checklist	2
**INTRODUCTION**				
Background	3a	D;E	Explain the healthcare context (including whether diagnostic or prognostic) and rationale for developing or evaluating the prediction model, including references to existing models	3-4
	3b	D;E	Describe the target population and the intended purpose of the prediction model in the context of the care pathway, including its intended users (e.g., healthcare professionals, patients, public)	4
	3c	D;E	Describe any known health inequalities between sociodemographic groups	NA
Objectives	4	D;E	Specify the study objectives, including whether the study describes the development or validation of a prediction model (or both)	4
**METHODS**				
Data	5a	D;E	Describe the sources of data separately for the development and evaluation datasets (e.g., randomised trial, cohort, routine care or registry data), the rationale for using these data, and representativeness of the data	14
	5b	D;E	Specify the dates of the collected participant data, including start and end of participant accrual; and, if applicable, end of follow-up	14
Participants	6a	D;E	Specify key elements of the study setting (e.g., primary care, secondary care, general population) including the number and location of centres	14
	6b	D;E	Describe the eligibility criteria for study participants	14
	6c	D;E	Give details of any treatments received, and how they were handled during model development or evaluation, if relevant	14
Data preparation	7	D;E	Describe any data pre-processing and quality checking, including whether this was similar across relevant sociodemographic groups	15
Outcome	8a	D;E	Clearly define the outcome that is being predicted and the time horizon, including how and when assessed, the rationale for choosing this outcome, and whether the method of outcome assessment is consistent across sociodemographic groups	15-16
	8b	D;E	If outcome assessment requires subjective interpretation, describe the qualifications and demographic characteristics of the outcome assessors	NA
	8c	D;E	Report any actions to blind assessment of the outcome to be predicted	NA
Predictors	9a	D	Describe the choice of initial predictors (e.g., literature, previous models, all available predictors) and any pre-selection of predictors before model building	16-17
	9b	D;E	Clearly define all predictors, including how and when they were measured (and any actions to blind assessment of predictors for the outcome and other predictors)	16-17
	9c	D;E	If predictor measurement requires subjective interpretation, describe the qualifications and demographic characteristics of the predictor assessors	NA
Sample size	10	D;E	Explain how the study size was arrived at (separately for development and evaluation), and justify that the study size was sufficient to answer the research question. Include details of any sample size calculation	14
Missing data	11	D;E	Describe how missing data were handled. Provide reasons for omitting any data	17
Analytical methods	12a	D	Describe how the data were used (e.g., for development and evaluation of model performance) in the analysis, including whether the data were partitioned, considering any sample size requirements	17-18
	12b	D	Depending on the type of model, describe how predictors were handled in the analyses (functional form, rescaling, transformation, or any standardisation).	15-16
	12c	D	Specify the type of model, rationale[2], all model-building steps, including any hyperparameter tuning, and method for internal validation	16-17
	12d	D;E	Describe if and how any heterogeneity in estimates of model parameter values and model performance was handled and quantified across clusters (e.g., hospitals, countries). See TRIPOD-Cluster for additional considerations[3]	16
	12e	D;E	Specify all measures and plots used (and their rationale) to evaluate model performance (e.g., discrimination, calibration, clinical utility) and, if relevant, to compare multiple models	17-18
	12f	E	Describe any model updating (e.g., recalibration) arising from the model evaluation, either overall or for particular sociodemographic groups or settings	16-18
	12g	E	For model evaluation, describe how the model predictions were calculated (e.g., formula, code, object, application programming interface)	NA
Class imbalance	13	D;E	If class imbalance methods were used, state why and how this was done, and any subsequent methods to recalibrate the model or the model predictions	NA
Fairness	14	D;E	Describe any approaches that were used to address model fairness and their rationale	NA
Model output	15	D	Specify the output of the prediction model (e.g., probabilities, classification). Provide details and rationale for any classification and how the thresholds were identified	17-18

[1] D=items relevant only to the development of a prediction model; E=items relating solely to the evaluation of a prediction model; D;E=items applicable to both the development and evaluation of a prediction model

[2] Separately for all model building approaches.

[3] TRIPOD-Cluster is a checklist of reporting recommendations for studies developing or validating models that explicitly account for clustering or explore heterogeneity in model performance (eg, at different hospitals or centres). Debray et al, BMJ 2023; 380: e071018 [DOI: 10.1136/bmj-2022-071018]

Training versus evaluation	16	D;E	Identify any differences between the development and evaluation data in healthcare setting, eligibility criteria, outcome, and predictors	17-18
Ethical approval	17	D;E	Name the institutional research board or ethics committee that approved the study and describe the participant-informed consent or the ethics committee waiver of informed consent	15
**OPEN SCIENCE**				
Funding	18a	D;E	Give the source of funding and the role of the funders for the present study	20
Conflicts of interest	18b	D;E	Declare any conflicts of interest and financial disclosures for all authors	21
Protocol	18c	D;E	Indicate where the study protocol can be accessed or state that a protocol was not prepared	NA
Registration	18d	D;E	Provide registration information for the study, including register name and registration number, or state that the study was not registered	NA
Data sharing	18e	D;E	Provide details of the availability of the study data	19
Code sharing	18f	D;E	Provide details of the availability of the analytical code[4]	19
**PATIENT & PUBLIC INVOLVEMENT**				
Patient & Public Involvement	19	D;E	Provide details of any patient and public involvement during the design, conduct, reporting, interpretation, or dissemination of the study or state no involvement.	14
**RESULTS**				
Participants	20a	D;E	Describe the flow of participants through the study, including the number of participants with and without the outcome and, if applicable, a summary of the follow-up time. A diagram may be helpful.	5
	20b	D;E	Report the characteristics overall and, where applicable, for each data source or setting, including the key dates, key predictors (including demographics), treatments received, sample size, number of outcome events, follow-up time, and amount of missing data. A table may be helpful. Report any differences across key demographic groups.	5
	20c	E	For model evaluation, show a comparison with the development data of the distribution of important predictors (demographics, predictors, and outcome).	NA
Model development	21	D;E	Specify the number of participants and outcome events in each analysis (e.g., for model development, hyperparameter tuning, model evaluation)	5-6
Model specification	22	D	Provide details of the full prediction model (e.g., formula, code, object, application programming interface) to allow predictions in new individuals and to enable third-party evaluation and implementation, including any restrictions to access or re-use (e.g., freely available, proprietary)[5]	19
Model performance	23a	D;E	Report model performance estimates with confidence intervals, including for any key subgroups (e.g., sociodemographic). Consider plots to aid presentation.	5-7
	23b	D;E	If examined, report results of any heterogeneity in model performance across clusters. See TRIPOD Cluster for additional details[3].	5-7
Model updating	24	E	Report the results from any model updating, including the updated model and subsequent performance	NA
**DISCUSSION**				
Interpretation	25	D;E	Give an overall interpretation of the main results, including issues of fairness in the context of the objectives and previous studies	9
Limitations	26	D;E	Discuss any limitations of the study (such as a non-representative sample, sample size, overfitting, missing data) and their effects on any biases, statistical uncertainty, and generalizability	12-13
Usability of the model in the context of current care	27a	D	Describe how poor quality or unavailable input data (e.g., predictor values) should be assessed and handled when implementing the prediction model	NA
	27b	D	Specify whether users will be required to interact in the handling of the input data or use of the model, and what level of expertise is required of users	NA
	27c	D;E	Discuss any next steps for future research, with a specific view to applicability and generalizability of the model	12-13

From: Collins GS, Moons KGM, Dhiman P, et al. *BMJ* 2024;385:e078378. doi:10.1136/bmj-2023-078378

[4] This relates to the analysis code, for example, any data cleaning, feature engineering, model building, evaluation.
[5] This relates to the code to implement the model to get estimates of risk for a new individual.
